# Genetic analyses and prediction for lodging‑related traits in a diverse Iranian hexaploid wheat collection

**DOI:** 10.1038/s41598-023-49927-z

**Published:** 2024-01-02

**Authors:** Ehsan Rabieyan, Reza Darvishzadeh, Hadi Alipour

**Affiliations:** Department of Plant Production and Genetics, Urmia University, Urmia, Iran

**Keywords:** Genome-wide association studies, Plant breeding

## Abstract

Lodging is one of the most important limiting environmental factors for achieving the maximum yield and quality of grains in cereals, including wheat. However, little is known about the genetic foundation underlying lodging resistance (LR) in wheat. In this study, 208 landraces and 90 cultivars were phenotyped in two cropping seasons (2018–2019 and 2019–2020) for 19 LR-related traits. A genome-wide association study (GWAS) and genomics prediction were carried out to dissect the genomic regions of LR. The number of significant marker pairs (MPs) was highest for genome B in both landraces (427,017) and cultivars (37,359). The strongest linkage disequilibrium (LD) between marker pairs was found on chromosome 4A (0.318). For stem lodging-related traits, 465, 497, and 478 marker-trait associations (MTAs) and 45 candidate genes were identified in year 1, year 2, and pooled. Gene ontology exhibited genomic region on Chr. 2B, 6B, and 7B control lodging. Most of these genes have key roles in defense response, calcium ion transmembrane transport, carbohydrate metabolic process, nitrogen compound metabolic process, and some genes harbor unknown functions that, all together may respond to lodging as a complex network. The module associated with starch and sucrose biosynthesis was highlighted. Regarding genomic prediction, the GBLUP model performed better than BRR and RRBLUP. This suggests that GBLUP would be a good tool for wheat genome selection. As a result of these findings, it has been possible to identify pivotal QTLs and genes that could be used to improve stem lodging resistance in *Triticum aestivum* L.

## Introduction

Bread wheat (*Triticum aestivum* L.) is among the widely consumed food crops worldwide and is regarded as one of the most traded commodities on global markets^1,2^. Lodging is one of the most important limiting environmental factors for achieving the maximum yield and quality of grains in cereals, including wheat^3–5^. Lodging is when the roots of a crop are dislocated and/or their stems are irreversibly bent downward^4^. There are several difficulties that result from this situation, including higher drying costs, slowed harvest, reduced grain quality, and drastic yield losses of up to 85%^3,5,6^. The main challenge is the lack of global, regional, or local statistics on lodged areas related to various crops^7^. There are three key elements in determining the level of lodging and yield loss—the lodging angle, the spatial extent of lodging, and the stage of crop development (time of lodging incidence)^8^. By definition, CAI refers to the angles formed by stems with respect to vertical planes^9^. During lodging, a crop may experience a sequence of steps (i.e., lodging stages) beginning with CAI∼0° (a low deviation from the vertical situation) and finishing with CAI∼90° (crop bending close to the horizontal situation)^10^. Hence, CAI levels (ranging from moderate to severe) can be used to evaluate the lodging stage and canopy structure of lodged crops^11,12^. Agronomists and crop physiologists study lodging widely, but their efforts are usually limited to two aspects: agronomic practices (which reduce lodging-related risks) and breeding programs (which develop lodging-tolerant cultivars)^11^. There are four main characteristics of wheat ideotypes that demonstrate lodging resistance: larger stem diameters, wide root plates, strong root systems, and moderately short heights^13^.

There are many challenges associated with evaluating lodging level since there are no data associated with it, no standard scale is available to present it, and lodging distribution on farms is random, involving complex genetic-environmental interactions^10,14,15^. The physiology of wheat lodging is influenced by a complex genetic architecture^16,17^. A complex trait that is difficult to quantify in the field is lodging^18^. For these reasons, assessing a genetic panel for lodging tolerance is a difficult task for wheat breeders. To make further progress in the development of lodging-tolerant wheat varieties it is crucial to get a better understanding of the molecular basis of lodging tolerance-related traits by using genetic tools, like QTL (quantitative trait loci) mapping^19^. In this context, a handful of QTLs accounting for 2–27% of stem strength and lodging variation^20–22^ have been reported. The advent of next-generation sequencing (NGS) approaches has enabled cost-efficient genotyping-by sequencing that has been shown to be a useful tool by facilitating genetic dissection of complex traits in non-model organisms. Association mapping overcomes many of the restrictions of classic QTL mapping and can help identify minor genetic factors underlying complex traits^19^. QTLs identified through association mapping can be directly utilized in marker-assisted selection for improving genetic gain. To date, a few genome-wide association studies (GWAS) have been adopted to explore marker-trait associations (MTAs) and candidate genes affecting growth and development for lodging in crop plants including wheat^23^, oat^24^, bean^25^, canola^26,27^, and rice^28^. For example, Singh et al.^23^ explored lodging tolerance via GWAS and identified a key genomic region on chromosome 2A, consistent across digital and visual scores of the lodging.

Anatomically lodging resistance is directly related to plant height, all 21 chromosomes carry genes that control plant height in wheat^29–31^. Up to now, 24 reduced height (*Rht*) genes (*Rht1–Rht24*) are catalogued in wheat^32,33^, where *Rht8* on chromosome arm 2DS has been extensively explored^34,35^. We could locate only two QTLs to chromosome 2DL, whereas the ones reported by Borner et al^36^, on chromosome 2DS could not be detected.

Genomic prediction (GP) boosts speed and effectiveness of breeding by shortening breeding cycles and improving selection accuracy as auxiliary tools for GWAS^37,38^. An advantage of this approach is that it provides an opportunity to select a candidate gene by genotyping it before determining its phenotype^39^. Genomic prediction involves training a model that is comprised of all genetic markers within a model. A validation set is used to estimate the accuracy of the model^40^. Several factors affect genomic accuracy, including marker set, population structure, genomic selection method, and trait genetic architecture. Research has shown that GP is highly or moderately accurate for quantitative characteristics in barley^41^, maize^42^, rice^43^, oat^44^, and wheat^20,39^.

To the best of our knowledge, little is known about genomic regions associated with lodging resistance in wheat. Therefore, we uncovered putative candidate genes and evaluated the genomic prediction accuracy of lodging resistance using three methods for building a genomic selection model, namely genomic best linear unbiased prediction (GBLUP), ridge regression-best linear unbiased prediction (RRBLUP), and Bayesian ridge regression (BRR).

## Result

### Phenotypic variation and correlation analysis

According to the analysis of variance, genotypic, and genotype × environmental effects on lodging-related traits were significant (Supplementary Table S1). Grain yield and number of nodes showed the lowest and highest phenotypic coefficient of variation (PCV) and genotypic coefficient of variation (GCV). A low heritability was observed in grain yield (41.32%), and a high heritability was observed in number of nodes (88.25%). Descriptive data on the lodging-related traits of wheat genotypes can be seen in Supplementary Fig S1 and Supplementary Table S2. Cultivars and landraces showed an average crop angle of inclination (CAI), lodging area (LA), and lodging index (LS) of 69.3 and 79.2°, 64.4 and 100%, 0.49 and 0.84, respectively. Because of this, cultivars have lower lodging rates than native landraces. Compared to native populations, cultivars had lower plant height, IL1, IL2, PL, and PeL, and larger stem diameters at nodes. Landraces showed higher DTH, DTF, and DTM while cultivars showed lower DTH, DTF, and DTM. Furthermore, cultivars showed better spike weight, spike area, and grain yield than landraces.

**Figure 1:**
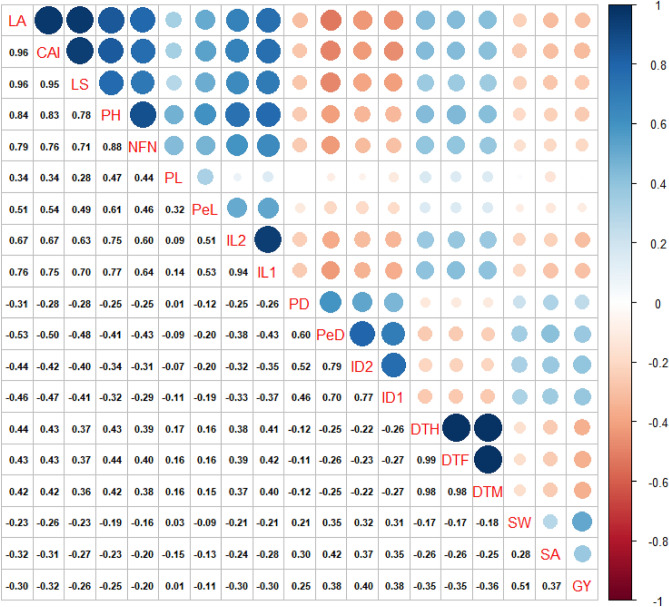
Correlation coefficients between the lodging-related traits in Iranian wheat cultivars and landraces. *LA* lodged area, *CAI* crop angle of inclination, *LS* lodging score index, *PH* plant height, *NFN* number of nodes, *PL* peduncle length, *PeL* penultimate length, *IL1* internode length 1, *IL2* internode length 2, *PD* peduncle diameter, *PeD* penultimate diameter, *ID1* internode diameter 1, *ID2* internode diameter 2, *DTH* days to heading, *DTF* days to flowering, *DTM* days to maturity, *SW* spike weight, *SA* spike area, *GY* grain yield.

Pearson correlation between lodging-related traits is illustrated in Fig. 1. The lodging index was most positively correlated with LA (r = 0.96**), followed by CAI (r = 0.95**), PH (r = 0.78**), NFN (r = 0.71**), IL1 (r = 0.70**), and IL2 (r = 0.63**). The lodging index also showed the greatest negative correlation with PeD (r = − 0.48**), followed by ID1 (r = − 0.41**) and ID2 (r = − 0.40**). These findings show that the higher the LI, the lower the grain yield (r = − 0.26**). Correlation coefficients between two environments (year 1 and year 2) for the lodging-related traits in Iranian wheat cultivars and landraces are shown in Supplementary Table S3.

**Figure 2:**
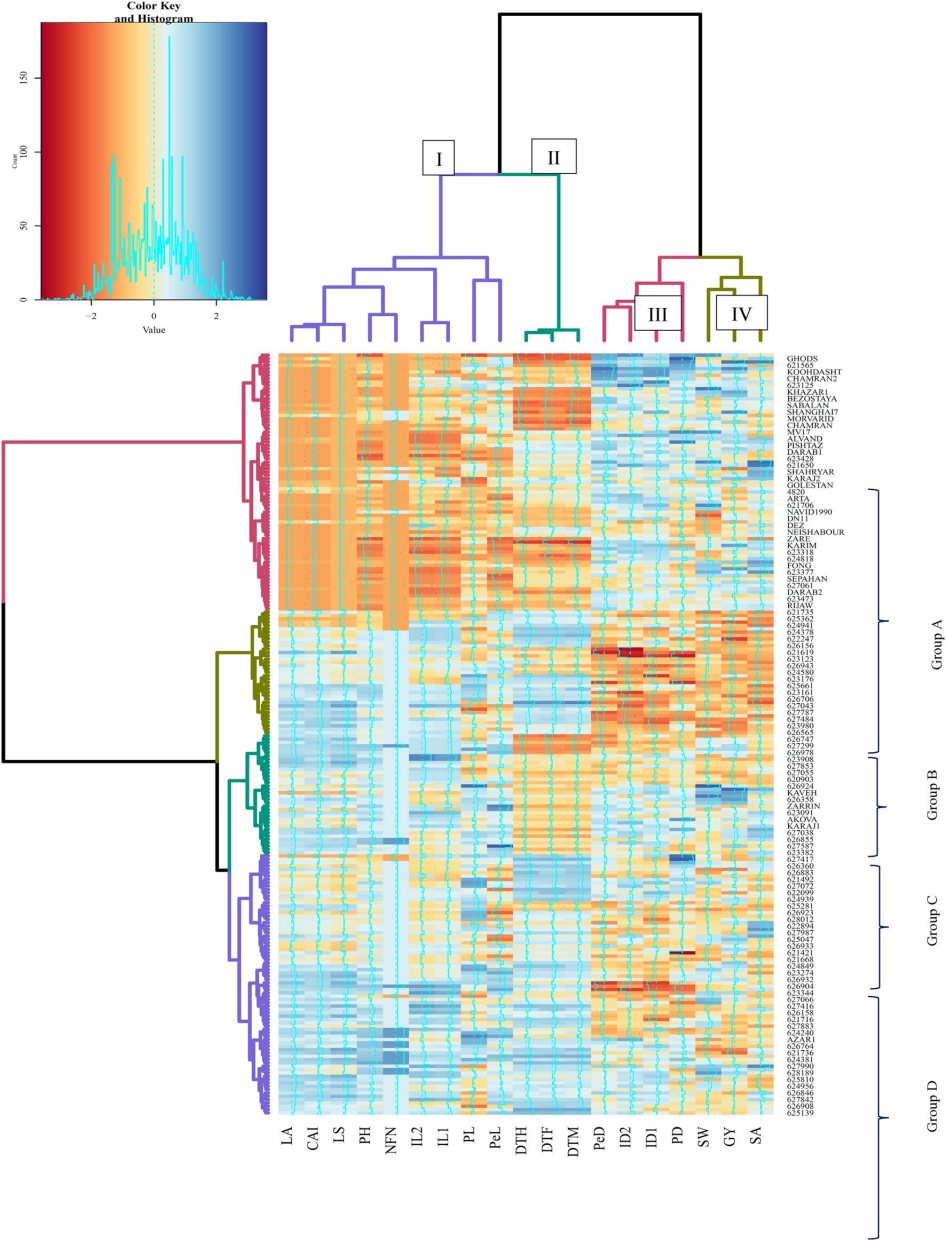
Hierarchical clustering and heatmap of Iranian wheat landraces and cultivars based on the wheat lodging-related traits. *LA* lodged area, *CAI* crop angle of inclination, *LS* lodging score index, *PH* plant height, *NFN* number of nodes, *PL* peduncle length, *PeL* penultimate length, *IL1* internode length 1, *IL2* internode length 2, *PD* peduncle diameter, *PeD* penultimate diameter, *ID1* internode diameter 1, *ID2* internode diameter 2, *DTH* days to heading, *DTF* days to flowering, *DTM* days to maturity, *SW* spike weight, *SA* spike area, *GY* grain yield.

Genotypes were divided into four bunches based on heatmap output. The foremost lodging-resistant genotypes were found within bunch A, which had a lodging index of zero or near zero. Genotypes with lodging index between 0 and 0.15% were found within bunch B. In the remaining two groups, genotypes were observed with a high lodging index. The lodging index within bunch D, which incorporates most local populaces, was the most elevated and extended from 0.6 to 1. Traits were separated into four groups: group 1 including SA, SW, and GY; Group 2 including PD, PeD, ID1, and ID2; Group 3 including DTH, DTF, and DTM; Group 4 including LS, CAI, LA, IL1, IL2, PH, NFN, PL, and PeL (Fig. 2).

### Marker distribution and linkage disequilibrium (LD)

We had used four different reference genomes including barley, Chinese Spring, W7984, and IWGSC RefSeq v1.0 for imputation and the results indicate that W7984 provides more accurate results than other reference genomes. Therefore, in the current study we only used the result that obtained from W7984 reference genome. An analysis of 566,439,207 unique reads was obtained by genotyping 298 Iranian bread wheat accessions. A total of 133,039 SNPs were identified after alignment and de-duplication, of which 10,938 had a MAF > 5%, heterozygosity < 10%, and missing data < 10%. The 10,938 SNPs were retained and used for imputation. Afterward, 43,525 imputed SNPs were used to conduct the association study. A, B, and D genomes were mapped with 15,951, 21,864, and 5,710 SNPs, respectively, representing 36.7%, 50.2%, and 13.1% of all SNPs. The lowest and highest number of SNPs were on 4D (270 SNPs) and 3A (4034 SNPs), respectively (Supplementary Fig. S2).

According to the LD calculation of 46,525 SNPs, 1,858,425 marker pairs (MPs) were found in the panel of cultivars, of which 37.72% showed significant linkage. A range of 0.138 (Chr. 3D) to 0.368 (Chr. 4A) LD between marker pairs was observed across the 21 chromosomes. The D genome had the lowest number of MPs (215,600, 11.60%), followed by the A genome (683,825, 36.80%) and the B genome (959,000, 51.60%) (Table S1). A similar test on wheat landraces revealed 1,867,575 MPs, which were lower than those in wheat cultivars with a mean r^2^ of 0.182. As expected, landraces showed a higher percentage of significant markers (847,725, 45.39%). The strongest LD was observed between marker pairs in Chr.4A (0.369), followed by Chr. 2A (0.289) (Supplementary Table S4).

### Population structure and Kinship matrix

**Figure 3:**
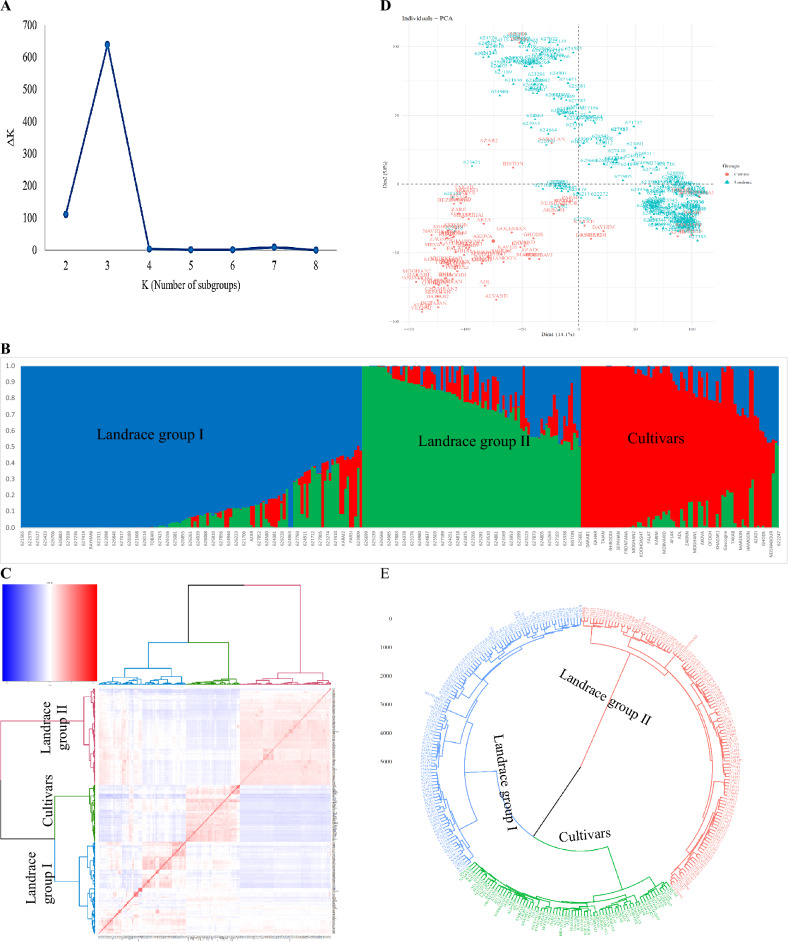
Determination of subpopulations number in wheat genotypes based on ΔK values (**A**), A structure plot of the 298 wheat genotypes and landraces determined by K = 3 (**B**). Principle component analysis (PCA) for a total of 298 Iranian bread wheat accessions (**C**). Cluster analysis using Kinship matrix of imputed data for Iranian wheat accessions (**D**). The dendrogram of Neighbor-Joining clustering was constructed using 43,525 SNPs and 298 Iranian wheat accessions (**E**).

According to the analyses of population structure, there are three subpopulations with varying degrees of mixing within them. The population structure matrix also revealed the maximum value of ΔK for K = 3, showing that the Iranian wheat genotypes can be divided into three subpopulations (Fig. 3A,B). A cluster analysis of the kinship matrix showed that the SBP-I subgroup contains 135 genotypes (13 cultivars and 122 landraces), the SBP-II contains 88 genotypes (5 cultivars and 63 landraces), and the SBP-III contains 75 genotypes (72 cultivars and 3 landraces) (Fig. 3C). In the principal component analysis, PC1 and PC2, explained 14.1 and 5.8 of the genotypic variation, respectively (Fig. 3D). Three distinct subpopulations were identified based on the first three PCs, with admixed accessions falling between the three major subpopulations. Additionally, the neighbor-joining tree of all accessions clearly showed that they were clustered into three subgroups (Fig. 3E).

### Genome-wide association analysis for lodging-related traits

**Figure 4:**
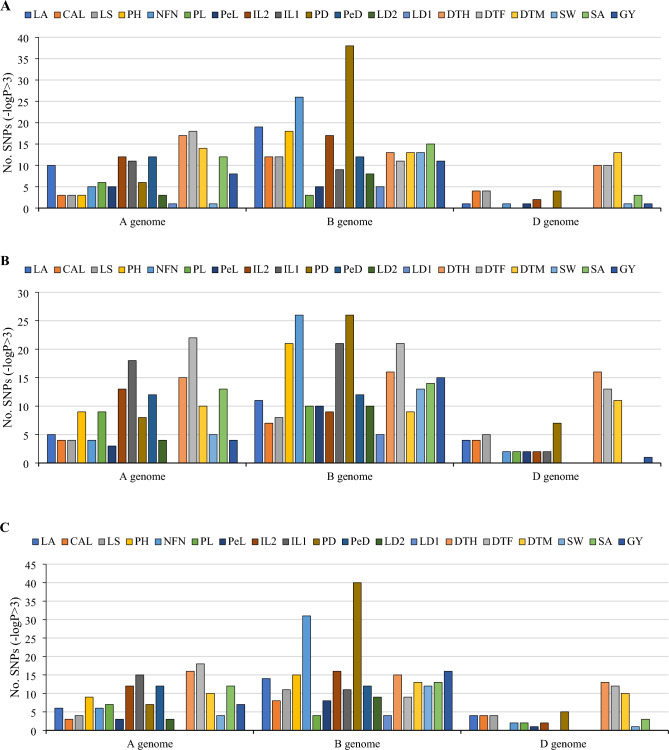
GWAS results (CMLM method) for lodging-related traits in Iranian landraces and cultivars (A = year 1, B = year 2, C = pooled). *GWAS* genome wide association study, *CMLM* compressed mixed linear model, *LA* lodged area, *CAI* crop angle of inclination, *LS* lodging score index, *PH* plant height, *NFN* number of nodes, *PL* peduncle length, *PeL* penultimate length, *IL1* internode length 1, *IL2* internode length 2, *PD* peduncle diameter, *PeD* penultimate diameter, *ID1* internode diameter 1, *ID2* internode diameter 2, *DTH* days to heading, *DTF* days to flowering, *DTM* days to maturity, *SW* spike weight, *SA* spike area, *GY* grain yield.

**Figure 5:**
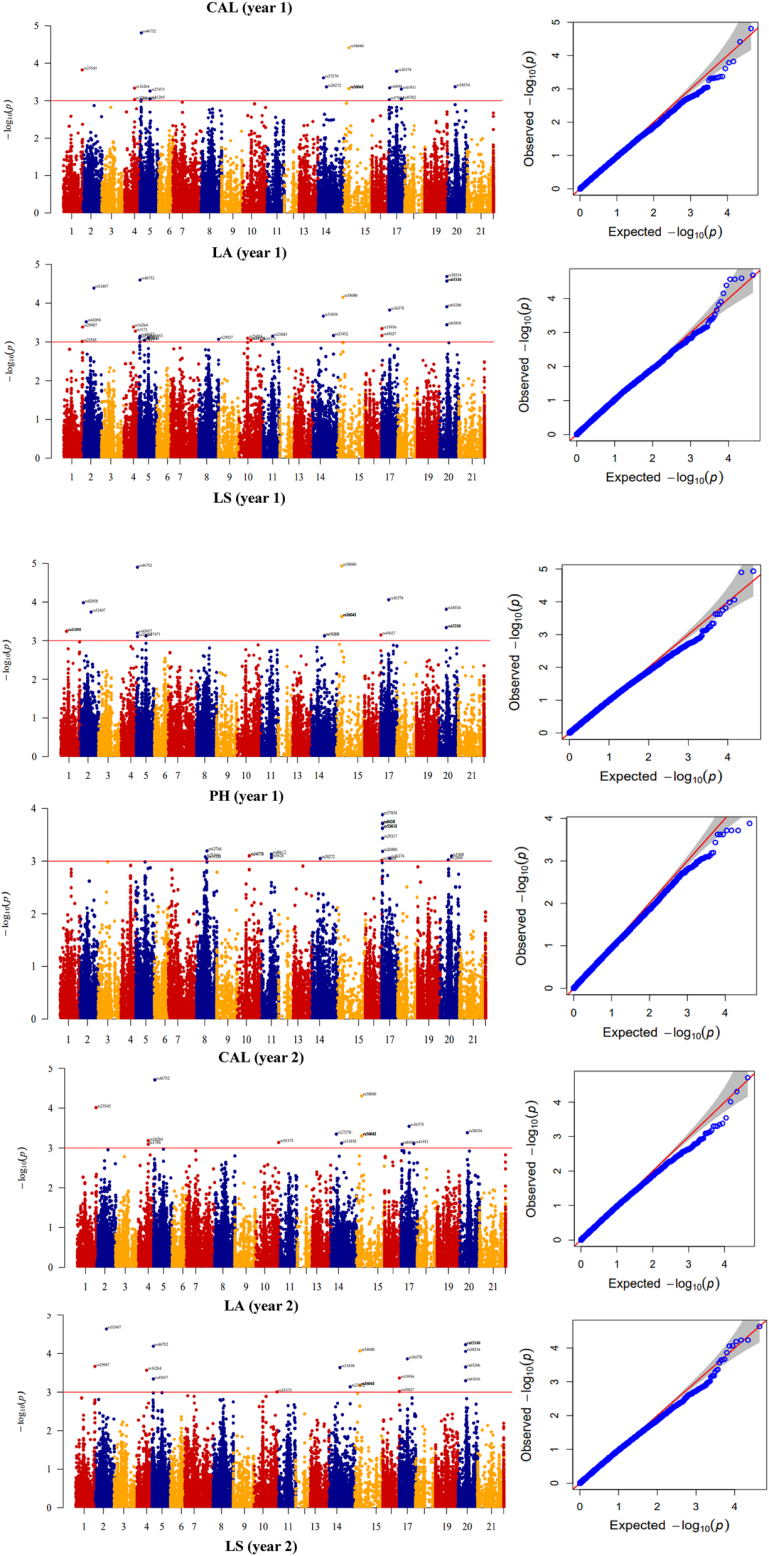

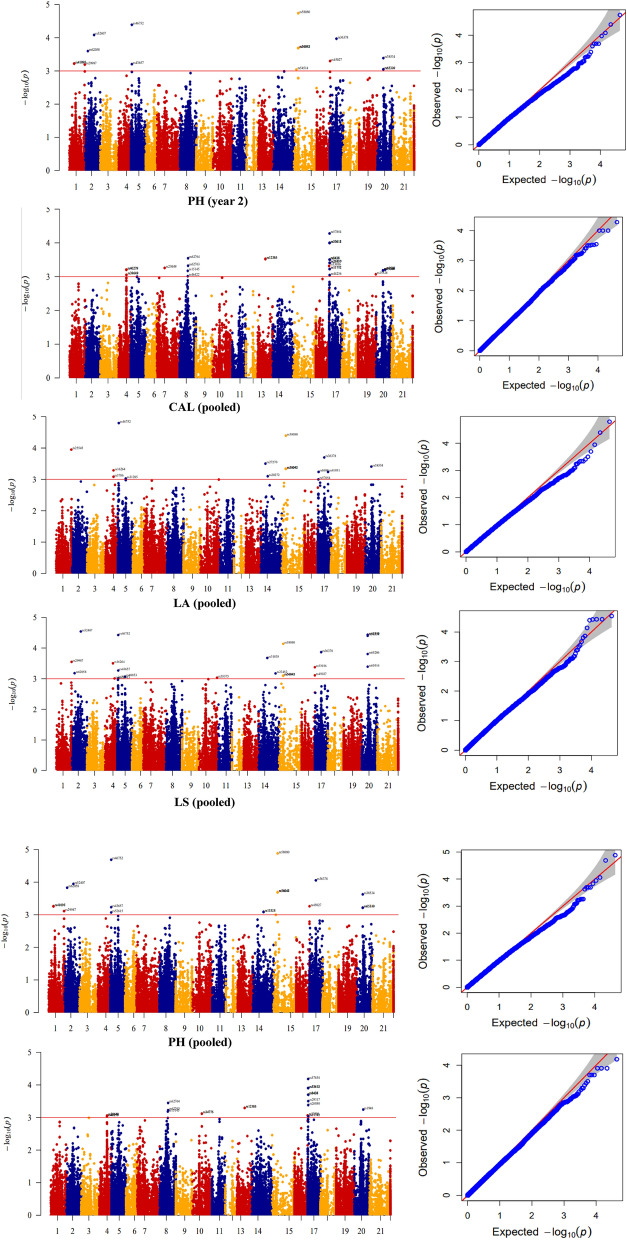
Manhattan and QQ-plots of highly associated haplotypes in Iranian wheat landraces and cultivars. X axis represents wheat chromosomes: (1) 1A, (2) 1B, (3) 1D, (4) 2A, (5) 2B, (6) 2D, (7) 3A, (8) 3B, (9) 3D, (10) 4A, (11) 4B, (12) 4D, (13) 5A, (14) 5B, (15) 5D, (16) 6A, (17) 6B, (18) 6D, (19) 7A, (20) 7B, (21) 7D. *LA* lodged area, *CAI* crop angle of inclination, *LS* lodging score index, *PH* plant height.

Compressed mixed linear model (CMLM) method led to the identification of 465, 497 and 478 significant marker pairs for lodging-related traits (– log10 P > 3), respectively, in the year 1, year 2, and pooled (Fig. 4). There were 150, 260 and 55 MTAs in the year 1, 162, 264 and 71 MTAs in the year 2, and 154, 261 and 63 MTAs in the pooled assigned to A, B, and D genomes, respectively. The largest number of significant marker pairs was found in the B genome followed by the A genome in year 1, year 2, and pooled. The number of significant markers for LA, CAL, LS, PH, NFN, PL, PeL, IL2, IL1, PD, PeD, LD2, LD1, DTH, DTF, DTM, SW, SA, and GY traits were 30, 19, 19, 21, 32, 9, 11, 31, 20, 48, 24, 11, 6, 40, 39, 40, 15, 30, and 20, respectively, in the year 1 (Supplementary 2 and Fig. 4A). The number of significant markers for LA, CAL, LS, PH, NFN, PL, PeL, IL2, IL1, PD, PeD, LD2, LD1, DTH, DTF, DTM, SW, SA, and GY traits were 20, 15, 17, 30, 32, 21, 15, 24, 41, 41, 24, 14, 5, 47, 56, 30, 18, 27, and 20, respectively, in the year 2 (Supplementary 2 and Fig. 4B). The number of significant markers for LA, CAL, LS, PH, NFN, PL, PeL, IL2, IL1, PD, PeD, LD2, LD1, DTH, DTF, DTM, SW, SA, and GY traits were 24, 15, 19, 24, 39, 13, 12, 30, 26, 52, 24, 12, 4, 44, 39, 33, 17, 28, and 23, respectively in the pooled (Supplementary 2 and Fig. 4C). The highest number of significant marker pairs was related to PD (52 significant markers) and the least number was associated with LD1 (4 significant markers) in the pooled. In contrast to other genomes, genome B had a greater effect on lodging, which had the most significant markers for lodging (Fig. 4). Manhattan and Q-Q plots for the lodging-related traits of interest are presented in Fig. 5 and Supplementary Fig. S3.

### Putative candidate genes for lodging tolerance

In-depth analysis was conducted on the markers with the highest significance (P < 0.0001) and pleiotropy. Gene ontologies based on 45 reliable MTAs indicate that candidate gene harboring these SNPs encode proteins that play a variety of roles in various biological processes, such as defense response, calcium ion transmembrane transport, carbohydrate metabolic process, chloroplast organization, nitrogen compound metabolic process, biosynthetic process, protein neddylation, protein phosphorylation, lipid metabolic process, phosphorelay signal transduction system and response to oxidative stress under lodging stress (Table 1). A total of 45 highly significant, functional MTAs were considered "reliable" MTAs for lodging-related characteristics. In choosing reliable MTAs, a high significance threshold and the molecular function were taken into account. The "major" MTAs were selected from the reliable MTAs that that had R^2^ > 10%. A total of 20 major MTAs were detected for lodging-related in two environments (Supplementary Table S5). The following pathways have been discovered based on the rice reference genome; starch and sucrose metabolism (Supplementary Fig. S4), zeatin biosynthesis (Supplementary Fig. S5), amino sugar and nucleotide sugar metabolism (Supplementary Fig. S6), and carbon metabolism (Supplementary Fig. S7).

**Table 1:** Description of expected MTAs using imputed SNPs for lodging traits in Iranian wheat accessions.

No.	SNP	Sequence	Trait- Index	Chromosome	Position (bp)	P-Value	Transcript ID	R^2^	Molecular process	Biological process
1	rs10133	TGCAGATTCTAGTGCGCGCACCGCAAACCCAAGACGGCTGCTGTCCTATCACTACTCAGACGAG	NFN	8	51,209	0.00031	TraesCS3B02G264100	0.167	–	Chloroplast organization
2	rs10253	TGCAGATTGCGCACAGGCTATATATTGATTCATTGAATTTTCTTCTTCTTCTTCTTTATTTGTC	PeD	19	10,479	0.00087	TraesCS7A02G069700	0.134	Hydrolase activity, hydrolyzing O-glycosyl compounds	Carbohydrate metabolic process
3	rs11220	TGCAGCAACACACCAAATAGATCATAGCCAGCTTGCTTGCACTACACGACCTAGCCCGAGATCG	IL1	17	92,187	0.00055	TraesCS6B02G448800	0.191	Monooxygenase activity, iron ion binding, oxidoreductase activity, acting on paired donors, with incorporation or reduction of molecular oxygen, heme binding	–
4	rs13078	TGCAGCAATGACTCATATCAGCAGAAAACAATGATCAAGTTAGCCATGTACTACATGCAATGTG	IL2	14	70,681	0.00073	TraesCS5B02G367400	0.142	Serine-type endopeptidase activity	Proteolysis
5	rs15145	TGCAGCACGGCAAGGTTCACATCGAAACAACGAAGCAACTGAAGAAAGCTACAGGAGAGGAGAG	NFN, PH	8	60,303	0.00064	TraesCS3B02G447100	0.163	Catalytic activity	Nitrogen compound metabolic process, regulation of gene expression, macromolecule metabolic process, primary metabolic process
6	rs16627	TGCAGCAGCATGCGGCGGCTGAGCTCGGGGTCCATGGACACCACCGTCGGGCAGCCCAAGATGT	DTF	5	6832	0.0005	TraesCS2B02G004700	0.133	Monooxygenase activity, iron ion binding, oxidoreductase activity, acting on paired donors, with incorporation or reduction of molecular oxygen, heme binding	–
7	rs17637	TGCAGCAGGAACCAATAACTGATGGTTTCTATTGACGGAAATTGGAAGAAGTTCAGAAATAATA	DTH, DTM	6	73,660	0.00052	TraesCS2D02G477100	0.133	Methylmalonate-semialdehyde dehydrogenase (acylating) activity, copper ion binding, oxidoreductase activity	Response to oxidative stress
8	rs19969	TGCAGCATGGGTCCCTCTCTGGCCAACCACTCTTGGTCAGCTATGAACACCGCTCCATATCCCA	DTM	6	79,343	0.00064	TraesCS2D02G508900	0.131	Protein kinase activity, calcium ion binding, polysaccharide binding	Protein phosphorylation, cell surface receptor signaling pathway
9	rs19970	TGCAGCATGGGTCCCTCTCTGGCCAACCACTCTTGGTCAGCTATGAACACCGCTCCATATCCCA	DTH	6	79,343	0.0004	TraesCS2D02G508900	0.135	Protein kinase activity, calcium ion binding, polysaccharide binding	Protein phosphorylation, cell surface receptor signaling pathway
10	rs22878	TGCAGCCCACAATCTTCCCGACTTACAACGTATGTGATGTGTGAAGAACAGATTACTAATAAGC	PL	7	23,905	0.00078	TraesCS3A02G046500	0.056	Hydrolase activity	Lipid metabolic process, lipid catabolic process
11	rs26880	TGCAGCCTCGCGTTCTCGCGGACGGTCAGAACCCGAGATCGGAAGAGCGGGATCACCGACTGCC	PH	17	4546	0.00039	TraesCS6B02G022800	0.207	Chromatin binding, DNA (cytosine-5-)-methyltransferase activity, methyltransferase activity	
12	rs29317	TGCAGCGATTCATTCGACTTGGCGAGCAAAAACGGGGCCTTAGGCAGAGCAATGCTCACCTCGA	PH	17	4546	0.00039	TraesCS6B02G023300	0.207	Double-stranded DNA binding	Regulation of DNA-templated transcription
13	rs29320	TGCAGCGATTCCTTACTCCTCACTAGCAACGTTGGAGCGTAAGAAAAAGCAATGCCCACCTCGG	DTM	6	77,069	0.00055	TraesCS2D02G488800	0.132	Double-stranded DNA binding	DNA-templated transcription termination
14	rs2942	TGCAGAATTCATTAAGTTCCCCTTGTTCAAATGCTACAAGTCGATGCTGCTTCTCCTTGTTCAT	PL	17	62,609	0.00012	TraesCS6B02G391200	0.069	Nucleic acid binding, ATP binding	–
15	rs29601	TGCAGCGCAGCAGTAGAAAGATGAGTATATTTCTTGTCGCAGCCCTGGTGGCCTGCCTGGTCAG	PD	15	97,912	0.00088	TraesCS5B02G391900	0.072	Protein kinase activity, ATP binding	Protein phosphorylation
16	rs30872	TGCAGCGCGTAGCCTGACTGAGCAGCGAGAGACAAGCAGCAAGGTCAGCAACCATGGGGGGAGG	DTF	8	39,841	0.00084	TraesCS3B02G069100	0.13	Transmembrane transporter activity	Oligopeptide transport, transmembrane transport
17	rs31183	TGCAGCGCTGCATGGAGCCACGCTGGGCGACAGCGTGTCCAAGGAGGAGGCGGCCGCGGTCCTC	PeL	20	109,456	0.00066	TraesCS7B02G433100	0.093	Calcium ion binding	–
18	rs31186	TGCAGCGCTGCATGGAGGCCACGCTGGGCGACAGCGTGTCCAAGGAGGAGGCGGCCGCGGTCCT	PeL	20	109,456	0.00057	TraesCS7B02G433100	0.093	Calcium ion binding	–
19	rs33741	TGCAGCGTGCCTGTGGCTATACGTACTGATCGTTTCCCCGTGTTCCTCCACACGGGCAGGTTCG	IL1	4	59,228	0.0005	TraesCS2A02G170200	0.192	Strictosidine synthase activity	Biosynthetic process
20	rs36483	TGCAGCTCCCAGCCCAAAGGGGGAGCTGCTTATTTGGGCGCCTCTGCTCAAGCACCGAGATCGG	ID2	17	94,461	0.00006	TraesCS6B02G453300	0.12	Protein binding	–
21	rs38534	TGCAGCTGCAACAACCGCTCGAGCAAAAGCTAGCAGAGAGAGAAAAGAAGGAACCGTGCATGGA	CAL, LA, LS	20	45,510	0.00042	TraesCS7B02G043400	0.292	Oxidoreductase activity	–
22	rs39281	TGCAGCTGCTGGAAGGACGGGTGGTGGTGGTAGATGGGGACCCCTCTTATAGGCTTGAGCAAGG	SW, NFN	17	1137	0.0009	raesCS6B02G012800	0.052	Transcription cis-regulatory region binding, DNA binding	Regulation of DNA-templated transcription, abaxial cell fate specification
23	rs44017	TGCAGGAGAGGGTTACCGTGCCAAGGACGGCGTGGAAGCTAGCCGACATCTTCATCCTCTGCCT	GY	8	21,628	0.00018	TraesCS3B02G034600	0.13	Transferase activity, glycosyltransferase activity, cellulose synthase (UDP-forming) activity, mannan synthase activity	Plant-type primary cell wall biogenesis, cellulose biosynthetic process, cell wall organization, plant-type cell wall organization or biogenesis
24	rs44236	TGCAGGAGCAGGGTGGGAGGGATATGGTGGGGCATAAGAGGGTGTGGATGAGAGGATGAGCTGC	DTF, PH	17	4546	0.00038	TraesCS6B02G023900	0.134	Nucleotide binding, ATP binding, transferase activity, NEDD8 transferase activity	Protein neddylation
25	rs46422	TGCAGGCAGGTGAACGACTGTACAGTCAAGCCATGGATATAATCAGGCACTCGCACGACATCGT	PH	8	60,303	0.00097	TraesCS3B02G444800	0.202	Protein binding	–
26	rs47117	TGCAGGCCCGCAACAGCTGCCATGAACCATTCTATAACACCTCAATCAAGCTCAACCGTCCGTT	PD	9	5684	0.00058	TraesCSU02G234600	0.075	Polysaccharide binding	–
27	rs4727	TGCAGACTACGACGCCGCCCCAGCTTCGCTCCAGCGGCCCACGACTCTGTTCTGCATTCCCGAG	DTF	5	0	0.0005	TraesCS2B02G000300	0.133	Magnesium ion binding, terpene synthase activity, lyase activity	Diterpenoid biosynthetic process
28	rs4727	TGCAGACTACGACGCCGCCCCAGCTTCGCTCCAGCGGCCCACGACTCTGTTCTGCATTCCCGAG	DTH	5	0	0.00085	TraesCS2B02G000300	0.131	Magnesium ion binding, terpene synthase activity, lyase activity	Diterpenoid biosynthetic process
29	rs50030	TGCAGGGATTTCATTGTCGTCACCTTCTTGGTGTTGTTGGCAGGAAGCTTAAATGCCTTCCGTG	IL2	19	133,352	0.00029	TraesCS2D02G042200	0.148	ADP binding	Defense response
30	rs50030	TGCAGGGATTTCATTGTCGTCACCTTCTTGGTGTTGTTGGCAGGAAGCTTAAATGCCTTCCGTG	IL1	19	133,352	0.00028	TraesCS5B02G401100	0.195	ADP binding	Defense response
31	rs51159	TGCAGGGGCTCAACCTCGCCCATACGACATGGCAAAGTTTGACACGGCACGACTACCGTGAGGT	DTH, DTF	21	157,445	0.00034	TraesCS7D02G545800	0.136	"Protein kinase activity, protein binding,	Protein phosphorylation
32	rs52123	TGCAGGGTTTTGAGAGAAGAACTGATCCCACCTTTCAGGGAAGACCGAGATCGGAAGAGCGGGA	ID2, PeL	1	114,237	0.00099	TraesCS1A02G431100	0.102	Protein binding	–
33	rs52244	TGCAGGTACAATGTACGGCAGCCAAAATTGCGCGTTGACATGATTCTCACCCTCAGGTCGGTCG	PeD	20	109,456	0.00066	TraesCS7B02G434600	0.136	Protein binding	–
34	rs52407	TGCAGGTAGATGAAACGGTGGACGTGCGGATGGTGGGACGAGACGGGGCCGCCGTGCGTGTGAC	LA, LS	2	66,042	0.00002	TraesCS1B02G387900	0.278	DNA-binding transcription factor activity, protein dimerization activity	Regulation of DNA-templated transcription
35	rs54416	TGCAGGTGGGGGCGCGCCTGCAAAACGGCGAAATTAGACTCCCCAGGTGCCCCTCGGGTTCCTG	PeL	7	135,636	0.00026	TraesCS3A02G494700	0.098	D-arabinono-1,4-lactone oxidase activity, flavin adenine dinucleotide binding, FAD binding	–
36	rs5683	TGCAGAGATGCGAGTAGCTTTTTTTGAAAGGGAGATGCGAGTAGCTGAAGTCTGAAGAAACGCA	NFN	8	51,209	0.00071	TraesCS3B02G264100	0.162	–	Chloroplast organization
37	rs57440	TGCAGTATGATGCTCATTTCGATCTGTGACTGGCCGAGATCGGAAGAGCGGGATCACCGACTGC	PD	2	45,574	0.00085	TraesCS1B02G211200	0.073	Catalytic activity, choline-phosphate cytidylyltransferase activity	CDP-choline pathway, biosynthetic process
38	rs58173	TGCAGTCCACGCTCCCAGACAGCGTGGACTGGAGGGCCCGAGATCGGAAGAGCGGGATCACCGA	NFN	6	22,050	0.00021	TraesCS2D02G068500	0.169	Cysteine-type peptidase activity	Proteolysis
39	rs60228	TGCAGTGATCCCTGAAGGTAAATCCGATGCCGCAGCAAATGCGAGGCACCCAACGGAGAAGATA	PL	7	22,198	0.00039	TraesCS3A02G047100	0.061	Aspartic-type endopeptidase activity	Proteolysis
40	rs63000	TGCAGTTCAGTCGGTACCTCAAGCTGTATGTGCTGCTGAAACGGGACCGAGTTCCCCTTGGTGC	DTF, DTM	8	41,546	0.00017	TraesCS6A02G211000	0.139	Actin filament binding	Arp2/3 complex-mediated actin nucleation
41	rs65345	TGCAGTTTTCCAGTCGCACGTCCTCCAGCGAGGGGCACCTGTCGTCACTGATGTGGCTCGCAAA	DTF	10	83,789	0.00065	TraesCS4A02G325200	0.131	Protein binding	
42	rs7047	TGCAGAGTACAACATACACACAGTTAAAATGCAAGGCTTGTTCGACAAATAGCCCTTTCTAAGG	DTM	6	78,206	0.00032	TraesCS2D02G497800	0.135	–	Regulation of cellular response to heat
43	rs8436	TGCAGATCCTAACACGGCACGCGTCCCAGAACCGTCTTCCCCGTCTAACGCGCCCGACCGACTC	PH	17	4546	0.00031	TraesCS6B02G023900	0.208	Nucleotide binding, ATP binding, transferase activity, NEDD8 transferase activity	Protein neddylation
44	rs8943	TGCAGATGAAGACACACTCTTAGCAGGCCAATCGCTCACCGCTGGCGACAAGCTCGTCTCGAGA	IL2	14	70,681	0.00096	TraesCS5B02G367800	0.141	–	Recognition of pollen
45	rs960	TGCAGAAATGGGGGGCAGAAAATCGCACCCAGAAAACCCCACCAAAACCCTCGCCCTGTTGACC	PD	7	90,077	0.0005	TraesCS3A02G441200	0.076	Protein binding, zinc ion binding, histone-lysine N-methyltransferase activity	Histone methylation, histone lysine methylation

*LA* lodged area, *CAI* crop angle of inclination, *LS* lodging score index, *PH* plant height, *NFN* number of nodes, *PL* peduncle length, *PeL* penultimate length, *IL1* internode length 1, *IL2* internode length 2, *PD* peduncle diameter, *PeD* penultimate diameter, *ID1* internode diameter 1, *ID2* internode diameter 2, *DTH* days to heading, *DTF* days to flowering, *DTM* days to maturity, *SW* spike weight, *SA* spike area, *GY* grain yield.

Wheat chromosomes: (1) 1A, (2) 1B, (3) 1D, (4) 2A, (5) 2B, (6) 2D, (7) 3A, (8) 3B, (9) 3D, (10) 4A, (11) 4B, (12) 4D, (13) 5A, (14) 5B, (15) 5D, (16) 6A, (17) 6B, (18) 6D, (19) 7A, (20) 7B, (21) 7D.

### Genomic prediction for lodging-related traits

**Figure 6:**
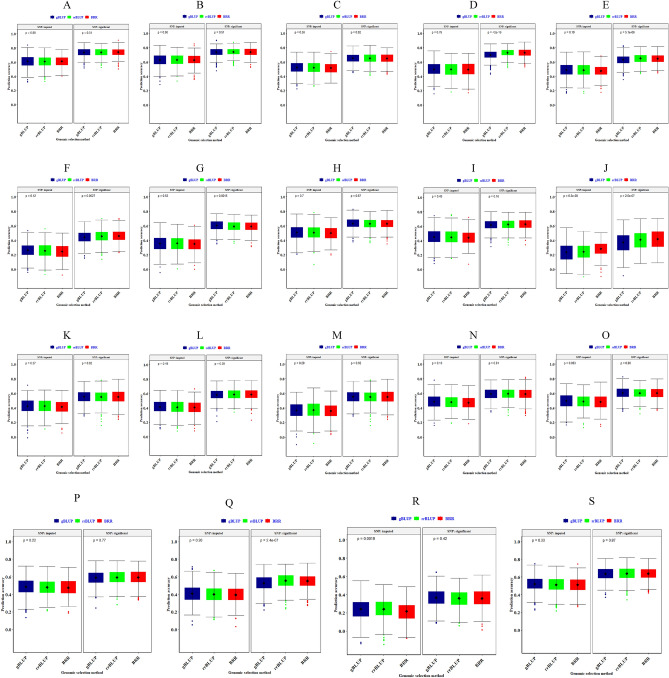
The efficacy of genomic selection (GS) on genomic prediction (GP) accuracy for 19 lodging traits in Iranian landraces and cultivars. (**A**–**S**) The accuracy of GP via ridge regression-best linear unbiased prediction (RR-BLUP), genomic best linear unbiased predictions (GBLUP), and Bayesian ridge regression (BRR) genomic selection (GS) methods are demonstrated with green, red, and blue colors, respectively. The boxplots show the first, second (median) and third quartiles. The middle points indicate a mean of GP accuracies for the trait of interest. Lodged area (**A**), Crop angle of inclination (**B**), Lodging score index (**C**), Plant height (**D**), Number of nodes (**E**), Peduncle length (**F**), Penultimate length (**G**), Internode length 1 (**H**), Internode length 2 (**I**), Peduncle diameter (**J**), Penultimate diameter (**K**), Internode diameter 1 (**L**), Internode diameter 2 (**M**), Days to heading (**N**), Days to flowering (**O**), Days to maturity (**P**), Spike weight (**Q**), Spike area (**R**) and Grain Yield (**S**).

The BRR, GBLUP, and RR-BLUP approaches using whole SNPs led to the identification of the highest prediction accuracies for 1, 13, and 5 phenotypes, respectively (Fig. 6). The highest prediction accuracy was determined via the GBLUP model for DTF, DTH, DTM, GY, ID1, IL1, IL2, LA, LS, NFN, PH, PL, SA, via the RR-BLUP for RR-BLUP for CAL, ID2, PeD, PeL, SW, and via the BRR for PD traits. The best prediction accuracy was related to the three traits CAL, LA, and LS, respectively with an average of 0.627, 0.610, and 0.519, while the lowest prediction accuracy was related to trait SW with an average of 0.233 (Fig. 6).

GBLUP, RR-BLUP, and BRR approaches using significant SNPs displayed the highest prediction accuracies for the phenotypes 6, 6, and 7, respectively (Fig. 6). The highest prediction accuracy by the GBLUP was obtained for Convexity; by the RR-BLUP for DTH, IL1, ID2, PeD, PeL, SW; as well as by the BRR for PD, DTM, IL2, LA, PH, and PL traits. The best prediction was related to the three traits CAL, LA, and LS, respectively with a mean of 0.737, 0.637, and 0.650, while the lowest accuracy was related to trait SW with a mean of 0.361 (Fig. 6).

## Discussion

It appears that Iranian wheat accessions are more diverse than the cultivars, and thus have a higher lodging score index due to the higher DTH, DTF, DTM, IL, and PH, and lower stem diameter and NFN. The stem diameter explains 55% of the variance in the lodging index^45^, so it is viewed as an important parameter for enhancing lodging resistance due to the increased amount of lignin, cellulose, and carbohydrates soluble in water. Increasing internode diameters can lower tiller numbers per unit area and therefore grain yield^46^, thus the association between grain yield and stem structure needs to be further explored. According to the correlation between phenological traits and lodging, an increase in DTH, DTF, and DTM leads to further growth, which leads to lodging^47^. Trait correlations revealed lodging to be directly related to plant high and other stem traits^48^. The properties of stems and their composition contribute significantly to stem bending resistance in crops^13,49^. The results show the lodging index correlates more strongly with ID1 than ID2, suggesting that the first internode contributes more to lodging resistance in wheat. Considering the first internode's nearly twice the material strength of the second internode, this association makes sense^50^. A weakness affecting stem strength, root characteristics, or soil structure can contribute to anchorage failure and lodging susceptibility^51^. The research by Berry et al.^52^ found a decrease in lodging risk as a result of an increase in stem strength and root anchorage. Similarly, Tripathi et al.^45^ found lodging resistance negatively related to spike area and weight. Thus, the increase in stem diameter of wheat genotypes can reduce lodging risk, making these genotypes ideal for breeding programs aimed at improving lodging resistance.

If population structure is not appropriately accounted for in mapping studies, there is the possibility of false associations^53^. There are two types of kinships that cause false positives in GWAS: ancestry differences and cryptic relatedness. Cryptic relatedness occurs when some accessions of a plant are closely related; however, their shared ancestry is not disclosed to the breeder^54^. PCA analysis and clustering were conducted on Iranian wheat genotypes to evaluate their population structure. The panel of accessions was stratified into three groups based on the results. Such a genetic separation can be explained by selection effects in breeding programs^55^. The same grouping was observed on these Iranian wheat genotypes by Rabieyan et al.^38^. Cultivars made up one group, while landraces made up the other two groups, regardless of their geographic origins. There was no correlation between the clustering of wheat accessions and the origin or geographical distribution of these accessions. In part, this can be explained by migrations of farmers to different regions and germplasm exchanges among researchers and institutes worldwide^22^.

According to the results, the detected SNPs covered the wheat genome well. A higher number of SNPs were found in genome B compared to genome D. Therefore, chromosome size is directly related to SNP density^56^. Due to evolutionary processes, genome B contains a greater number of SNPs^57,58^.

From our observations, Genomes B, A, and D have the lowest LD, respectively. Breeding efforts during the period of lodging-related traits have presumably resulted in cultivars exhibiting higher LD than landraces, particularly in genome D^59^. Due to selection events during crop breeding, cultivars exhibit higher LD compared to landraces^38^. In addition to evolutionary processes, breeding schedules are likely to have influenced the differences in LD between genomes and accessions^60^. Liu et al.^61^ also noticed that LD decay distances were significantly less in native populations than in varieties of wheat in China/Pakistan. A number of factors besides selection breeding affect linkage disequilibrium in wheat and other plants, including population relatedness, genetic drift, mutation, recombination, and mating systems^38^.

Numerous efforts have been made so far to locate QTLs and genes affecting wheat traits in order to facilitate marker-assisted breeding for wheat^62,63^. In this study, MTAs were also detected, adding to the previous list of candidate genes and markers. In spite of this, aligning our results with earlier studies is challenging due to different reference genome models than IWGSC Ref.Seq, inaccurate genomic locations, or combining different markers (GBS-derived SNP versus SSR and DART)^62–65^. Of course, detecting MTAs on the same chromosome as previous projects increase their assurance. A total of four MTAs were revealed on each of chr 1A, 2B, 6B, and 2D in regards to plant height. There are genes on all 21 chromosomes that are involved in controlling plant height in wheat^29–31^. The reduced height (*Rht*) genes (*Rht1–Rht24*) have been classified in wheat^32,33^, where *Rht8* on chromosome arm 2DS has been extensively studied^34,35^. A total of two QTLs were detected on chromosome 2DL, while the ones reported by Börner et al.^36^ on chromosome 2DS were not detected. There is a similar pattern of QTLs on chromosomes 1A, 2B, 6B, and 2D described by Griffiths et al.^66^. Plant height is increased by 2–7 cm by the major height QTLs found in this study, and when all major height QTLs are taken into account, 26 cm is gained in height. This discovery may help to explain why Iran breeders select tall plants within semi-dwarf backgrounds. There may, however, be a way to balance QTLs bringing yield and height increases by using height QTLs without effect on yield. As a result of the project, QTLs were identified for height, lodging score index, internode length, internode diameter, grain yield, flowering date, and spike weight. Five MTAs for grain yield were recorded on the chr 4A, 5A, 3B, 5B, and 3D in this study. Earlier research efforts have discovered MTAs/QTLs for grain yield on wheat chr 7B^67–69^, 7A^67,69–71^, 5B^67,69,72^, 3D^69^, 3A^67,69,73,74^, 2B^69,73–76^, and 1B^69,74,75^. Individual QTL for lodging score index, internode length, and internode diameter were estimated by the model of lodging. A number of MTAs for the above traits were found on the 1A, 2A, 3A, 6A, 7A, 1B, 3B, 5B, 6B, 1D, 2D, and 2D. Berry and Berry^20^, identified QTLs on chromosomes 1A, 3A, 6A, 5B, 6B, 2D, and 7D for lodging-related traits such as stem diameter, internode length, and lodging score index. Singh et al.^23^ explored lodging tolerance via GWAS and identified a key genomic region on chromosome 2A.

The flanking sequences of imputed SNPs were aligned versus the RefSeq version 2.0 findings in the recently published study^77^. It was interesting to observe that most of the marker pairs were located in the protein-coding regions of the genome, which are responsible for controlling transcription, defense response, oxidation–reduction process, calcium ion transmembrane transport, carbohydrate metabolic process, and sulfate transport that are likely interconnected in lodging resistance. Researchers have found similar results in earlier studies^20–22^. The following pathways have been identified based on the rice reference genome: starch and sucrose metabolism, zeatin biosynthesis, amino sugar and nucleotide sugar metabolism, and carbon metabolism. Based on various studies, carbon metabolism, and starch and sucrose are effective on the lodging resistance in plants^78–80^. There are two types of carbon assimilated structural carbohydrates and non-structural carbohydrates (NSCs). The former (such as lignin and cellulose) contribute mostly to the cell composition and densities of the stem lodging resistance, while the latter (such as starch and sucrose) contribute to metabolism and yield^78^. Studies on carbon assimilate metabolism in gramineous crops, especially NSCs, have been conducted in recent years. In these studies, NSCs were found to vary significantly by sowing method and planting density and were closely associated with yield and lodging^78,79^. A stem sheath’s NSCs are the key to stem lodging resistance, and they can continue providing assimilates while photosynthetic capacity is diminished during grain-filling, thereby reducing yield loss caused by stress^80,81^. Mizuno et al.^82^ stated that the metabolism of sugar has an effect on the amount of lodging resistance so that lodging decreased the amount of sucrose, starch, and the ratio of sucrose to total sugars in sorghum stems. The hormone zeatin (Z) significantly increases the growth of buds^83^. According to the studies, the application of Z significantly influenced the growth regulation of tillers and tiller’s buds. Therefore, Z application will help plants anchor well to the soil, thereby creating a more lodging-tolerant environment^83,84^.

Our findings suggested that genomic predictions are useful in predicting wheat genotype's performance, allowing phenotyping to be limited to a fraction of germplasm instead of the entire collection^85–87^. Furthermore, Kehel et al.^88^ reported that genomic selection can accurately predict key traits within wheat accessions, especially for traits with moderate to high heritability. A prediction model usually accounts for stratified populations by using the first five principal components as covariates^87–90^. In the Iranian wheat genotypes, there is a significant population structure with 30.5% of diversity coming from the first five eigenvalues. It was also observed in other studies that the population structure negatively affected GWAS and GP models^90^. Based on our observations, the GBLUP model provided the highest prediction accuracy. A study by Shabannejad et al.^37^ investigated classic strategies to exploit GP accuracy in wheat cultivars and landraces under normal and drought conditions. Using the GBLUP and BRR methods, they identified the highest GP accuracy. Singh et al.^23^ examined genome prediction models for lodging‑related traits and found high predictive accuracy (0.42) across populations and environments. The authors observed that obtaining the highest GP accuracy depends on the genetic variation, the genetic architecture of the trait, the level of LD, and the genomic selection approach. As a result, the GBLUP model can detect genetic impacts in wheat populations better than other genomic prediction models.

## Conclusion

Using validated lodging measurements along with association and genomic prediction analyses, we provide evidence in support of a polygenic genetic architecture of lodging in wheat. Our findings have diverse applications in plant breeding and genetics. The results of our research provide new insight into the molecular mechanisms underlying lodging resistance traits in wheat. To develop lodging resistance wheat cultivars, marker-assisted selection can target genes controlling these traits, including LS, PH, NFN, and IL1. Moreover, genomic selection by using our putative genetic markers along with GBLUP-based genomic prediction will help to achieve the above-mentioned goal.

## Methods

### Plant material and phenotyping

**Figure 7:**
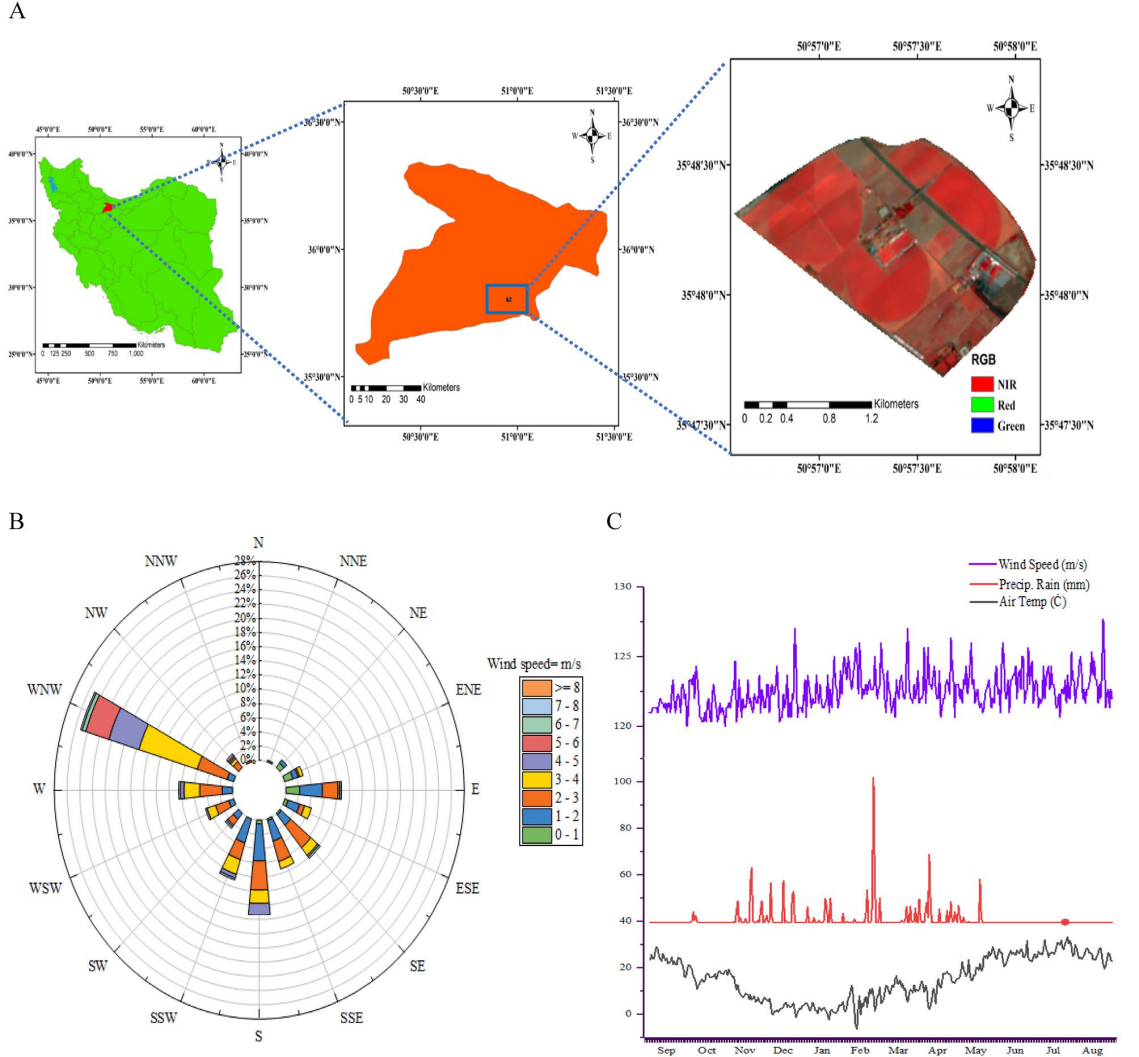
The geographical location of the study area (**A**), and average two-year wind rose plot (**B**) and climatic parameters (**C**).

The research was conducted in the Alborz province in the Department of Agriculture & Natural Resources Campus (35°48′59ʺN, 51°58′48ʺE, 1321 m elevation) (Fig. 7a). We conducted an alpha-lattice experiment with two replications on 298 wheat accessions (208 native landraces and 90 cultivars) to analyze lodging and related traits in wheat under normal conditions (Supplementary Table S6). The replications each contained 30 incomplete blocks containing 10 genotypes each. Wheat accessions were grown in plots with a plant density of 250 plants/m^2^ in four rows (1 m × 2 m) at 0.20 m intervals. The wind rose plot, and the climatic characteristics are presented in Fig. 7B,C, and Supplementary Table S7. Based on evaporation from an evaporation pan, a 40 mm threshold was determined for irrigation to be implemented in irrigated crops. The lodging and related traits of wheat accessions were measured in the pre-physiological stage. The traits measured in this study were as follows: Lodged area (LA, %), Lodging score index (LS), Crop angle of inclination (CAI, degree), Plant height (PH, cm), Internode length 1 and 2 (IL1 and IL2, cm), Penultimate length (Pel, cm), Peduncle length (PL, cm), Internode diameter 1 and 2 (ID1 and ID2, mm), Penultimate diameter (PeD, mm), Peduncle diameter (PD, mm), Number of node (NFN, n), Grain Yield (GY, g per plant), Spike weight (SW, g), Spike area (SA, cm^2^), Days to maturity (DTM), Days to flowering (DTF), and Days to heading (DTH).

The authors declare that all study complies with relevant institutional, national, and international guidelines and legislation for plant ethics in the “Methods” section. Samples are provided from the Gene Bank of Agronomy and Plant Breeding Group and these samples are available at USDA with USDA PI number (Supplementary Table 2), respectively. The authors declare that all that permissions or licenses were obtained to collect the wheat plant.

### LA, CAI, LS

**Figure 8:**
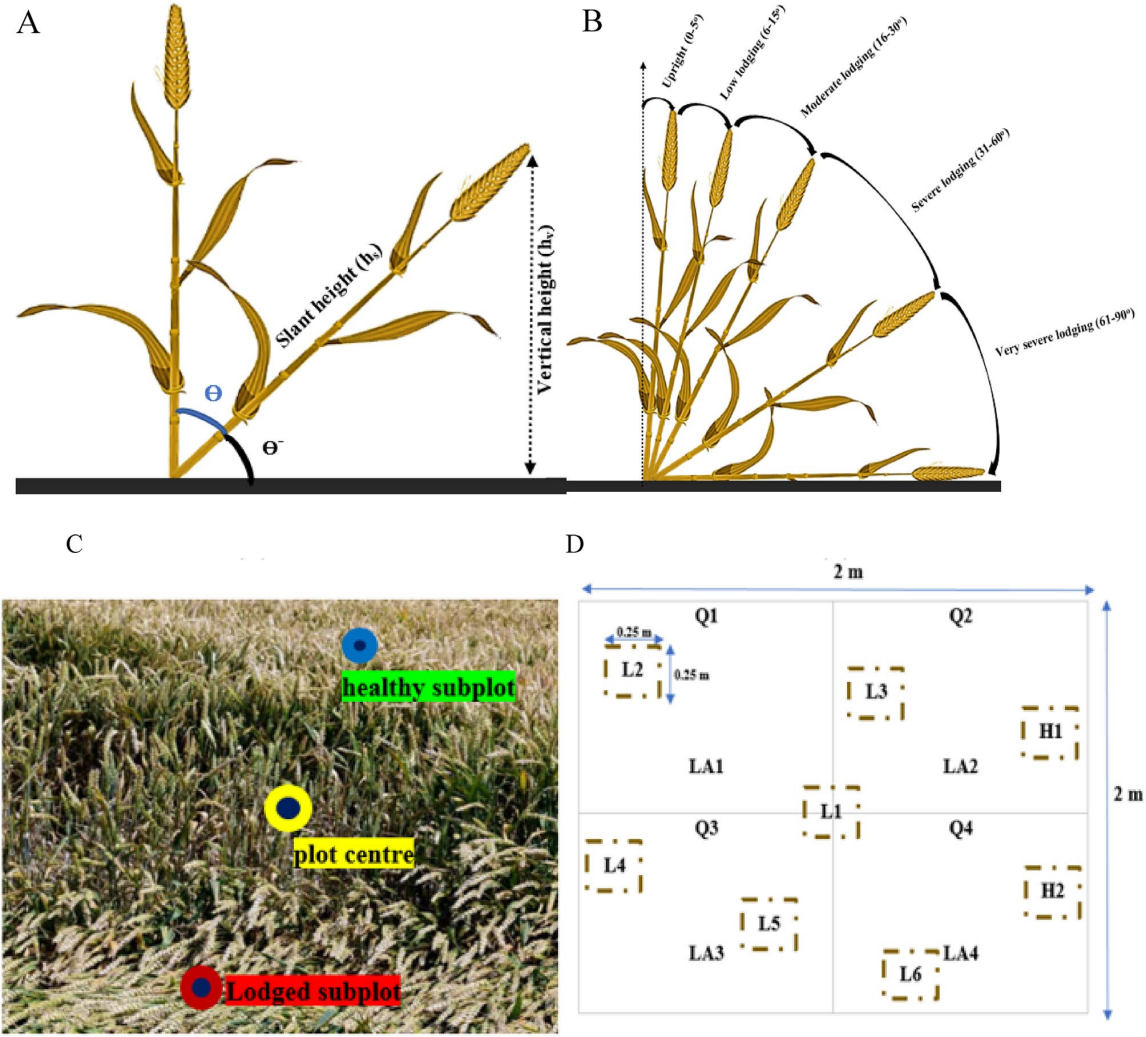
Measurement of crop angle of inclination (**A**) and presentation of various lodging stages (**B**). Presentation of the plot center and the healthy/lodged subplots in the field (**C**). Division of the plot into four quadrants Q1, Q2, Q3, and Q4 (**D**). LA1, LA2, LA3, and LA4 are corresponding to the lodged area in each quadrant. In this scenario, H1 and H2 present the healthy subplots while L1 to L6 are the lodged subplots. The CAI is estimated via averaging the CAI and LA calculated in the six lodged subplots and in each quadrant, respectively.

In order to determine whether the wheat plots were healthy (H) or lodged (L) in the field, the CAI was calculated for each plot using the lodged area (LA [0–100%]) and vertical angle (CAI [0–90°]) (Fig. 8A,B)^91^. The CAI was calculated using trigonometric calculations and a plumb bob. In this case, the string of the plumb bob was suspended from the top of the crop, and it was just possible for the plumb bob's tip to touch the soil, ensuring precise vertical height (hv) measurements. The slant height (hsl) of lodged plants was measured using a plumb bob. The CAI was then calculated from the vertical using the Eq. (1) ^91^:

1 $$\theta degree={90}^{{\text{o}}}-{Sin}^{-1}\frac{{h}_{v}}{{h}_{sl}}$$

lodged area was also analyzed visually using a quadrant methodology. Using this approach, the LA % was computed in each of the four quadrants starting from the center of the plot, and then the final LA for each plot was calculated. The Fig. 8C and D depict lodged and healthy subplots for healthy plots, each trait was measured in three subplots (0.25 m^2^), while for lodged plots, four to eight subplots were measured in order to account for spatial heterogeneity within each lodged patch^91^.

To define lodging and healthy severity classes, a normalized lodging score index (LS [0–1]) was calculated by merging LA and CAI [Eq. (2)]. The plot was labeled as H (LS = 0.0) if no lodging was observed. In the presence of lodging, wheat plots were classed as Upright (0.0 < LS ≤ 0.05), low lodged (LL) (0.06 < LS ≤ 0.15), moderately lodged (ML) (0.16 < LS ≤ 0.30), severely lodged (SL) (0.31 < LS ≤ 0.60), and very severely lodged (VSL) (0.61 < LS ≤ 1.0^91^.

2 $$LS=\frac{LA}{100}*\frac{CAL}{{90}^{{\text{o}}}}$$

Phenotypic [Eq. (3)] and genotypic [Eq. (4)] variances, phenotypic coefficient of variation [PCV, Eq. (5)], genotypic coefficient of variation [GCV, Eq. (6)], and broad sense heritability [H^2^, Eq. (7)] were computed using standard formulas^92^.

3 $${\text{Phenotypic variance }}(\sigma^{{2}} ph) \, = \sigma^{{2}} g \, + \sigma^{{2}} e$$

4 $${\text{Genotypic variance }}(\sigma^{{2}} g) \, = \, \left( {{\text{MSP }} - {\text{ MSe}}} \right) \, /{\text{ r}}$$

5 $${\text{PCV}} = \, \surd \sigma^{{2}} ph/{\overline{\text{X}}} \times { 1}00$$

6 $${\text{GCV}} = \, \surd \sigma^{{2}} {\text{g}}/{\overline{\text{X}}} \times { 1}00$$

7 $${\text{H}}^{{2}} = \, (\sigma^{{2}} {\text{g }}/\sigma^{{2}} {\text{ph}}) \, \times { 1}00$$

where: *MSe*, error mean square; *MSP*, mean square for phenotypes (varieties); *r*, number of replications; *X̅*, was the population mean for a specific trait; σ^2^*ph,* phenotypic variance; *σ*^2^*g*, genotypic variance; *σ*^2^*e*, error variance.

### Statistical analysis

Analysis of variance and the best linear unbiased prediction (BLUP), were estimated using METAR v2.1^93^. The diversity of Iranian wheat accessions was evaluated and compared using advanced statistical analysis. The box plot was drawn with R 4.1 software using ggplot2, dplyr, and ggpubr packages. Also, correlation diagrams were drawn using the R packages corrplot and RcolorBrewer. Cluster analysis and heatmaps were implemented with the R 4.1 packages gplots, dendextend, and d3heatmap in order to classify wheat accession types.

### Genotyping and SNP imputation

By using CTAB, wheat seedling genomic DNA was extracted and RNA contamination was removed with RNase (ribonuclease)^94^. A Thermo Scientific NanoDrop was used to determine DNA concentration, and a 0.8% agarose gel was used to evaluate DNA integrity. Genotyping-by-sequencing (GBS) was employed to genotype all 298 wheat samples^95^. A library of GBS has been developed and sequenced as described by Alipour et al^58^. Each sequencing read was trimmed to 64 bp and grouped into sequence tags. The SNPs were explored using BLAST, which allows for up to 3 bp of mismatch. The UNEAK pipeline in TASSEL^96^ was used to call SNPs. To avoid false-positive SNPs originating from sequencing errors, SNPs with a missing rate < 10% across samples, a minor allele frequency (MAF) > 5% and heterozygosity < 10% were excluded. Missing data were imputed using the LD KNNi method implemented in TASSEL^96^. For the SNP calling, we used the W7984 bread wheat genome as the reference genome^97^.

### Population structure and kinship matrix

Population structure inference was performed using an admixture model implemented in the Structure software. The assumptive number of subpopulations (K) was regarded from K = 1 to K = 10 and 10,000 burn-in steps were followed by 10,000 MCMC steps^98^. The most likely K value was determined using the ΔK method in Structure harvester^99^. The kinship matrix (K) was calculated with the EMMA algorithm using the GAPIT package in R software^100,101^. A principal component analysis (PCA) was also performed using the Tidyverse package in R. To determine the relationship between landraces and cultivars, Archaeopteryx constructed a neighbor-joining tree based on a pairwise distance matrix^101^.

### GWAS analysis

For each trait, BLUPs values of the years were used for genome-wide association studies. The package CMLM in R was used to detect marker-trait associations (MTAs)^38^. We used a significance threshold (cut-off) of − log10 (P-value) > 3.0 (P < 0.001) for identifying significant associations in the model (as reported by many authors)^58,96^. All SNPs which met this cut-off value was categorized as significant MTAs^38^. GWAS results were summarised using Manhattan plots for visualizing associations between genotypes and phenotypes using the GAPIT package^102^. In this plot, the x-axis and y-axis represents the genomic position of SNPs and the − log10 (*P*-value) obtained from the F-test, respectively. A Q-Q plot was also done to assess the distribution of p-values results obtained from the GWAS analyses^68^.

### Gene annotation

Genome sequences surrounding all significantly associated SNPs were collected and used for gene annotation with BLAST against the IWGSC RefSeq v2.0 genome references for wheat and rice, respectively^97^.

To detect candidate genes affecting lodging‑related traits, regions surrounding traits-associated SNPs were blasted against the wheat genome (IWGSC RefSeq v2.0) in the ensemble genome database using the BLASTn. After alignment, genes exhibiting the highest blast score and identity percentage were selected. The biological process and molecular function of putative genes were detected from ensembl plants (http://plants.ensembl.org). To detect pathways affecting lodging‑related traits, regions surrounding traits-associated SNPs were blasted against the rice genome (IRGSP 1.0) in the ensemble genome database using the BLASTn. Moreover, the sequences of significant SNPs were utilized in the enrichment analysis of gene ontology via KOBAS version 2.0 to test for statistically enriched pathways in the database KEGG^103–105^ [https://www.genome.jp/kegg/; www.kegg.jp/kegg/kegg1.html].

### Genomic prediction strategies

The GP was calculated using three different approaches: RR-BLUP^106^, BRR^107^, and GBLUP^108^. An analysis of all the data was conducted using the Intelligent Prediction and Association Tool (iPat)^109^. A validation set was created by randomly appointing 20% of genotypes to a validation set, and a training set was created by using all the residuals. We repeated this process 100 times for each prediction method. A correlation (r) between the BLUPs and GEBVs was calculated over both the training and validation sets to measure GP accuracy^110–112^.

### Permission for land study

The authors declare that all land experiments and studies were carried out according to authorized rules.

### Supplementary Information

Supplementary Information 1.

Supplementary Information 2.

Supplementary Information 3.

## Data Availability

The datasets generated and analyzed during the current study are available in Supplementary 3.

## Abbreviations

MTAs: Marker-trait associations
GWAS: Genome-wide association study
QTL: Quantitative trait loci
MAF: Minor allele frequency
SNP: Single nucleotide polymorphism
BLUP: Best linear unbiased prediction
GBLUP: Genomic best linear unbiased prediction
RRBLUP: Ridge regression-best linear unbiased prediction
BRR: Bayesian ridge regression
CMLM: Compressed mixed linear model
PCA: Principal component analysis
GP: Genomic prediction
GEBV: Genomic-estimated breeding value
LD: Linkage disequilibrium
PCV: Phenotypic coefficient of variation
GCV: Genotypic coefficient of variation
H^2^: Broad sense heritability
LR: Lodging resistance
LA: Lodged area
CAI: Crop angle of inclination
LS: Lodging score index
PH: Plant height
NFN: Number of nodes
PL: Peduncle length
PeL: Penultimate length
IL1: Internode length 1
IL2: Internode length 2
PD: Peduncle diameter
PeD: Penultimate diameter
ID1: Internode diameter 1
ID2: Internode diameter 2
DTH: Days to heading
DTF: Days to flowering
DTM: Days to maturity
SW: Spike weight
SA: Spike area
GY: Grain yield

## References

[1] Rabieyan E, Alipour H (2021). NGS-based multiplex assay of trait-linked molecular markers revealed the genetic diversity of Iranian bread wheat landraces and cultivars. Crop Pasture Sci..

[2] Rabieyan E, Bihamta MR, Moghaddam ME, Mohammadi V, Alipour H (2022). Imaging-based screening of wheat seed characteristics towards distinguishing drought-responsive Iranian landraces and cultivars. Crop Pasture Sci..

[3] Shah L, Yahya M, Shah SMA (2019). Improving lodging resistance: Using wheat and rice as classical examples. Int. J. Mol. Sci..

[4] Meng B, Wang T, Luo Y (2021). Genome-wide association study identified novel candidate loci/genes affecting lodging resistance in rice. Genes.

[5] Niu L, Feng S, Ding W, Li G (2016). Influence of speed and rainfall on large-scale wheat lodging from 2007 to 2014 in China. PLoS ONE.

[6] Berry PM, Sterling M, Spink JH, Baker CJ, Sylvester-Bradley R, Mooney SJ, Tams AR, Ennos AR (2004). Understanding and reducing lodging in cereals. Adv. Agron..

[7] Yang H, Chen E, Li Z, Zhao C, Yang G, Pignatti S, Casa R, Zhao L (2015). Wheat lodging monitoring using polarimetric index from RADARSAT-2 data. Int. J. Appl. Earth Obs. Geoinf..

[8] Chauhan S, Darvishzadeh R, Boschetti M, Pepe M, Nelson A (2019). Remote sensing-based crop lodging assessment: Current status and perspectives. ISPRS J. Photogramm. Remote Sens..

[9] Huang W, Wang H, Mei D (2018). Progress in research on lodging resistance of crops. Crop Mag..

[10] Piñera-Chavez FJ, Berry PM, Foulkes MJ, Molero G, Reynolds MP (2016). Avoiding lodging in irrigated spring wheat. II. Genetic variation of stem and root structural properties. Field Crop Res..

[11] Wu W, Ma BL (2016). A new method for assessing plant lodging and the impact of management options on lodging in canola crop production. Sci. Rep..

[12] Lang YZ, Yang XD, Wang ME, Zhu QS (2012). Effects of lodging at different filling stages on rice yield and grain quality. Rice Sci..

[13] Berry PM, Sylvester-Bradley R, Berry S (2007). Ideotype design for lodging-resistant wheat. Euphytica.

[14] Zhu G, Li G, Wang D, Yuan S, Wang F (2016). Changes in the lodging-related traits along with rice genetic improvement in China. PLoS ONE.

[15] Nafziger ED, Wax LM, Brown CM (1986). Response of five winter wheat cultivars to growth regulators and increased nitrogen. Crop Sci..

[16] Verma V, Worland AJ, Savers EJ, Fish L, Caligari PD, Snape JW (2005). Identification and characterization of quantitative trait loci related to lodging resistance and associated traits in bread wheat. Plant Breed..

[17] Liu W, Leiser WL, Maurer HP, Li J, Weissmann S, Hahn V, Würschum T (2015). Evaluation of genomic approaches for marker-based improvement of lodging tolerance in triticale. Plant Breed..

[18] Pinthus MJ (1974). Lodging in wheat, barley, and oats: The phenomenon, its causes, and preventive measures. Adv. Agron..

[19] Mwando E, Han Y, Angessa TT, Zhou G, Hill CB, Zhang X-Q, Li C (2020). Genome wide association study of salinity tolerance during germination in barley (*Hordeum vulgare* L.). Front. Plant Sci..

[20] Berry PM, Berry ST (2015). Understanding the genetic control of lodging-associated plant characters in winter wheat (*Triticum aestivum* L.). Euphytica.

[21] Keller M, Karutz C, Schmid JE, Stamp P, Winzeler M, Keller B, Messmer MM (1999). Quantitative trait loci for lodging resistance in a segregating wheat × spelt population. Theor. Appl. Genet..

[22] Hai L, Guo H, Xiao S, Jiang G, Zhang X, Yan C, Jia J (2005). Quantitative trait loci (QTL) of stem strength and related traits in a doubled-haploid population of wheat (*Triticum aestivum* L.). Euphytica.

[23] Singh D, Wang X, Kumar U, Gao L, Noor M, Imtiaz M, Poland J (2019). High-throughput phenotyping enabled genetic dissection of crop lodging in wheat. Front. Plant Sci..

[24] Tumino G, Voorrips RE, Morcia C, Ghizzoni R, Germeier CU, Paulo MJ, Terzi V, Smulders MJ (2017). Genome-wide association analysis for lodging tolerance and plant height in a diverse European hexaploid oat collection. Euphytica.

[25] Resende RT, de Resende MDV, Azevedo CF, Fonsecae Silva F, Melo LC, Pereira HS, Vianello RP (2018). Genome-wide association and regional heritability mapping of plant architecture, lodging and productivity in *Phaseolus vulgaris*. Genes Genom. Genet..

[26] Wei L, Jian H, Lu K, Yin N, Wang J, Duan X, Li J (2017). Genetic and transcriptomic analyses of lignin-and lodging-related traits in *Brassica napus*. Theor. Appl. Genet..

[27] Li H, Cheng X, Zhang L, Hu J, Zhang F, Chen B, Wu X (2018). An integration of genome-wide association study and gene co-expression network analysis identifies candidate genes of stem lodging-related traits in *Brassica napus*. Front. Plant Sci..

[28] Guo Z, Liu X, Zhang B, Yuan X, Xing Y, Liu H, Luo L, Chen G, Xiong L (2021). Genetic analyses of lodging resistance and yield provide insights into post-Green-Revolution breeding in rice. Plant Biotechnol. J..

[29] Arif MAR, Shokat S, Plieske J, Lohwasser U, Chesnokov YV, Kumar N, Kulwal P, McGuire P, Sorrells M, Qualset CO, Börner A (2021). A SNP-based genetic dissection of versatile traits in bread wheat (*Triticum aestivum* L.). Plant J..

[30] Borner A, Plaschke J, Korzun V, Worland AJ (1996). The relationships between the dwarfing genes of wheat and rye. Euphytica.

[31] Snape JW, Law CN, Worland AJ (1977). Whole chromosome analysis of height in wheat. Heredity.

[32] McIntosh, R. A., Dubcovsky, J., Rogers, W. J., Morris, C. & Xia, X. C. *Catalogue of Gene Symbols for Wheat: Supplement (KOMUGI Wheat Genetic Resource Database)* (2017).

[33] Mo Y, Howell T, Vasquez-Gross H, de Haro LA, Dubcovsky J, Pearce S (2018). Mapping causal mutations by exome sequencing in a wheat TILLING population: A tall mutant case study. Mol. Genet. Genomics..

[34] Gasperini D, Greenland A, Hedden P, Dreos R, Harwood W, Griffiths S (2012). Genetic and physiological analysis of Rht8 in bread wheat: An alternative source of semi-dwarfism with a reduced sensitivity to brassinosteroids. J. Exp. Bot..

[35] Korzun V, Röder MS, Ganal MW, Worland AJ, Law CN (1998). Genetic analysis of the dwarfing gene (Rht8) in wheat. Part I. Molecular mapping of Rht8 on the short arm of chromosome 2D of bread wheat (*Triticum aestivum* L.). Theor. Appl. Genet..

[36] Börner A, Schumann E, Fürste A, Cöster H, Leithold B, Röder M, Weber W (2002). Mapping of quantitative trait loci determining agronomic important characters in hexaploid wheat (*Triticum aestivum* L.). Theor. Appl. Genet..

[37] Shabannejad M, Bihamta MR, Majidi-Hervan E, Alipour H, Ebrahimi A (2021). A classic approach for determining genomic prediction accuracy under terminal drought stress and well-watered conditions in wheat landraces and cultivars. PLoS ONE.

[38] Lipka AE, Tian F, Wang QS, Peiffer J, Li M, Bradbury PJ (2012). GAPIT: Genome association and prediction integrated tool. Bioinformatics.

[39] Poland J, Endelman J, Dawson J, Rutkoski J, Wu S, Manes Y, Dreisigacker S, Crossa J, Sánchez-Villeda H, Sorrells M, Jannink JL (2012). Genomic selection in wheat breeding using genotyping-by-sequencing. Plant Genome.

[40] Daetwyler HD, Calus MP, Pong-Wong R, de Los Campos G, Hickey JM (2013). Genomic prediction in animals and plants: Simulation of data, validation, reporting, and benchmarking. Genetics.

[41] Ahmad D, Zhang Z, Rasheed H, Xu X, Bao J (2022). Recent advances in molecular improvement for potato tuber traits. Int. J. Mol. Sci..

[42] Zhao Y, Gowda M, Liu W, Würschum T, Maurer HP, Longin FH, Ranc N, Reif JC (2012). Accuracy of genomic selection in European maize elite breeding populations. Theor. Appl. Genet..

[43] Spindel J, Begum H, Akdemir D, Virk P, Collard B, Redona E, Atlin G, Jannink JL, McCouch SR (2015). Genomic selection and association mapping in rice (*Oryza sativa*): Effect of trait genetic architecture, training population composition, marker number and statistical model on accuracy of rice genomic selection in elite, tropical rice breeding lines. PLoS Genet..

[44] Asoro FG, Newell MA, Beavis WD, Scott MP, Jannink JL (2011). Accuracy and training population design for genomic selection on quantitative traits in elite North American oats. Plant Genome.

[45] Tripathi SC, Sayre KD, Kaul JN, Narang RS (2003). Growth and morphology of spring wheat (*Triticum aestivum* L.) culms and their association with lodging: effects of genotypes, N levels and ethephon. Field Crops Res..

[46] Kelbert AJ, Spaner D, Briggs KG, King JR (2004). Screening for lodging resistance in spring wheat breeding programmes. Plant Breed..

[47] Kong EY, Liu DC, Guo XL, Yang WL, Sun JZ, Li X, Zhan KH, Cui DG, Lin JX, Zhang AM (2013). Anatomical and chemical characteristics associated with lodging resistance in wheat. Crop J..

[48] Sher A, Khan A, Ashraf U, Liu HH, Li JC (2018). Characterization of the effect of increased plant density on canopy morphology and stalk lodging risk. Front. Plant Sci..

[49] Zhu X, Wang X, Guo K, Guo W, Feng C, Peng Y (2006). Stem characteristics of wheat lodging and their effects on Yield and quality. J. Triticeae Crops.

[50] Xiao Y, Liu J, Li H, Cao X, Xia X, He Z (2015). Lodging resistance and yield potential of winter wheat: Effect of planting density and genotype. Front. Agric. Sci. Eng..

[51] Berry PM, Griffin JM, Sylvester-Bradley R, Scott RK, Spink JH, Baker CJ, Clare RW (2000). Controlling plant form through husbandry to minimize lodging in wheat. Field Crops Res..

[52] Berry PM, Sterling M, Baker CJ, Spink J, Sparkes DL (2003). A calibrated model of wheat lodging compared with field measurements. Agric. For. Meteorol..

[53] Wang SX, Zhu YL, Zhang DX, Shao H, Liu P, Hu JB (2017). Genome wide association study for grain yield and related traits in elite wheat varieties and advanced lines using SNP markers. PLoS ONE.

[54] Sul JH, Martin LS, Eskin E (2018). Population structure in genetic studies: Confounding factors and mixed models. PLoS Genet..

[55] Alipour H, Abdi H, Rahimi Y, Bihamta MR (2021). Dissection of the genetic basis of genotype-by-environment interactions for grain yield and main agronomic traits in Iranian bread wheat landraces and cultivars. Sci. Rep..

[56] Sabzehzari M, Zeinali M, Naghavi MR (2020). Alternative sources and metabolic engineering of Taxol: Advances and future perspectives. Biotechnol. Adv..

[57] Mourad AMI, Belamkar V, Baenziger PS (2020). Molecular genetic analysis of spring wheat core collection using genetic diversity, population structure, and linkage disequilibrium. BMC Genomics.

[58] Alipour H, Bihamta MR, Mohammadi V, Peyghambari SA, Bai G, Zhang G (2017). Genotyping-by-sequencing (GBS) revealed molecular genetic diversity of Iranian wheat landraces and cultivars. Front. Plant Sci..

[59] Albrecht T, Oberforster M, Kempf H, Ramgraber L, Schacht J, Kazman E (2015). Genome wide association mapping of pre-harvest sprouting resistance in a diversity panel of European winter wheat. J. Appl. Genet..

[60] Liu H, Zhou H, Wu Y, Li X, Zhao J, Zuo T, Zhang X, Zhang Y, Liu S, Shen Y (2015). The impact of genetic relationship and linkage disequilibrium on genomic selection. PLoS ONE.

[61] Liu J, Rasheed A, He Z, Imtiaz M, Arif A, Mahmood T, Xia X (2019). Genome-wide variation patterns between landraces and cultivars uncover divergent selection during modern wheat breeding. Theor. Appl. Genet..

[62] Rabbi SMHA, Kumar A, Mohajeri Naraghi S, Simsek S, Sapkota S, Solanki S, Alamri MS, Elias EM, Kianian S, Missaoui A, Mergoum M (2021). Genome-wide association mapping for yield and related traits under drought stressed and non-stressed environments in wheat. Front. Genet..

[63] Bhatta M, Morgounov A, Belamkar V, Baenziger PS (2018). Genome-wide association study reveals novel genomic regions for grain yield and yield-related traits in drought-stressed synthetic hexaploid wheat. Int. J. Mol. Sci..

[64] Gahlaut V, Jaiswal V, Singh S (2019). Multi-locus genome wide association mapping for yield and its contributing traits in hexaploid wheat under different water regimes. Sci. Rep..

[65] Esmaeili-Fard SM, Gholizadeh M, Hafezian SH, Abdollahi-Arpanahi R (2021). Genes and pathways affecting sheep productivity traits: Genetic parameters, genome-wide association mapping, and pathway enrichment analysis. Front. Genet..

[66] Griffiths S, Simmonds J, Leverington M, Wang Y, Fish L, Sayers L, Alibert L, Orford S, Wingen L, Snape J (2012). Meta-QTL analysis of the genetic control of crop height in elite European winter wheat germplasm. Mol. Breed..

[67] Neumann K, Kobiljski B, Denčić S, Varshney R, Börner A (2011). Genome-wide association mapping: a case study in bread wheat (*Triticum aestivum* L.). Mol. Breed..

[68] Rahimi Y, Bihamta MR, Taleei A, Alipour H, Ingvarsson PK (2019). Genome-wide association study of agronomic traits in bread wheat reveals novel putative alleles for future breeding programs. BMC Plant Biol..

[69] Bordes J, Goudemand E, Duchalais L, Chevarin L, Oury FX (2014). Genome-wide association mapping of three important traits using bread wheat elite breeding populations. Mol. Breed..

[70] Sukumaran S, Lopes M, Dreisigacker S, Reynolds M (2018). Genetic analysis of multi-environmental spring wheat trials identify genomic regions for locus-specific trade-offs for grain weight and grain number. Theor. Appl. Genet..

[71] Kumar N, Kulwal PL, Balyan HS, Gupta PK (2007). QTL mapping for yield and yield contributing traits in two mapping populations of bread wheat. Mol. Breed..

[72] Edae EA, Byrne PF, Haley SD, Lopes MS, Reynolds MP (2014). Genome-wide association mapping of yield and yield components of spring wheat under contrasting moisture regimes. Theor. Appl. Genet..

[73] Hoffstetter A, Cabrera A, Sneller C (2016). Identifying quantitative trait loci for economic traits in an elite soft red winter wheat population. Crop Sci..

[74] Sehgal D, Autrique E, Singh R, Ellis M, Singh S, Dreisigacker S (2017). Identification of genomic regions for grain yield and yield stability and their epistatic interactions. Sci. Rep..

[75] Ogbonnaya FC, Rasheed A, Okechukwu EC, Jighly A, Makdis F, Wuletaw T, Hagras A, Uguru MI, Agbo CU (2017). Genome-wide association study for agronomic and physiological traits in spring wheat evaluated in a range of heat prone environments. Theor. Appl. Genet..

[76] Lozada DN, Mason RE, Babar MA, Carver BF, Guedira GB, Merrill K, Arguello MN, Acuna A, Vieira L, Holder A (2017). Association mapping reveals loci associated with multiple traits that affect grain yield and adaptation in soft winter wheat. Euphytica.

[77] Appels R, Eversole K, Stein N, Feuillet C, Keller B, Rogers J, Pozniak CJ, Choulet F, Distelfeld A, Poland J (2018). Shifting the limits in wheat research and breeding using a fully annotated reference genome. Science.

[78] Kuai J, Li X, Ji J, Li Z, Xie Y, Wang B, Zhou G (2021). The physiological and proteomic characteristics of oilseed rape stem affect seed yield and lodging resistance under different planting densities and row spacing. J. Agron. Crop Sci..

[79] Tian QL, Liu B, Zhong XY, Zhao M, Sun H, Ren WJ (2016). Relationship of NSC with the formation of branches and spikelets and the yield traits of Indica hybrid rice in different planting methods. Sci. Agric. Sin..

[80] Slewinski TL (2012). Non-structural carbohydrate partitioning in grass stems: A target to increase yield stability, stress tolerance, and biofuel production. J. Exp. Bot..

[81] Fu J, Huang Z, Wang Z, Yang J, Zhang J (2011). Pre-anthesis non-structural carbohydrate reserve in the stem enhances the sink strength of inferior spikelets during grain filling of rice. Field Crops Res..

[82] Mizuno H, Kasuga S, Kawahigashi H (2018). Root lodging is a physical stress that changes gene expression from sucrose accumulation to degradation in sorghum. BMC Plant Biol..

[83] Cai T, Meng X, Liu X, Liu T, Wang H, Jia Z, Ren X (2018). Exogenous hormonal application regulates the occurrence of wheat tillers by changing endogenous hormones. Front. Plant Sci..

[84] Raza A, Asghar MA, Ahmad B, Bin C, Iftikhar Hussain M, Li W, Weiguo L (2020). Agro-techniques for lodging stress management in maize-soybean intercropping system: A review. Plants.

[85] Thorwarth P, Ahlemeyer J, Bochard AM, Krumnacker K, Blümel H, Laubach E, Knöchel N, Cselényi L, Ordon F, Schmid KJ (2017). Genomic prediction ability for yield-related traits in German winter barley elite material. Theor. Appl. Genet..

[86] Crossa J, Jarquín D, Franco J, Pérez-Rodríguez P, Burgueño J, Saint-Pierre C, Vikram P, Sansaloni C, Petroli C, Akdemir D, Sneller C (2016). Genomic prediction of gene bank wheat landraces. Genes Genom. Genet..

[87] de Azevedo Peixoto L, Moellers TC, Zhang J, Lorenz AJ, Bhering LL, Beavis WD, Singh AK (2017). Leveraging genomic prediction to scan germplasm collection for crop improvement. PLoS ONE.

[88] Kehel Z, Sanchez-Garcia M, El Baouchi A, Aberkane H, Tsivelikas A, Charles C, Amri A (2020). Predictive characterization for seed morphometric traits for genebank accessions using genomic selection. Front. Ecol. Evol..

[89] Norman A, Taylor J, Edwards J, Kuchel H (2018). Optimising genomic selection in wheat: Effect of marker density, population size and population structure on prediction accuracy. Genes Genom. Genet..

[90] Daetwyler HD, Bansal UK, Bariana HS, Hayden MJ, Hayes BJ (2014). Genomic prediction for rust resistance in diverse wheat landraces. Theor. Appl. Genet..

[91] Chauhan S, Darvishzadeh R, Boschetti M, Nelson A (2020). Estimation of crop angle of inclination for lodged wheat using multi-sensor SAR data. Remote Sens. Environ..

[92] Rabieyan E, Bihamta MR, Mostashari MM, Moghaddam ME, Mohammadi V, Alipour H (2023). Applying genetic biofortification for screening of Iranian bread wheat genotypes with high grain yield and nutritional quality. J. Soil Sci. Plant Nutr..

[93] Mavindidze P, Mafandizvo T, Gasura E, Mavankeni BO, Kutywayo D, Kamutando CN (2020). Progress check of yielding ability and stability of selected pre-release bread-wheat cultivars in Zimbabwe. J. Crop Sci. Biotechnol..

[94] Saghai-Maroof MA, Soliman KM, Jorgensen RA, Allard R (1984). Ribosomal DNA spacer-length polymorphisms in barley: Mendelian inheritance, chromosomal location, and population dynamics. PNAS.

[95] Elshire RJ, Glaubitz JC, Sun Q, Poland JA, Kawamoto K, Buckler ES, Mitchell SE (2011). A robust, simple genotyping-by-sequencing (GBS) approach for high diversity species. PLoS ONE.

[96] Bradbury PJ, Zhang Z, Kroon DE, Casstevens TM, Ramdoss Y, Buckler ES (2007). TASSEL: Software for association mapping of complex traits in diverse samples. Bioinformatics.

[97] Alipour H, Bai G, Zhang G, Bihamta MR, Mohammadi V, Peyghambari SA (2019). Imputation accuracy of wheat genotyping-by-sequencing (GBS) data using barley and wheat genome references. PLoS ONE.

[98] Pritchard JK, Stephens M, Donnelly P (2000). Inference of population structure using multilocus genotype data. Genetics.

[99] Evanno G, Regnaut S, Goudet J (2005). Detecting the number of clusters of individuals using the software STRUCTURE: A simulation study. Mol. Ecol..

[100] Kang HM, Zaitlen NA, Wade CM, Kirby A, Heckerman D, Daly MJ, Eskin E (2008). Efficient control of population structure in model organism association mapping. Genetics.

[101] Lipka AE, Tian F, Wang Q, Peiffer J, Li M, Bradbury PJ, Gore MA, Buckler ES, Zhang Z (2012). GAPIT: Genome association and prediction integrated tool. Bioinformatics.

[102] Remington DL, Thornsberry JM, Matsuoka Y, Wilson LM, Whitt SR, Doebley J, Kresovich S, Goodman MM, Buckler ES (2001). Structure of linkage disequilibrium and phenotypic associations in the maize genome. PNAS.

[103] Kanehisa M, Goto S (2000). KEGG: Kyoto encyclopedia of genes and genomes. Nucleic Acids Res..

[104] Kanehisa M (2019). Toward understanding the origin and evolution of cellular organisms. Protein Sci..

[105] Kanehisa M, Furumichi M, Sato Y, Ishiguro-Watanabe M, Tanabe M (2021). KEGG: Integrating viruses and cellular organisms. Nucleic Acids Res..

[106] Endelman JB (2011). Ridge regression and other kernels for genomic selection with R package rrBLUP. Plant Genome.

[107] Pérez P, de Los Campos G (2014). Genome-wide regression and prediction with the BGLR statistical package. Genetics.

[108] Clark SA, van der Werf J (2013). Genomic best linear unbiased prediction (gBLUP) for the estimation of genomic breeding values. Genom. Wide Assoc. Stud. Genom. Pred..

[109] Chen CJ, Zhang Z (2018). iPat: Intelligent prediction and association tool for genomic research. Bioinformatics.

[110] Resende Jr MFR, Munoz P, Resende MD, Garrick DJ, Fernando RL, Davis JM, Jokela EJ, Martin TA, Peter GF, Kirst M (2012). Accuracy of genomic selection methods in a standard data set of loblolly pine (*Pinus taeda* L.). Genetics.

[111] Rabieyan E, Bihamta MR, Moghaddam ME, Mohammadi V, Alipour H (2022). Genome-wide association mapping and genomic prediction for pre-harvest sprouting resistance, low α-amylase and seed color in Iranian bread wheat. BMC Plant Biol..

[112] Rabieyan E, Bihamta MR, Moghaddam ME, Mohammadi V, Alipour H (2022). Genome-wide association mapping and genomic prediction of agronomical traits and breeding values in Iranian wheat under rain-fed and well-watered conditions. BMC Genom..

