# Supplementary material for: Genetic analyses and prediction for lodging‑related traits in a diverse Iranian hexaploid wheat collection
Source: Sci Rep. 2024 Jan 2;14:275. doi: 10.1038/s41598-023-49927-z (PMC10761700; doi:10.1038/s41598-023-49927-z)
Supplement: Supplementary file 1 — Supplementary Information 1. [file 41598_2023_49927_MOESM1_ESM.docx]

**Supplementary Table 1** Mean, heritability coefficient of variation (CV), and combined analysis of variance based on studied traits in 298 Iranian wheat landraces and cultivars.

| **Trait** | **Mean** | **CV (%)** | **PCV (%)** | **GCV (%)** | **H^2^ (%)** | **Mean squares** | | | |
| --- | --- | --- | --- | --- | --- | --- | --- | --- | --- |
|  |  |  |  |  |  | **Env** | **Rep (Env)** | **Gen** | **Gen×Env** |
| **LA** | 45.41 | 12.91 | 22.15 | 16.11 | 75.55 | ns | ns | *** | *** |
| **CAL** | 40.39 | 13.22 | 18.89 | 13.48 | 77.09 | * | * | *** | *** |
| **LS** | 0.30 | 15.05 | 24.95 | 20.58 | 72.82 | ns | ns | *** | *** |
| **PH** | 103.41 | 12.08 | 15.81 | 11.46 | 77.07 | * | * | *** | *** |
| **NFN** | 4.78 | 5.23 | 6.03 | 4.48 | 88.25 | ns | ns | *** | *** |
| **PL** | 41.11 | 5.23 | 10.57 | 8.42 | 79.22 | ns | * | *** | *** |
| **PeL** | 24.55 | 4.39 | 8.97 | 7.86 | 81.86 | * | * | *** | *** |
| **IL2** | 11.80 | 11.52 | 14.78 | 11.74 | 86.37 | ns | * | *** | *** |
| **IL1** | 7.74 | 12.54 | 14.54 | 12.54 | 78.33 | ns | ns | *** | *** |
| **PD** | 3.24 | 11.95 | 13.54 | 10.76 | 81.37 | ns | * | *** | *** |
| **PeL** | 3.99 | 11.11 | 13.54 | 10.88 | 86.08 | ns | ns | *** | *** |
| **ID2** | 4.11 | 10.07 | 10.87 | 7.57 | 80.98 | * | ** | *** | *** |
| **ID1** | 3.93 | 12.12 | 13.65 | 11.76 | 86.47 | ns | ns | *** | *** |
| **DTH** | 182.83 | 3.45 | 9.38 | 6.25 | 85.84 | * | * | *** | *** |
| **DTF** | 190.48 | 3.29 | 9.96 | 8.66 | 83.39 | * | ** | *** | *** |
| **DTM** | 219.18 | 2.82 | 9.44 | 7.36 | 79.10 | * | ** | *** | *** |
| **SW** | 2.29 | 21.50 | 26.99 | 22.11 | 65.54 | * | * | *** | *** |
| **SA** | 12.13 | 19.14 | 22.99 | 17.11 | 58.86 | * | ** | *** | *** |
| **GY** | 1.71 | 20.74 | 27.34 | 23.05 | 41.32 | * | * | *** | *** |

*, ** and *** are signifcant at the probability level of 5%, 1% and 0.1%, respectively.

Abbreviations: Lodged area (LA, %), crop angle of inclination (CAI), lodging score index (LS), plant height (PH, cm), number of nodes (NFN), peduncle length (PL, cm), penultimate length (PeL, cm), internode length 1 (IL1, cm), internode length 2 (IL2, cm), peduncle diameter (PD, mm), penultimate diameter (PeD, mm), internode diameter 1 (ID1, mm), internode diameter 2 (ID2, mm), days to heading (DTH), days to flowering (DTF), days to maturity (DTM), spike weight (SW, g), spike area (SA, cm^2^) and grain yield (GY, g per plant).

**Supplementary Table 2** Descriptive statistics for 19 lodging-related traits in Iranian wheat cultivars and landraces.

| **Trait** | **Type** | **N** | **Mean** | **Minimum** | **Maximum** | **Std Error** | **Skewness** | **Kurtosis** |
| --- | --- | --- | --- | --- | --- | --- | --- | --- |
| **CAL** | Cultivar | 90 | 15.352 | 0 | 76.7 | 2.439 | 1.378 | 0.631 |
|  | Landrace | 208 | 51.217 | 0 | 79.2 | 1.501 | -0.829 | -0.052 |
|  | Total | 298 | 40.385 | 0 | 79.2 | 1.596 | -0.294 | -1.341 |
| **LA** | Cultivar | 90 | 16.491 | 0 | 100 | 2.819 | 1.575 | 1.389 |
|  | Landrace | 208 | 57.923 | 0 | 100 | 1.892 | -0.649 | -0.492 |
|  | Total | 298 | 45.41 | 0 | 100 | 1.918 | -0.149 | -1.403 |
| **LS** | Cultivar | 90 | 0.094 | 0 | 0.8 | 0.02 | 2.403 | 5.125 |
|  | Landrace | 208 | 0.389 | 0 | 0.84 | 0.017 | 0.004 | -1.167 |
|  | Total | 298 | 0.3 | 0 | 0.84 | 0.016 | 0.391 | -1.217 |
| **PH** | Cultivar | 90 | 94.118 | 72.64 | 121.39 | 1.293 | 0.598 | -0.507 |
|  | Landrace | 208 | 107.434 | 76.62 | 127.36 | 0.712 | -0.579 | 0.196 |
|  | Total | 298 | 103.412 | 72.64 | 127.36 | 0.724 | -0.35 | -0.734 |
| **NFN** | Cultivar | 90 | 4.367 | 4 | 6 | 0.053 | 0.825 | -0.716 |
|  | Landrace | 208 | 4.957 | 4 | 6 | 0.036 | -0.056 | 0.685 |
|  | Total | 298 | 4.779 | 4 | 6 | 0.034 | 0.078 | -0.391 |
| **PL** | Cultivar | 90 | 38.775 | 25.74 | 53.47 | 0.584 | 0.556 | 0.389 |
|  | Landrace | 208 | 42.114 | 26.73 | 58.42 | 0.436 | 0.093 | -0.511 |
|  | Total | 298 | 41.106 | 25.74 | 58.42 | 0.363 | 0.245 | -0.439 |
| **PeL** | Cultivar | 90 | 22.924 | 16.83 | 33.66 | 0.356 | 0.6 | 0.476 |
|  | Landrace | 208 | 25.259 | 13.86 | 35.64 | 0.233 | -0.393 | 0.369 |
|  | Total | 298 | 24.554 | 13.86 | 35.64 | 0.205 | -0.106 | -0.18 |
| **IL2** | Cultivar | 90 | 10.118 | 6 | 16.67 | 0.276 | 0.558 | -0.549 |
|  | Landrace | 208 | 12.529 | 6 | 17.33 | 0.148 | -0.396 | 0.241 |
|  | Total | 298 | 11.801 | 6 | 17.33 | 0.147 | -0.268 | -0.585 |
| **IL1** | Cultivar | 90 | 6.334 | 4 | 10.67 | 0.17 | 0.81 | -0.102 |
|  | Landrace | 208 | 8.353 | 4 | 11.56 | 0.098 | -0.397 | 0.249 |
|  | Total | 298 | 7.743 | 4 | 11.56 | 0.101 | -0.228 | -0.728 |
| **PD** | Cultivar | 90 | 3.441 | 2.19 | 4.9 | 0.056 | 0.465 | 0.667 |
|  | Landrace | 208 | 3.146 | 1.41 | 4.94 | 0.037 | 0.119 | 1.484 |
|  | Total | 298 | 3.235 | 1.41 | 4.94 | 0.032 | 0.211 | 1.199 |
| **PeD** | Cultivar | 90 | 4.332 | 2.93 | 5.65 | 0.058 | -0.148 | -0.282 |
|  | Landrace | 208 | 3.841 | 2.24 | 5.27 | 0.039 | -0.095 | -0.112 |
|  | Total | 298 | 3.99 | 2.24 | 5.65 | 0.035 | -0.069 | -0.187 |
| **ID2** | Cultivar | 90 | 4.357 | 3.34 | 5.52 | 0.047 | 0.121 | 0.05 |
|  | Landrace | 208 | 4.005 | 2.41 | 5.8 | 0.037 | -0.158 | 0.892 |
|  | Total | 298 | 4.112 | 2.41 | 5.8 | 0.031 | -0.195 | 0.729 |
| **ID1** | Cultivar | 90 | 4.226 | 2.48 | 5.7 | 0.051 | -0.124 | 1.814 |
|  | Landrace | 208 | 3.807 | 2.4 | 5.4 | 0.041 | 0.056 | -0.429 |
|  | Total | 298 | 3.934 | 2.4 | 5.7 | 0.034 | -0.104 | -0.176 |
| **DTH** | Cultivar | 90 | 178.833 | 163 | 196.5 | 0.675 | 0.173 | 0.108 |
|  | Landrace | 208 | 184.558 | 171 | 196.5 | 0.376 | -0.377 | -0.441 |
|  | Total | 298 | 182.829 | 163 | 196.5 | 0.365 | -0.343 | -0.332 |
| **DTF** | Cultivar | 90 | 186.567 | 170.5 | 203.5 | 0.675 | 0.229 | 0.006 |
|  | Landrace | 208 | 192.171 | 177.5 | 204 | 0.375 | -0.361 | -0.431 |
|  | Total | 298 | 190.478 | 170.5 | 204 | 0.363 | -0.316 | -0.396 |
| **DTM** | Cultivar | 90 | 215.356 | 199 | 229.5 | 0.661 | 0.008 | -0.155 |
|  | Landrace | 208 | 220.837 | 208 | 234 | 0.371 | -0.297 | -0.405 |
|  | Total | 298 | 219.181 | 199 | 234 | 0.357 | -0.347 | -0.244 |
| **SW** | Cultivar | 90 | 2.412 | 1.21 | 4.15 | 0.056 | 0.475 | 0.652 |
|  | Landrace | 208 | 2.243 | 1.12 | 3.84 | 0.033 | 0.525 | 0.29 |
|  | Total | 298 | 2.294 | 1.12 | 4.15 | 0.029 | 0.539 | 0.466 |
| **SA** | Cultivar | 90 | 13.19 | 8.39 | 18.15 | 0.201 | 0.029 | -0.185 |
|  | Landrace | 208 | 11.667 | 6.88 | 20.49 | 0.162 | 0.78 | 0.907 |
|  | Total | 298 | 12.127 | 6.88 | 20.49 | 0.134 | 0.439 | 0.164 |
| **GY** | Cultivar | 90 | 1.89 | 1.09 | 2.71 | 0.034 | 0.269 | -0.145 |
|  | Landrace | 208 | 1.629 | 0.82 | 2.75 | 0.023 | 0.294 | -0.154 |
|  | Total | 298 | 1.708 | 0.82 | 2.75 | 0.02 | 0.247 | -0.184 |

Abbreviations: Lodged area (LA, %), crop angle of inclination (CAI), lodging score index (LS), plant height (PH, cm), number of nodes (NFN), peduncle length (PL, cm), penultimate length (PeL, cm), internode length 1 (IL1, cm), internode length 2 (IL2, cm), peduncle diameter (PD, mm), penultimate diameter (PeD, mm), internode diameter 1 (ID1, mm), internode diameter 2 (ID2, mm), days to heading (DTH), days to flowering (DTF), days to maturity (DTM), spike weight (SW, g), spike area (SA, cm^2^) and grain yield (GY, g per plant).

**Supplementary Table** Correlation coefficients between two environments (year 1 and year 2) for lodging-related traits ‎in Iranian wheat cultivars and landraces.

|  |  |  |  |  |  | Year 1 | | | | | | | | | | | |  | |  | |  | |  | |
| --- | --- | --- | --- | --- | --- | --- | --- | --- | --- | --- | --- | --- | --- | --- | --- | --- | --- | --- | --- | --- | --- | --- | --- | --- | --- |
|  |  | LA | CAL | LS | PH | NFN | PL | PeL | IL2 | IL1 | PD | PeL | ID2 | ID1 | DTH | DTF | DTM | | SW | | SA | | GY | |  |
|  | LA | 0.918^**^ |  |  |  |  |  |  |  |  |  |  |  |  |  |  |  | |  | |  | |  | |  |
|  | CAL | 0.749^**^ | 0.886^**^ |  |  |  |  |  |  |  |  |  |  |  |  |  |  | |  | |  | |  | |  |
|  | LS | 0.747^**^ | 0.722^**^ | 0.881^**^ |  |  |  |  |  |  |  |  |  |  |  |  |  | |  | |  | |  | |  |
|  | PH | 0.625^**^ | 0.639^**^ | 0.533^**^ | 0.847^**^ |  |  |  |  |  |  |  |  |  |  |  |  | |  | |  | |  | |  |
|  | NFN | 0.583^**^ | 0.591^**^ | 0.498^**^ | 0.824^**^ | 0.928^**^ |  |  |  |  |  |  |  |  |  |  |  | |  | |  | |  | |  |
|  | PL | 0.232^**^ | 0.240^**^ | 0.179^**^ | 0.445^**^ | 0.361^**^ | 0.851^**^ |  |  |  |  |  |  |  |  |  |  | |  | |  | |  | |  |
|  | PeL | 0.409^**^ | 0.426^**^ | 0.390^**^ | 0.553^**^ | 0.397^**^ | 0.333^**^ | 0.843^**^ |  |  |  |  |  |  |  |  |  | |  | |  | |  | |  |
|  | IL2 | 0.508^**^ | 0.532^**^ | 0.444^**^ | 0.758^**^ | 0.636^**^ | 0.119^*^ | 0.456^**^ | 0.873^**^ |  |  |  |  |  |  |  |  | |  | |  | |  | |  |
| Year 2 | IL1 | 0.574^**^ | 0.592^**^ | 0.504^**^ | 0.730^**^ | 0.599^**^ | 0.135^*^ | 0.464^**^ | 0.716^**^ | 0.864^**^ |  |  |  |  |  |  |  | |  | |  | |  | |  |
|  | PD | -0.286^**^ | -0.251^**^ | -0.259^**^ | -0.167^**^ | -0.184^**^ | 0.035 | -0.048 | -0.170^**^ | -0.181^**^ | 0.915^**^ |  |  |  |  |  |  | |  | |  | |  | |  |
|  | PeL | -0.281^**^ | -0.242^**^ | -0.253^**^ | -0.172^**^ | -0.178^**^ | 0.032 | -0.046 | -0.173^**^ | -0.175^**^ | 0.760^**^ | 0.933^**^ |  |  |  |  |  | |  | |  | |  | |  |
|  | ID2 | -0.349^**^ | -0.342^**^ | -0.315^**^ | -0.222^**^ | -0.223^**^ | -0.009 | -0.089 | -0.247^**^ | -0.269^**^ | 0.515^**^ | 0.489^**^ | 0.851^**^ |  |  |  |  | |  | |  | |  | |  |
|  | ID1 | -0.439^**^ | -0.451^**^ | -0.406^**^ | -0.218^**^ | -0.194^**^ | -0.060 | -0.103 | -0.261^**^ | -0.286^**^ | 0.493^**^ | 0.467^**^ | 0.716^**^ | 0.810^**^ |  |  |  | |  | |  | |  | |  |
|  | DTH | 0.367^**^ | 0.388^**^ | 0.306^**^ | 0.371^**^ | 0.349^**^ | 0.163^**^ | 0.151^**^ | 0.377^**^ | 0.371^**^ | -0.114^*^ | -0.115^*^ | -0.194^**^ | -0.222^**^ | 0.917^**^ |  |  | |  | |  | |  | |  |
|  | DTF | 0.351^**^ | 0.372^**^ | 0.287^**^ | 0.399^**^ | 0.374^**^ | 0.148^*^ | 0.128^*^ | 0.392^**^ | 0.380^**^ | -0.139^*^ | -0.142^*^ | -0.218^**^ | -0.220^**^ | 0.743^**^ | 0.916^**^ |  | |  | |  | |  | |  |
|  | DTM | 0.348^**^ | 0.364^**^ | 0.291^**^ | 0.366^**^ | 0.343^**^ | 0.140^*^ | 0.136^*^ | 0.365^**^ | 0.359^**^ | -0.112 | -0.116^*^ | -0.192^**^ | -0.201^**^ | 0.743^**^ | 0.745^**^ | 0.937^**^ | |  | |  | |  | |  |
|  | SW | -0.177^**^ | -0.206^**^ | -0.160^**^ | -0.140^*^ | -0.117^*^ | 0.014 | -0.045 | -0.137^*^ | -0.172^**^ | 0.233^**^ | 0.203^**^ | 0.270^**^ | 0.250^**^ | -0.087 | -0.088 | -0.106 | | 0.875^**^ | |  | |  | |  |
|  | SA | -0.298^**^ | -0.297^**^ | -0.270^**^ | -0.111 | -0.085 | -0.138^*^ | -0.054 | -0.175^**^ | -0.188^**^ | 0.295^**^ | 0.283^**^ | 0.332^**^ | 0.385^**^ | -0.256^**^ | -0.249^**^ | -0.252^**^ | | 0.262^**^ | | 0.804^**^ | |  | |  |
|  | GY | -0.222^**^ | -0.257^**^ | -0.183^**^ | -0.190^**^ | -0.157^**^ | -0.043 | -0.048 | -0.260^**^ | -0.248^**^ | 0.264^**^ | 0.242^**^ | 0.348^**^ | 0.360^**^ | -0.299^**^ | -0.311^**^ | -0.314^**^ | | 0.501^**^ | | 0.391^**^ | | 0.832^**^ | |  |

*, ** and *** are signifcant at the probability level of 5%, 1% and 0.1%, respectively.

**Supplementary Table 4** A summary of LD observed among marker pairs and the number of significant marker pairs per genome and chromosome.

| **Chromosome** | **Total** | | | |  | **Landrace** | | | |  | **Cultivar** | | | |
| --- | --- | --- | --- | --- | --- | --- | --- | --- | --- | --- | --- | --- | --- | --- |
|  | **TNSP** | **r^2^** | **Dis. (cM)** | **NSSP** |  | **TNSP** | **r^2^** | **Dis. (cM)** | **NSSP** |  | **TNSP** | **r^2^** | **Dis. (cM)** | **NSSP** |
| 1A | 111575 | 0.111829 | 1.333712 | 49917 (44.74%) |  | 94575 | 0.116906 | 1.568634 | 34895 (36.9%) |  | 85625 | 0.148069 | 1.736676 | 27111 (31.66%) |
| 2A | 137150 | 0.251605 | 0.856962 | 79772 (58.16%) |  | 125450 | 0.289098 | 0.936772 | 68972 (54.98%) |  | 119450 | 0.288518 | 0.972951 | 57769 (48.36%) |
| 3A | 96450 | 0.130453 | 2.27878 | 44914 (46.57%) |  | 74950 | 0.134097 | 2.933748 | 28787 (38.41%) |  | 85000 | 0.15728 | 2.574908 | 25912 (30.48%) |
| 4A | 130500 | 0.317779 | 1.378513 | 79428 (60.86%) |  | 110850 | 0.369392 | 1.594492 | 66016 (59.55%) |  | 116700 | 0.36745 | 1.50704 | 58086 (49.77%) |
| 5A | 71850 | 0.132927 | 2.005721 | 32488 (45.22%) |  | 60100 | 0.146486 | 2.402626 | 24483 (40.74%) |  | 60600 | 0.166755 | 2.38547 | 18725 (30.9%) |
| 6A | 99050 | 0.158856 | 1.296073 | 52549 (53.05%) |  | 85850 | 0.178539 | 1.498357 | 40739 (47.45%) |  | 86550 | 0.178744 | 1.486057 | 29651 (34.26%) |
| 7A | 149700 | 0.193545 | 1.164988 | 78616 (52.52%) |  | 128550 | 0.211862 | 1.358487 | 64114 (49.87%) |  | 129900 | 0.232161 | 1.343972 | 49454 (38.07%) |
| 1B | 150800 | 0.154279 | 0.932852 | 80419 (53.33%) |  | 135600 | 0.154625 | 1.035051 | 64442 (47.52%) |  | 132400 | 0.20421 | 1.063407 | 49705 (37.54%) |
| 2B | 187300 | 0.156885 | 0.764253 | 102236 (54.58%) |  | 157350 | 0.176011 | 0.910909 | 79057 (50.24%) |  | 166950 | 0.19665 | 0.858127 | 66140 (39.62%) |
| 3B | 201700 | 0.210733 | 0.771726 | 119399 (59.2%) |  | 173200 | 0.220043 | 0.89872 | 90266 (52.12%) |  | 177550 | 0.243607 | 0.876084 | 78180 (44.03%) |
| 4B | 60050 | 0.115027 | 2.20477 | 23537 (39.2%) |  | 44800 | 0.09777 | 2.968273 | 12423 (27.73%) |  | 52600 | 0.142347 | 2.516753 | 13477 (25.62%) |
| 5B | 152400 | 0.15014 | 1.292476 | 80669 (52.93%) |  | 136300 | 0.14202 | 1.445522 | 57252 (42%) |  | 135650 | 0.202818 | 1.431617 | 55651 (41.03%) |
| 6B | 190850 | 0.13708 | 0.658245 | 99314 (52.04%) |  | 167500 | 0.135522 | 0.750676 | 71975 (42.97%) |  | 159700 | 0.203568 | 0.787671 | 66038 (41.35%) |
| 7B | 150100 | 0.121987 | 0.987127 | 70107 (46.71%) |  | 127550 | 0.12878 | 1.153868 | 51602 (40.46%) |  | 134150 | 0.155388 | 1.102364 | 41168 (30.69%) |
| 1D | 48650 | 0.238268 | 3.477302 | 26009 (53.46%) |  | 42500 | 0.226198 | 3.808863 | 20075 (47.24%) |  | 38350 | 0.285881 | 4.409069 | 16564 (43.19%) |
| 2D | 69550 | 0.183692 | 1.586178 | 31547 (45.36%) |  | 55400 | 0.163933 | 1.999469 | 21117 (38.12%) |  | 49600 | 0.228564 | 2.23156 | 16357 (32.98%) |
| 3D | 37050 | 0.116765 | 4.639072 | 5460 (14.74%) |  | 31800 | 0.165445 | 5.245984 | 11619 (36.54%) |  | 26800 | 0.137566 | 6.273779 | 5458 (20.37%) |
| 4D | 13500 | 0.122822 | 9.104484 | 4560 (33.78%) |  | 11800 | 0.130958 | 10.56137 | 3577 (30.31%) |  | 11550 | 0.154924 | 10.56621 | 2312 (20.02%) |
| 5D | 31750 | 0.130873 | 6.894582 | 12308 (38.77%) |  | 26250 | 0.134737 | 8.311197 | 9238 (35.19%) |  | 23700 | 0.147915 | 9.317761 | 5518 (23.28%) |
| 6D | 38300 | 0.123729 | 4.134238 | 15652 (40.87%) |  | 34900 | 0.136001 | 4.545476 | 12619 (36.16%) |  | 29750 | 0.137805 | 5.369092 | 6852 (23.03%) |
| 7D | 46700 | 0.150286 | 4.409549 | 17838 (38.2%) |  | 42300 | 0.147515 | 4.882439 | 14457 (34.18%) |  | 35850 | 0.201644 | 5.778975 | 10863 (30.3%) |
| A genome | 796275 | 0.195029 | 1.397647 | 417684 (52.45%) |  | 680325 | 0.220024 | 1.631824 | 328006 (48.21%) |  | 683825 | 0.232699 | 1.61945 | 266708 (39%) |
| B genome | 1093200 | 0.154972 | 0.95375 | 575681 (52.66%) |  | 942300 | 0.1588 | 1.106081 | 427017 (45.32%) |  | 959000 | 0.199661 | 1.084318 | 370359 (38.62%) |
| D genome | 285500 | 0.162046 | 4.054108 | 113374 (39.71%) |  | 244950 | 0.1634 | 4.684331 | 92702 (37.85%) |  | 215600 | 0.197637 | 5.369609 | 63924 (29.65%) |
| Whole genomes | 2174975 | 0.170566 | 1.523235 | 1106739 (50.89%) |  | 1867575 | 0.181706 | 1.766921 | 847725 (45.39%) |  | 1858425 | 0.211583 | 1.778371 | 700991 (37.72%) |

Abbreviations: r^2^, Average squared allele frequency correlation; TNSP, Total number of SNP pairs; NSSP, Number of significant SNP pairs (P<0.001); Dis, Distance.

**Supplementary Table 5** Description of expected MTAs using imputed SNPs for lodging traits in Iranian wheat accessions.

| **No** | **SNP** | **Sequence** | **Trait- Index** | **Chromosome** | **Position (bp)** | **P-Value** | **Transcript ID** | **R^2^** | **Molecular process** | **Biological process** |
| --- | --- | --- | --- | --- | --- | --- | --- | --- | --- | --- |
| 1 | rs38534 | TGCAGCTGCAACAACCGCTCGAGCAAAAGCTAGCAGAGAGAGAAAAGAAGGAACCGTGCATGGA | CAL, LA, LS | 20 | 45510 | 0.00042 | [TraesCS7B02G043400](https://plants.ensembl.org/Triticum_aestivum/Gene/Summary?db=core;g=TraesCS7B02G043400;tl=u73YhkNUL66hc2oV-22538386-2487360325) | 0.292 | oxidoreductase activity |  |
| 2 | rs52407 | TGCAGGTAGATGAAACGGTGGACGTGCGGATGGTGGGACGAGACGGGGCCGCCGTGCGTGTGAC | LA, LS | 2 | 66042 | 0.00002 | [TraesCS1B02G387900](https://plants.ensembl.org/Triticum_aestivum/Gene/Summary?db=core;g=TraesCS1B02G387900;tl=IdmtDrtQzU3IRuaC-22538778-2487450769) | 0.278 | DNA-binding transcription factor activity, protein dimerization activity | regulation of DNA-templated transcription |
| 3 | rs8436 | TGCAGATCCTAACACGGCACGCGTCCCAGAACCGTCTTCCCCGTCTAACGCGCCCGACCGACTC | PH | 17 | 4546 | 0.00031 | [TraesCS6B02G023900](https://plants.ensembl.org/Triticum_aestivum/Gene/Summary?db=core;g=TraesCS6B02G023900;tl=T7qTTMaYUeh5V48r-22538996-2487497933) | 0.208 | nucleotide binding, ATP binding, transferase activity, NEDD8 transferase activity | protein neddylation |
| 4 | rs26880 | TGCAGCCTCGCGTTCTCGCGGACGGTCAGAACCCGAGATCGGAAGAGCGGGATCACCGACTGCC | PH | 17 | 4546 | 0.00039 | [TraesCS6B02G022800](https://plants.ensembl.org/Triticum_aestivum/Gene/Summary?db=core;g=TraesCS6B02G022800;tl=NuPLhhfnh9Rrwq5G-22539039-2487499989) | 0.207 | chromatin binding, DNA (cytosine-5-)-methyltransferase activity, methyltransferase activity |  |
| 5 | rs29317 | TGCAGCGATTCATTCGACTTGGCGAGCAAAAACGGGGCCTTAGGCAGAGCAATGCTCACCTCGA | PH | 17 | 4546 | 0.00039 | [TraesCS6B02G023300](https://plants.ensembl.org/Triticum_aestivum/Gene/Summary?db=core;g=TraesCS6B02G023300;tl=NuPLhhfnh9Rrwq5G-22539040-2487499999) | 0.207 | double-stranded DNA binding | regulation of DNA-templated transcription |
| 6 | rs46422 | TGCAGGCAGGTGAACGACTGTACAGTCAAGCCATGGATATAATCAGGCACTCGCACGACATCGT | PH | 8 | 60303 | 0.00097 | [TraesCS3B02G444800](https://plants.ensembl.org/Triticum_aestivum/Gene/Summary?db=core;g=TraesCS3B02G444800;tl=T7qTTMaYUeh5V48r-22538991-2487492073) | 0.202 | protein binding |  |
| 7 | rs50030 | TGCAGGGATTTCATTGTCGTCACCTTCTTGGTGTTGTTGGCAGGAAGCTTAAATGCCTTCCGTG | IL1 | 19 | 133352 | 0.00028 | [TraesCS5B02G401100](https://plants.ensembl.org/Triticum_aestivum/Gene/Summary?db=core;g=TraesCS5B02G401100;tl=EVKLNmUqO23IXT4t-22538741-2487436833) | 0.195 | ADP binding | defense response |
| 8 | rs33741 | TGCAGCGTGCCTGTGGCTATACGTACTGATCGTTTCCCCGTGTTCCTCCACACGGGCAGGTTCG | IL1 | 4 | 59228 | 0.0005 | [TraesCS2A02G170200](https://plants.ensembl.org/Triticum_aestivum/Gene/Summary?db=core;g=TraesCS2A02G170200;tl=dkLuNxSwD4Sfqd4U-22538708-2487427210) | 0.192 | strictosidine synthase activity | biosynthetic process |
| 9 | rs11220 | TGCAGCAACACACCAAATAGATCATAGCCAGCTTGCTTGCACTACACGACCTAGCCCGAGATCG | IL1 | 17 | 92187 | 0.00055 | [TraesCS6B02G448800](https://plants.ensembl.org/Triticum_aestivum/Gene/Summary?db=core;g=TraesCS6B02G448800;tl=EVKLNmUqO23IXT4t-22538735-2487434380) | 0.191 | monooxygenase activity, iron ion binding, oxidoreductase activity, acting on paired donors, with incorporation or reduction of molecular oxygen, heme binding |  |
| 10 | rs58173 | TGCAGTCCACGCTCCCAGACAGCGTGGACTGGAGGGCCCGAGATCGGAAGAGCGGGATCACCGA | NFN | 6 | 22050 | 0.00021 | [TraesCS2D02G068500](https://plants.ensembl.org/Triticum_aestivum/Gene/Summary?db=core;g=TraesCS2D02G068500;tl=o5NQh9QzpPkAtsyY-22538824-2487459965) | 0.169 | cysteine-type peptidase activity | proteolysis |
| 11 | rs10133 | TGCAGATTCTAGTGCGCGCACCGCAAACCCAAGACGGCTGCTGTCCTATCACTACTCAGACGAG | NFN | 8 | 51209 | 0.00031 | [TraesCS3B02G264100](https://plants.ensembl.org/Triticum_aestivum/Gene/Summary?db=core;g=TraesCS3B02G264100;tl=b4Vm7Sf5NgdiR3FF-22538841-2487463609) | 0.167 |  | chloroplast organization |
| 12 | rs15145 | TGCAGCACGGCAAGGTTCACATCGAAACAACGAAGCAACTGAAGAAAGCTACAGGAGAGGAGAG | NFN, PH | 8 | 60303 | 0.00064 | [TraesCS3B02G447100](https://plants.ensembl.org/Triticum_aestivum/Gene/Summary?db=core;g=TraesCS3B02G447100;tl=b4Vm7Sf5NgdiR3FF-22538842-2487463684) | 0.163 | catalytic activity | nitrogen compound metabolic process, regulation of gene expression, macromolecule metabolic process, primary metabolic process |
| 13 | rs5683 | TGCAGAGATGCGAGTAGCTTTTTTTGAAAGGGAGATGCGAGTAGCTGAAGTCTGAAGAAACGCA | NFN | 8 | 51209 | 0.00071 | [TraesCS3B02G264100](https://plants.ensembl.org/Triticum_aestivum/Gene/Summary?db=core;g=TraesCS3B02G264100;tl=b4Vm7Sf5NgdiR3FF-22538843-2487463611) | 0.162 |  | chloroplast organization |
| 14 | rs50030 | TGCAGGGATTTCATTGTCGTCACCTTCTTGGTGTTGTTGGCAGGAAGCTTAAATGCCTTCCGTG | IL2 | 19 | 133352 | 0.00029 | [TraesCS2D02G042200](https://plants.ensembl.org/Triticum_aestivum/Gene/Summary?db=core;g=TraesCS2D02G042200;tl=IdmtDrtQzU3IRuaC-22538773-2487450733) | 0.148 | ADP binding | defense response |
| 15 | rs13078 | TGCAGCAATGACTCATATCAGCAGAAAACAATGATCAAGTTAGCCATGTACTACATGCAATGTG | IL2 | 14 | 70681 | 0.00073 | [TraesCS5B02G367400](https://plants.ensembl.org/Triticum_aestivum/Gene/Summary?db=core;g=TraesCS5B02G367400;tl=IdmtDrtQzU3IRuaC-22538770-2487450364) | 0.142 | serine-type endopeptidase activity | proteolysis |
| 16 | rs8943 | TGCAGATGAAGACACACTCTTAGCAGGCCAATCGCTCACCGCTGGCGACAAGCTCGTCTCGAGA | IL2 | 14 | 70681 | 0.00096 | [TraesCS5B02G367800](https://plants.ensembl.org/Triticum_aestivum/Gene/Summary?db=core;g=TraesCS5B02G367800;tl=IdmtDrtQzU3IRuaC-22538771-2487450178) | 0.141 |  | recognition of pollen |
| 17 | rs52244 | TGCAGGTACAATGTACGGCAGCCAAAATTGCGCGTTGACATGATTCTCACCCTCAGGTCGGTCG | PeD | 20 | 109456 | 0.00066 | [TraesCS7B02G434600](https://plants.ensembl.org/Triticum_aestivum/Gene/Summary?db=core;g=TraesCS7B02G434600;tl=KFulZV2k32vh6Bvx-22538957-2487478760) | 0.136 | protein binding |  |
| 18 | rs10253 | TGCAGATTGCGCACAGGCTATATATTGATTCATTGAATTTTCTTCTTCTTCTTCTTTATTTGTC | PeD | 19 | 10479 | 0.00087 | [TraesCS7A02G069700](https://plants.ensembl.org/Triticum_aestivum/Gene/Summary?db=core;g=TraesCS7A02G069700;tl=KFulZV2k32vh6Bvx-22538961-2487488215) | 0.134 | hydrolase activity, hydrolyzing O-glycosyl compounds | carbohydrate metabolic process |
| 19 | rs36483 | TGCAGCTCCCAGCCCAAAGGGGGAGCTGCTTATTTGGGCGCCTCTGCTCAAGCACCGAGATCGG | ID2 | 17 | 94461 | 0.00006 | [TraesCS6B02G453300](https://plants.ensembl.org/Triticum_aestivum/Gene/Summary?db=core;g=TraesCS6B02G453300;tl=dkLuNxSwD4Sfqd4U-22538697-2487428526) | 0.12 | protein binding |  |
| 20 | rs52123 | TGCAGGGTTTTGAGAGAAGAACTGATCCCACCTTTCAGGGAAGACCGAGATCGGAAGAGCGGGA | ID2, PeL | 1 | 114237 | 0.00099 | [TraesCS1A02G431100](https://plants.ensembl.org/Triticum_aestivum/Gene/Summary?db=core;g=TraesCS1A02G431100;tl=96jQ5IMR7LXpcA9h-22538674-2487407947) | 0.102 | protein binding |  |

Abbreviations: lodged area (LA), crop angle of inclination (CAI), lodging score index (LS), plant height (PH), number of nodes (NFN), peduncle length (PL), penultimate length (PeL), internode length 1 (IL1), internode length 2 (IL2), peduncle diameter (PD), penultimate diameter (PeD), internode diameter 1 (ID1), internode diameter 2 (ID2), days to heading (DTH), days to flowering (DTF), days to maturity (DTM), spike weight (SW), spike area (SA) and grain yield (GY).

wheat chromosomes: 1) 1A, 2) 1B, 3) 1D, 4) 2A, 5) 2B, 6) 2D, 7) 3A, 8) 3B, 9) 3D, 10) 4A, 11) 4B, 12) 4D, 13) 5A, 14) 5B, 15) 5D, 16) 6A, 17) 6B, 18) 6D, 19) 7A, 20) 7B, 21)7D.

**Supplementary Table 6** Overview on the landraces and cultivars of Iranian wheat studied

|  | |  | | Genetic background: Cultivars | | | | | | | |  | |
| --- | --- | --- | --- | --- | --- | --- | --- | --- | --- | --- | --- | --- | --- |
| No. | | Variety Name | | Introduced year | Growth Habit | | Pedigree | | | | | Selection History | |
| 1 | | 4820 | | 1951 | Spring | | - | | | | | Algeria | |
| 2 | | ADL | | 1976 | Spring | | TK/SHAHPASSAND | | | | | | |
| 3 | | AFLAK | | 2010 | Spring | | HD160/5/Tob/Cno/23854/3/Nai60//Tit/Son64/4/LR/Son64 | | | | | S-80-18 | |
| 4 | | AKBARI | | 2006 | Spring | | 1-63-31/3/12300/TOB//CNO67/SX | | | | | | |
| 5 | | AKOVA | | 1958 | Winter | | - | | | | |  | |
| 6 | | ALBORZ | | 1978 | Spring | | FN/MD//K117A/3/2*CLLF/4/SON64/KLRE/3/CNO//LR64*2/SON64 | | | | | CM2182 | |
| 7 | | ALVAND | | 1995 | Facultative | | 1-27-6275/CF 1770  or CF17170 1-22-11 | | | | | 0K | |
| 8 | | ARTA | | 2006 | Spring | | HD2206/Hork//Bul/6/CMH80A.253/2/M2A/CML//Ald*4/5/BH1146/H56.71//BH1146/3/CMH78.390/4/Seri 82/7/Hel/3*Cno79/7/2*Seri 82 | | | | | | |
| 9 | | ARVAND 1 | | 1974 | Spring | | RSH/3/MTA//KY/MAYO58 | | | | | -0IRN | |
| 10 | | ATRAK | | 1995 | Spring | | JUP/BJY'S'//URES | | | | | CM67458-4Y-1M-3Y-1M-3Y-0B | |
| 11 | | AZADI | | 1979 | Facultative | | 4820/1.32.15409//8156 | | | | | -0IRN | |
| 12 | | AZAR | | 1957 | Winter | | AZAR,LV | | | | | -0IRN | |
| 13 | | AZAR 2 | | 1997 | Winter | | KVZ/TI//MAYA/26591-1T-7M-OY-115Y-OM/3/SEFID | | | | | IMW88.1.10838-0MRGH-0MRGH-0MRGH-3MRGH-0MRGH | |
| 14 | | BAHAR | | 2007 | Spring | | HD2172/3/BB/2*7C//Y50E/3*KAL | | | | | ICW84.0008-013AP-300L-3AP-300L-0AP-0IRN | |
| 15 | | BAM | | 2006 | Spring | | VEE#5/NAC//1-66-22   or VEERY/NACOZARI-76//1-66-22 | | | | | DH-4-209-1577-F3 | |
| 16 | | BAYAT | | 1976 | Spring | | PUNJAB-76/CHENAB-70 | | | | | -0DAR | |
| 17 | | BEZOSTAYA | | 1969 | Winter | | LUTESCENS17/SKOROSPELKA2 | | | | | | |
| 18 | | BISTON | | 1980 | Spring | | 9-36/592/PIEVE or 9-36-562/PIAVE | | | | | -0IRN | |
| 19 | | CHAMRAN | | 1997 | Spring | | ND/VG9144//KAL/BB/3/YACO/4/VEE#5 | | | | | CM85836-4Y-0M-0Y-8M-0Y-0PZ | |
| 20 | | CHAMRAN-2 | | 2013 | Spring | | Attila 50Y//Attila/Bacanora | | | | | | |
| 21 | | DARAB 1 | | 1980 | Spring | | RSH/IRN 149(60-61)//C271 | | | | | -0IRN | |
| 22 | | DARAB 2 | | 1995 | Spring | | MAYA'S'/NAC | | | | | CM39424-1Y-1M-4Y-1M-1Y-1M-0Y | |
| 23 | | DARYA | | 2006 | Spring | | Sha4/Chil | | | | |  | |
| 24 | | DASTJERDI | | 1960 | Spring | | DASTJERDI | | | | | -0IRN | |
| 25 | | DAYHIM | | 1968 | Spring | | DIADEM/ITALIAI | | | | | -0K | |
| 26 | | DEZ | | 2002 | Spring | | KAUZ*2/OPATA//KAUZ | | | | | CRG737-1Y-010M-0Y-0IRN | |
| 27 | | DN-11 | | --- | --- | |  | | | | |  | |
| 28 | | FALAT | | 1990 | Spring | | KVZ/BUHO//KAL/BB | | | | | CM33027-F-15M-500Y-0M-87B-0Y | |
| 29 | | FONG | | --- | --- | | - | | | | | - | |
| 30 | | FONTANA | | --- | --- | | - | | | | | - | |
| 31 | | GAHAR | | 1996 | Spring | | ND/VG9144//KAL/BB/3/YACO'S'/4/VEE#5 | | | | | CM85836-45Y-0M-4M-0Y-0MRGH | |
| 32 | | Gascogne | | 1994 | --- | |  | | | | |  | |
| 33 | | GHODS | | 1988 | Spring | | RHS/5/WT/4/NOR10/K54*2//FN/3/PTR/6/OMID//KAL/BB | | | | | -0IRN | |
| 34 | | GOLESTAN | | 1986 | Spring | | D6301/NAI60//WRM/3/CNO*2/CHR | | | | | CM11683-0IRN | |
| 35 | | HAMOON | | 2002 | Spring | | FALAT/RSH | | | | | -0IRN | |
| 36 | | HOMA | | 2009 | Winter | | A pure Line of Sardari | | | | | 0MAR | |
| 37 | | INIA 66 | | 1969 | Spring | | LR64/SN64 | | | | | II19008-83M-100Y-100M-100Y-100C | |
| 38 | | KARAJ 1 | | 1974 | Facultative | | 200H/VFN/RSH | | | | | -0K | |
| 39 | | KARAJ 2 | | 1974 | Winter | | FA//TH/MTA/3/OMI or FA//TH/MT/3/OMID | | | | | -0K | |
| 40 | | KARAJ 3 | | 1974 | Winter | | DRC/MXP//ISWRN-297/3/NAI60 | | | | | -0K | |
| 41 | | KARIM | | 2011 | Spring | | Triticum aestivum/Sprw “s”//CA8055/3/Baconora88 | | | | | ICW92-0477-1AP-1AP-4AP-1AP-0AP | |
| 42 | | KAVEH | | 1980 | Spring | | FTA/PL | | | | | -0IRN | |
| 43 | | KAVIR | | 1997 | Spring | | Stm/3/Kal//V543/Jit716 or SHORTIM/3/KALYANSONA//V-534/JIT-716 | | | | | | |
| 44 | | KHAZAR 1 | | 1974 | Spring | | P4160//SN64/LR64 | | | | | -0G | |
| 45 | | KOOHDASHT | | 2002 | Spring | | BB/RON//CNO67/TOTA/3/JAR | | | | | TR810200-29R-1R-6R-0R-0IRN | |
| 46 | | MAHDAVI | | 1995 | Spring | | TI/PCH/5/MT48/3/WTE*3/NAR59/TOTA63/4/MUS | | | | | ICW84 | |
| 47 | | MAROON | | 1991 | Spring | | AVD/PCHU/5/N10/BR21.1C//KT54B/3/NAR59/1093/4/7C | | | | | | |
| 48 | | MARVDASHT | | 1999 | Spring | | HD2172/BLOUDAN//AZADI | | | | | -0SHZ | |
| 49 | | MIHAN | | 2010 | Winter | | Barkat/90Zhong87 | | | | | | |
| 50 | | MOGHAN 1 | | 1974 | Spring | | LR/N10B//3*ANE | | | | | II8739-4R-1M-1R-0IRN | |
| 51 | | MOGHAN 2 | | 1974 | Spring | | LR64A/HUAR | | | | | II15929-1M-4R-2M-0IND-0IRN | |
| 52 | | MOGHAN 3 | | 2006 | Spring | | Luan/3/V763.23/V879.c8//Pvn/4/Picus/5/Opata | | | | | N-80-6 | |
| 53 | | MORVARID | | 2009 | Spring | | MILAN/SHANGHAI-7 | | | | | CM97550-0M-2Y-030H-3Y-3Y-0Y-2M-010Y-0FUS-3FUS-1FUS-0Y-2SJ-0Y | |
| 54 | | MV-17 | | 1993 | Winter | | - | | | | | Marton Vassar, Hungry | |
| 55 | | NAVID 1990 | | 1990 | Facultative | | HYS/7C | | | | |  | |
| 56 | | NAZ | | 1978 | Spring | | II12300//LR64A/8156/3/NOR | | | | | II30842-31R-2M-2Y-0M-0IRN | |
| 57 | | NEISHABOUR | | 2006 | Spring | | 1-63-31/3/12300/TOB//CNO67/SX | | | | | | |
| 58 | | NICKNEJAD | | 1995 | Spring | | F134-71/CROW'S' | | | | | SWM11147-1AP-2AP-4AP-1AP-0AP-0MRGH | |
| 59 | | OFOGH | | 2012 | Spring | | GF-gy54/Attila | | | | | Cross made in Iran at SPII in 1995. Cross #1-14437 | |
| 60 | | OHADI | | 2010 | Winter | | Selection in landraces | | | | | | |
| 61 | | PANJAMO 62 | | 1968 | Spring | |  | | | | |  | |
| 62 | | PARSI | | 2009 | Spring | | Dove"S"/Buc"S"//2*Darab1 or DOVE(SIB)/(SIB)BUCKBUCK(M-84-17)//2*DARAB | | | | | | |
| 63 | | PISHGAM | | 2008 | Facultative | | Bkt/90Zhong87 | | | | | 0K | |
| 64 | | PISHTAZ | | 2002 | Spring | | ALVAND//ALDAN/IAS 58 | | | | | -0IRN | |
| 65 | | QABOOS | | 2014 | Spring | | Dryland Agricultural Research Institute | | | | | | |
| 66 | | RASHID | | 1968 | Facultative | | N.P.7881/AZAR 2/588 | | | | | -01IRN | |
| 67 | | RAYHANI | | 1942 | Spring | | RAYHANI | | | | | 0IRN | |
| 68 | | RIJAW | | 2011 | Facultative | | PATO/CAL/3/7C//BB/CNO/5/CAL//CNO/SN64/4/CNO//BAD/CHR/3/KL../6/SABALAN | | | | | IWWIP | |
| 69 | | ROSHAN | | 1960 | Spring | | Landrace | | | | | -0IRN | |
| 70 | | SABALAN | | 1980 | Spring | | (908//FN/A12)1-32-4382 | | | | | -0IRN | |
| 71 | | SEPAHAN | | 2006 | Spring | | AZADI/5/L2453/1347/4/KAL//BB/KAL/3/Y50E/3*KAL | | | | | -0ISF | |
| 72 | | SHAHI | | 1967 | Winter | | SHAHI | | | | | 0IRN | |
| 73 | | SHAHPASSAND | | 1942 | Winter | | SHAHPASSAND | | | | | 0IRN | |
| 74 | | SHAHRYAR | | 2002 | Winter | | KVZ/TI//MAYA/26591-1T-7M-OY-115Y-OM/3/1-44-21863/4/ANZA/3/PI/NAR59//HYS or KAVKAZ/TANORI-71/3/MAYA-74(SIB)//BLUEBIRD/INIA/4/KARAJ-2/5/ANZA/3/PITIC-62/NDR//HYSLOP | | | | | -0IRN | |
| 75 | | SHANGHAI #7 | | --- | Spring | |  | | | | |  | |
| 76 | | SHIRAZ | | 2002 | Spring | | GV/D6301//ALD/3/AZADI or GAVILAN,MEX/D-630//(SIB)ALONDRA/3/AZADI  or ALVAND//ALDAN/IAS-58 | | | | | -0IRN | |
| 77 | | SHIROODI | | 1997 | Spring | | ND/VG9144//KAL/BB/3/YACO/4/VEE#5 | | | | | CM85836-4Y-0M-0Y-8M-0Y-0PZ-0IRN | |
| 78 | | SIOSSON | | 1994 | Spring | | - | | | | |  | |
| 79 | | SIRVAN | | 2012 | Spring | | PRL/2*PASTOR | | | | | CGSS97Y00034M-099TOPB-027Y-099M-099Y-099M-27Y-0B | |
| 80 | | SISTAN | | 2006 | Spring | | Bank"s"/Veery"s" | | | | | | |
| 81 | | SIVAND | | 2009 | Spring | | Kauz"S"/Azd or KAUZ(SIB)/(AZD)AZADI | | | | | M-84-18 | |
| 82 | | TAJAN | | 1995 | Spring | | BOW/NKT | | | | | CM67428-6M-1Y-05M-3Y-0B-0K | |
| 83 | | TAK-AB | | 2013 | Spring | | Manning/Sdv1//Dogu88 | | | | | 0YC-0YC-0YC-12YC-0YC | |
| 84 | | TOBARI 66 | | 1969 | Spring | | - | | | | |  | |
| 85 | | TOUS | | 2002 | Facultative | | SPN/MCD//CAM/3/NZR or  SPN/MCD//CAMA/3/NZT | | | | | SWM777627'-17H-4H-1H-0H | |
| 86 | | UROUM | | 2009 | Winter | | Alvand//Ns732/Her | | | | | | |
| 87 | | VEE/NAC | | 1997 | Spring | | Veery/Nacozari or Veery#5/NacozariF76 | | | | | CM67575 | |
| 88 | | ZAGROS | | 1996 | Spring | | TAN'S'/VEE'S'//OPATA | | | | | CM82781-030TOPM-14Y-025H-05H-0SY-05RB-0H-0MRGH | |
| 89 | | ZARE | | 2010 | Facultative | | 130L1.11//F35.70/MO73/4/YMH/TOB//MCD/3/LIRA | | | | | CIT925080-0SE-0YC-7YC-0YC-1YC-0YC-3YC-0YC | |
| 90 | | ZARRIN | | 1995 | Spring | | NAI60/HVII//BUC/3/F59.71/GHK | | | | | SWO791095 | |
| Genetic background: Landraces | | | | | | |  |  |  |  | |  |  |
| No. | | Region of origin (Province) | | | USDA_PI_NO | |  | No. | Region of origin (Province) | USDA_PI_NO | |  |  |
| 91 | | Azarbayjan-Gharbi | | | 620903 | |  | 195 | Gazvin | 624985 | |  |  |
| 92 | | Hamadan | | | 621420 | |  | 196 | Gazvin | 624990 | |  |  |
| 93 | | Hamadan | | | 621421 | |  | 197 | Markazi | 625047 | |  |  |
| 94 | | Bakhtaran | | | 621492 | |  | 198 | Markazi | 625080 | |  |  |
| 95 | | Hamadan | | | 621565 | |  | 199 | Markazi | 625081 | |  |  |
| 96 | | Kordestan | | | 621619 | |  | 200 | Markazi | 625123 | |  |  |
| 97 | | Tehran | | | 621650 | |  | 201 | Markazi | 625127 | |  |  |
| 98 | | Tehran | | | 621668 | |  | 202 | Markazi | 625139 | |  |  |
| 99 | | Tehran | | | 621669 | |  | 203 | Mazandaran | 625263 | |  |  |
| 100 | | Gazvin | | | 621704 | |  | 204 | Gilan | 625281 | |  |  |
| 101 | | Gazvin | | | 621706 | |  | 205 | Mazandaran | 625362 | |  |  |
| 102 | | Gazvin | | | 621712 | |  | 206 | Khorasan | 625433 | |  |  |
| 103 | | Gazvin | | | 621716 | |  | 207 | Khorasan | 625661 | |  |  |
| 104 | | Gazvin | | | 621717 | |  | 208 | Khorasan | 625810 | |  |  |
| 105 | | Gazvin | | | 621735 | |  | 209 | Kerman | 626156 | |  |  |
| 106 | | Gazvin | | | 621736 | |  | 210 | Kerman | 626158 | |  |  |
| 107 | | Markazi | | | 621869 | |  | 211 | Kerman | 626215 | |  |  |
| 108 | | Markazi | | | 621908 | |  | 212 | Sistan-Balouchestan | 626223 | |  |  |
| 109 | | Zanjan | | | 622063 | |  | 213 | Sistan-Balouchestan | 626226 | |  |  |
| 110 | | Mazandaran | | | 622084 | |  | 214 | Sistan-Balouchestan | 626234 | |  |  |
| 111 | | Gilan | | | 622098 | |  | 215 | Sistan-Balouchestan | 626260 | |  |  |
| 112 | | Gilan | | | 622099 | |  | 216 | Sistan-Balouchestan | 626261 | |  |  |
| 113 | | Gilan | | | 622105 | |  | 217 | Esfahan | 626358 | |  |  |
| 114 | | Mazandaran | | | 622247 | |  | 218 | Esfahan | 626360 | |  |  |
| 115 | | Mazandaran | | | 622264 | |  | 219 | Esfahan | 626565 | |  |  |
| 116 | | Mazandaran | | | 622272 | |  | 220 | Esfahan | 626566 | |  |  |
| 117 | | Khorasan | | | 622311 | |  | 221 | Esfahan | 626573 | |  |  |
| 118 | | Khorasan | | | 622379 | |  | 222 | Ilam | 626699 | |  |  |
| 119 | | Esfahan | | | 622894 | |  | 223 | Hamadan | 626706 | |  |  |
| 120 | | Esfahan | | | 623008 | |  | 224 | Khorasan | 626736 | |  |  |
| 121 | | Esfahan | | | 623069 | |  | 225 | Yazd | 626747 | |  |  |
| 122 | | Bakhtaran | | | 623090 | |  | 226 | Yazd | 626764 | |  |  |
| 123 | | Khorasan | | | 623091 | |  | 227 | Khorasan | 626776 | |  |  |
| 124 | | Yazd | | | 623109 | |  | 228 | Esfahan | 626814 | |  |  |
| 125 | | Fars | | | 623123 | |  | 229 | Esfahan | 626825 | |  |  |
| 126 | | Fars | | | 623125 | |  | 230 | Yazd | 626846 | |  |  |
| 127 | | Fars | | | 623127 | |  | 231 | Markazi | 626855 | |  |  |
| 128 | | Azarbayjan-Gharbi | | | 623136 | |  | 232 | Fars | 626872 | |  |  |
| 129 | | Fars | | | 623139 | |  | 233 | Azarbayjan-Shargi | 626881 | |  |  |
| 130 | | Azarbayjan-Gharbi | | | 623161 | |  | 234 | Fars | 626883 | |  |  |
| 131 | | Azarbayjan-Gharbi | | | 623162 | |  | 235 | Azarbayjan-Shargi | 626895 | |  |  |
| 132 | | Gilan | | | 623169 | |  | 236 | Azarbayjan-Shargi | 626904 | |  |  |
| 133 | | Khorasan | | | 623176 | |  | 237 | Kerman | 626908 | |  |  |
| 134 | | Azarbayjan-Gharbi | | | 623266 | |  | 238 | Gilan | 626923 | |  |  |
| 135 | | Bakhtaran | | | 623274 | |  | 239 | Gilan | 626924 | |  |  |
| 136 | | Hamadan | | | 623291 | |  | 240 | Hormozgan | 626932 | |  |  |
| 137 | | Yazd | | | 623318 | |  | 241 | Hormozgan | 626933 | |  |  |
| 138 | | Fars | | | 623338 | |  | 242 | Kerman | 626943 | |  |  |
| 139 | | Bakhtaran | | | 623344 | |  | 243 | Azarbayjan-Gharbi | 626958 | |  |  |
| 140 | | Kordestan | | | 623345 | |  | 244 | Esfahan | 626978 | |  |  |
| 141 | | Kerman | | | 623377 | |  | 245 | Khouzestan | 627036 | |  |  |
| 142 | | Kerman | | | 623379 | |  | 246 | Khouzestan | 627038 | |  |  |
| 143 | | Kerman | | | 623382 | |  | 247 | Azarbayjan-Gharbi | 627043 | |  |  |
| 144 | | Sistan-Balouchestan | | | 623417 | |  | 248 | Gilan | 627054 | |  |  |
| 145 | | Azarbayjan-Shargi | | | 623421 | |  | 249 | Zanjan | 627055 | |  |  |
| 146 | | Azarbayjan-Shargi | | | 623428 | |  | 250 | Gilan | 627057 | |  |  |
| 147 | | Ilam | | | 623473 | |  | 251 | Markazi | 627061 | |  |  |
| 148 | | Ilam | | | 623475 | |  | 252 | Kerman | 627066 | |  |  |
| 149 | | Ilam | | | 623503 | |  | 253 | Zanjan | 627072 | |  |  |
| 150 | | Bakhtaran | | | 623506 | |  | 254 | Khouzestan | 627099 | |  |  |
| 151 | | Bakhtaran | | | 623507 | |  | 255 | Zanjan | 627102 | |  |  |
| 152 | | Bakhtaran | | | 623508 | |  | 256 | Mazandaran | 627103 | |  |  |
| 153 | | Azarbayjan-Gharbi | | | 623510 | |  | 257 | Khorasan | 627189 | |  |  |
| 154 | | Bakhtaran | | | 623905 | |  | 258 | Khorasan | 627236 | |  |  |
| 155 | | Bakhtaran | | | 623908 | |  | 259 | Yazd | 627299 | |  |  |
| 156 | | Bakhtaran | | | 623909 | |  | 260 | Hormozgan | 627356 | |  |  |
| 157 | | Bakhtaran | | | 623953 | |  | 261 | Markazi | 627359 | |  |  |
| 158 | | Hamadan | | | 623980 | |  | 262 | Kerman | 627360 | |  |  |
| 159 | | Hamadan | | | 624215 | |  | 263 | Bakhtaran | 627385 | |  |  |
| 160 | | Ilam | | | 624240 | |  | 264 | Zanjan | 627399 | |  |  |
| 161 | | Ilam | | | 624251 | |  | 265 | Azarbayjan-Shargi | 627410 | |  |  |
| 162 | | Kordestan | | | 624315 | |  | 266 | Bakhtaran | 627414 | |  |  |
| 163 | | Bakhtaran | | | 624378 | |  | 267 | Bakhtaran | 627416 | |  |  |
| 164 | | Bakhtaran | | | 624381 | |  | 268 | Bakhtaran | 627417 | |  |  |
| 165 | | Hamadan | | | 624576 | |  | 269 | Hamadan | 627423 | |  |  |
| 166 | | Hamadan | | | 624580 | |  | 270 | Khorasan | 627460 | |  |  |
| 167 | | Hamadan | | | 624582 | |  | 271 | Yazd | 627484 | |  |  |
| 168 | | Hamadan | | | 624585 | |  | 272 | Azarbayjan-Shargi | 627551 | |  |  |
| 169 | | Hamadan | | | 624596 | |  | 273 | Kordestan | 627587 | |  |  |
| 170 | | Bakhtaran | | | 624804 | |  | 274 | Esfahan | 627616 | |  |  |
| 171 | | Bakhtaran | | | 624805 | |  | 275 | Esfahan | 627688 | |  |  |
| 172 | | Ilam | | | 624818 | |  | 276 | Yazd | 627723 | |  |  |
| 173 | | Ilam | | | 624837 | |  | 277 | Azarbayjan-Shargi | 627760 | |  |  |
| 174 | | Ilam | | | 624838 | |  | 278 | Azarbayjan-Shargi | 627787 | |  |  |
| 175 | | Ilam | | | 624846 | |  | 279 | Kerman | 627842 | |  |  |
| 176 | | Ilam | | | 624849 | |  | 280 | Sistan-Balouchestan | 627845 | |  |  |
| 177 | | Ilam | | | 624861 | |  | 281 | Sistan-Balouchestan | 627849 | |  |  |
| 178 | | Ilam | | | 624863 | |  | 282 | Sistan-Balouchestan | 627852 | |  |  |
| 179 | | Ilam | | | 624864 | |  | 283 | Sistan-Balouchestan | 627853 | |  |  |
| 180 | | Kordestan | | | 624894 | |  | 284 | Mazandaran | 627856 | |  |  |
| 181 | | Kordestan | | | 624900 | |  | 285 | Zanjan | 627873 | |  |  |
| 182 | | Kordestan | | | 624901 | |  | 286 | Azarbayjan-Shargi | 627881 | |  |  |
| 183 | | Hamadan | | | 624910 | |  | 287 | Azarbayjan-Shargi | 627883 | |  |  |
| 184 | | Hamadan | | | 624911 | |  | 288 | Mazandaran | 627905 | |  |  |
| 185 | | Hamadan | | | 624925 | |  | 289 | Markazi | 627908 | |  |  |
| 186 | | Tehran | | | 624939 | |  | 290 | Markazi | 627948 | |  |  |
| 187 | | Tehran | | | 624941 | |  | 291 | Hamadan | 627963 | |  |  |
| 188 | | Tehran | | | 624944 | |  | 292 | Zanjan | 627987 | |  |  |
| 189 | | Tehran | | | 624946 | |  | 293 | Bakhtaran | 627990 | |  |  |
| 190 | | Tehran | | | 624947 | |  | 294 | Bakhtaran | 628012 | |  |  |
| 191 | | Tehran | | | 624956 | |  | 295 | Mazandaran | 628084 | |  |  |
| 192 | | Tehran | | | 624963 | |  | 296 | Markazi | 628088 | |  |  |
| 193 | | Gazvin | | | 624980 | |  | 297 | Esfahan | 628114 | |  |  |
| 194 | | Gazvin | | | 624983 | |  | 298 | Ilam | 628189 | |  |  |

**Supplementary Table 7** Pattern of total monthly precipitation and irrigation for the 2018-19 and 2019-20 cropping seasons.

| **Year** | **Month** | **Max Temperature °C** | **Min Temperature °C** | **Average Temperature °C** | **Average rainfall, mm** | **Average relative humidity** | **Sunny hours** | **Evaporation, mm** |
| --- | --- | --- | --- | --- | --- | --- | --- | --- |
| 2018-2019 | November | 14.561 | 4.104 | 10.900 | 0.93 | 45.810 | 6.893 | 3.068 |
|  | December | 9.242 | -0.119 | 4.671 | 41.11 | 60.134 | 5.065 | 0.000 |
|  | January | 8.406 | -0.613 | 3.668 | 15.04 | 57.750 | 6.652 | 0.000 |
|  | February | 7.871 | -2.254 | 2.536 | 27.99 | 61.429 | 6.868 | 0.000 |
|  | March | 14.216 | 4.623 | 9.271 | 38.44 | 56.847 | 5.942 | 0.179 |
|  | April | 21.093 | 9.563 | 15.110 | 46.65 | 49.954 | 6.587 | 4.497 |
|  | May | 29.229 | 14.261 | 21.935 | 22.01 | 38.722 | 10.435 | 7.377 |
|  | June | 34.159 | 17.597 | 26.083 | 0.00 | 32.304 | 12.763 | 11.676 |
| 2019-2020 | November | 17.080 | 6.383 | 11.520 | 0.63 | 43.479 | 6.960 | 3.189 |
|  | December | 12.303 | 1.652 | 6.671 | 4.71 | 50.419 | 7.226 | 0.000 |
|  | January | 9.077 | -0.055 | 4.052 | 19.84 | 54.476 | 6.526 | 0.000 |
|  | February | 10.739 | 2.039 | 6.464 | 31.73 | 64.755 | 5.829 | 0.000 |
|  | March | 20.558 | 8.377 | 14.652 | 14.11 | 38.952 | 7.303 | 0.000 |
|  | April | 19.983 | 7.793 | 13.633 | 45.81 | 51.413 | 7.563 | 6.714 |
|  | May | 25.513 | 12.061 | 18.432 | 57.07 | 54.907 | 8.287 | 6.161 |
|  | June | 33.807 | 17.347 | 25.583 | 7.23 | 37.492 | 11.100 | 11.143 |
|  |  |  |  |  |  |  |  |  |
|  | **Month** | **ET_0_ (mm)** | **K_C_** | **ET_C_ (mm)** | **Required water per ha (m^3^ /ha)** | **Required water for 1377 m^2^ (m^3^)** | **Water discharge (m^3^ /h)** | **Period of irrigation (h)** |
| 2018-2019  and 2019-2020 | March | 40 | 0.92 | 36.8 | 368 | 50.69 | 10.8 | 4.69 |
|  | April | 40 | 1.33 | 53.2 | 532 | 73.28 | 10.8 | 6.79 |
|  | May | 40 | 1.15 | 46 | 46 | 63.37 | 10.8 | 5.87 |
|  | June | 40 | 0.58 | 23.2 | 232 | 31.96 | 10.8 | 2.96 |

| **A**  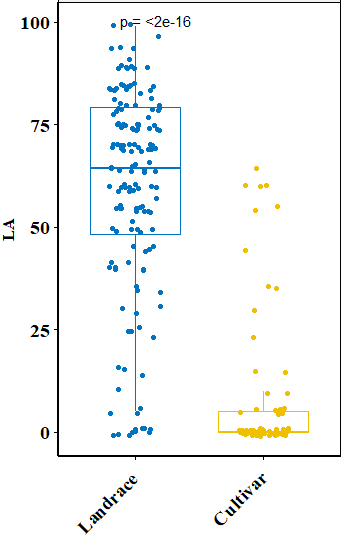 | **B**  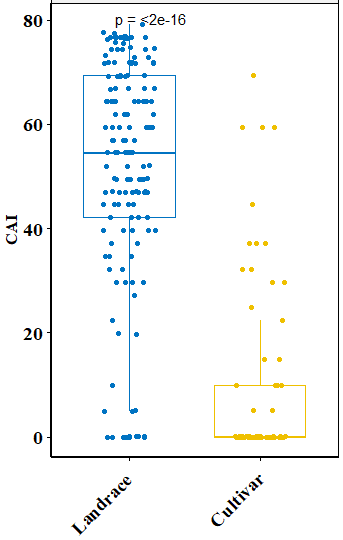 | **C**  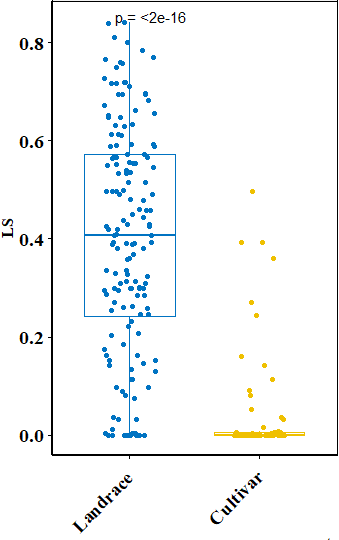 | **D**  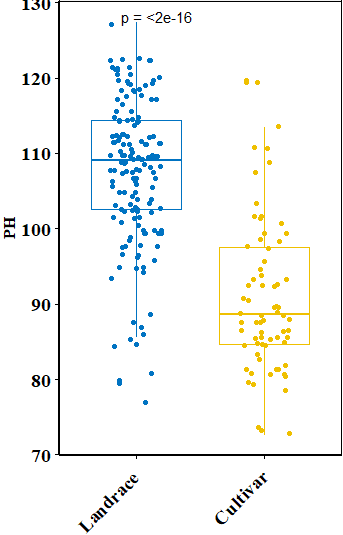 | **E**  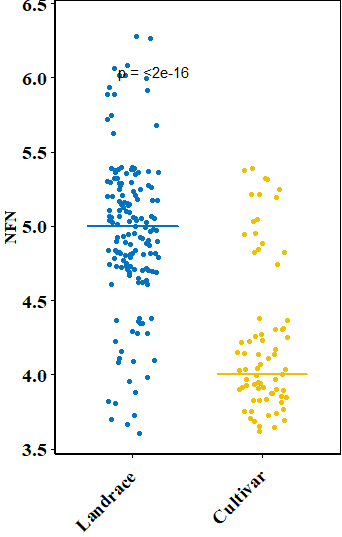 |
| --- | --- | --- | --- | --- |
| **F**  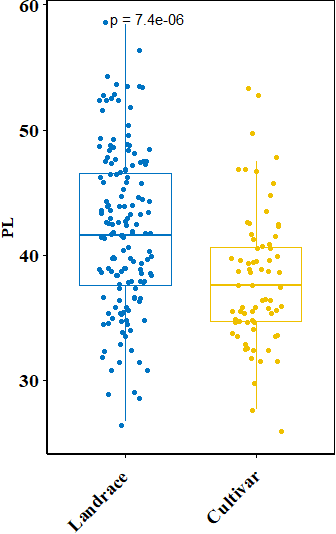 | **G**  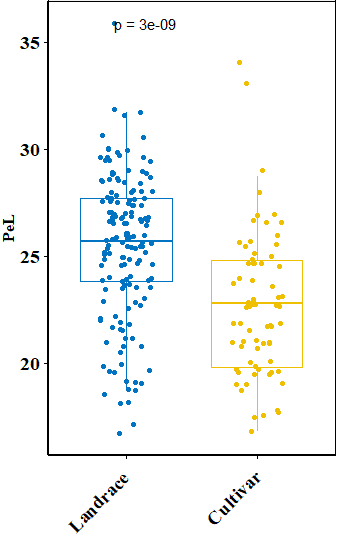 | **H**  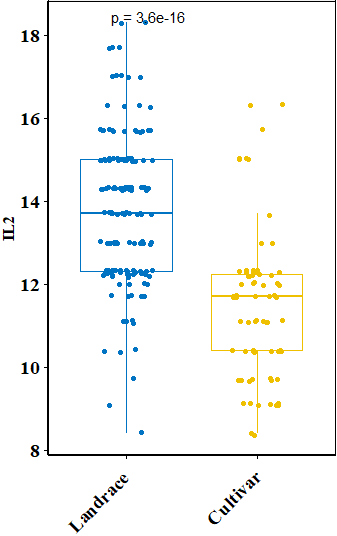 | **I**  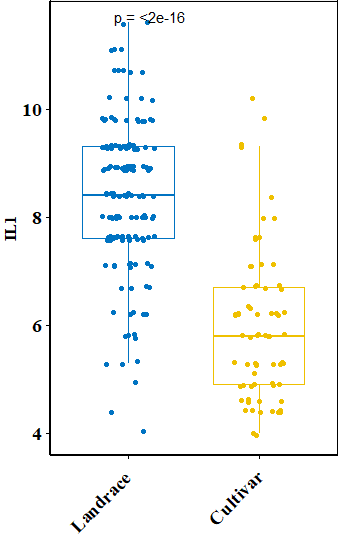 | **J**  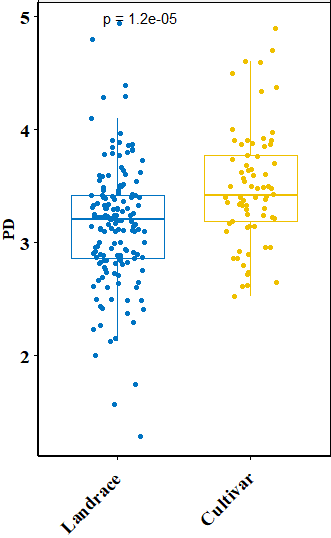 |
| **K**  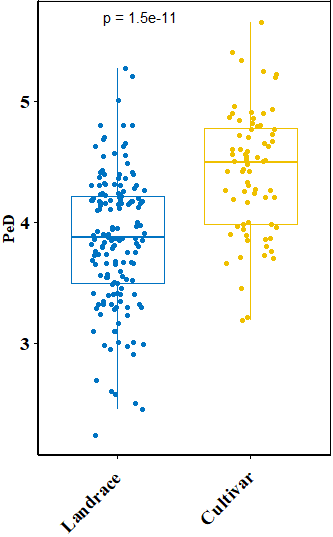 | **L**  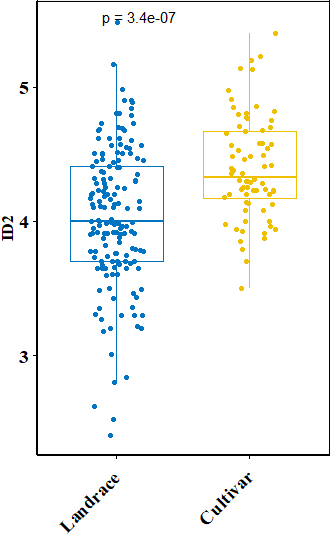 | **M**  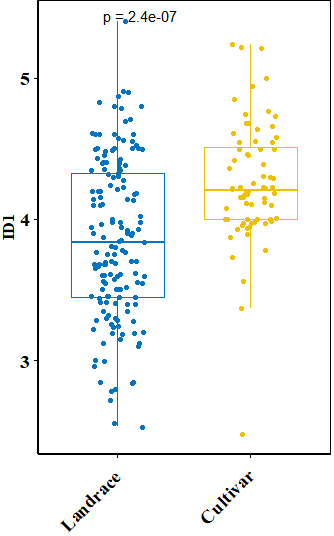 | **N**  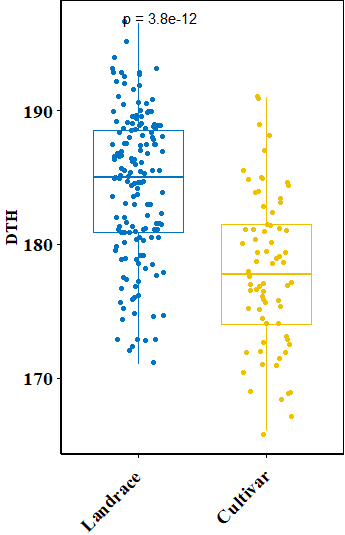 | **O**  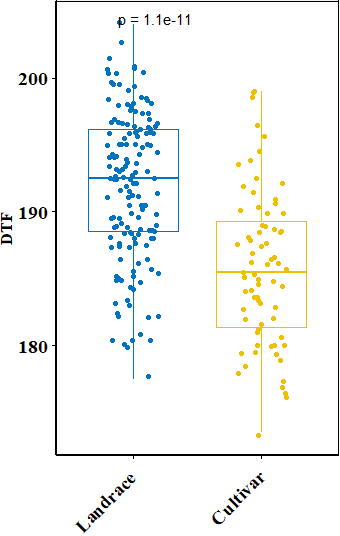 |
| **P**  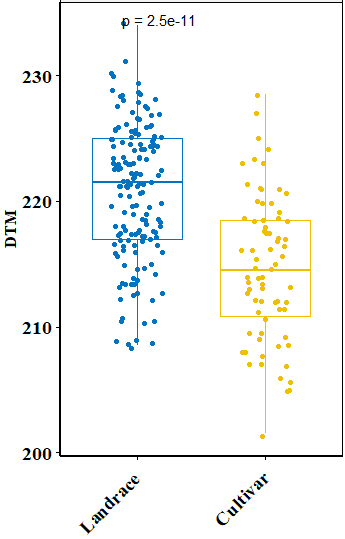 | **Q**  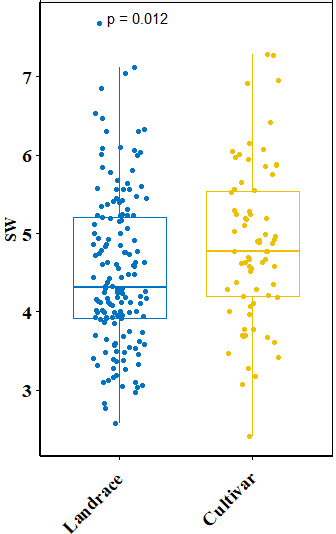 | 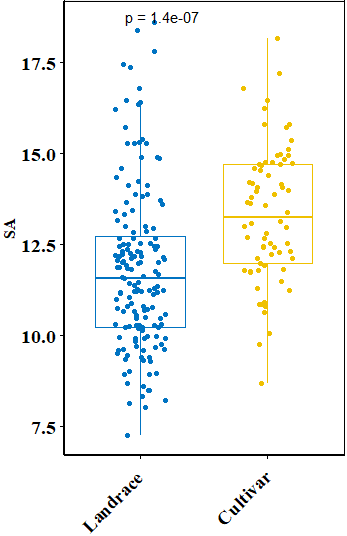**R** | **S**  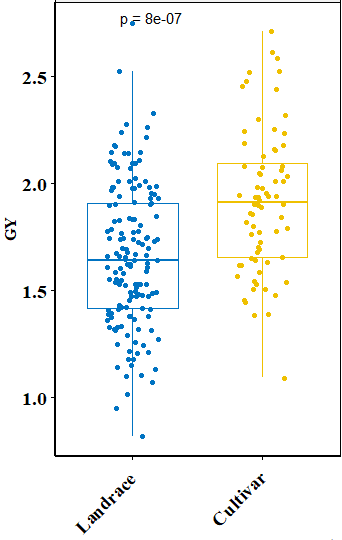 |  |

**Supplementary Fig. 1** Box-plot presentation of the distribution for 19 lodging-related traits in Iranian wheat cultivars and landraces.

Abbreviations: Lodged area (LA), crop angle of inclination (CAI), lodging score index (LS), plant height (PH), number of nodes (NFN), peduncle length (PL), penultimate length (PeL), internode length 1 (IL1), internode length 2 (IL2), peduncle diameter (PD), penultimate diameter (PeD), internode diameter 1 (ID1), internode diameter 2 (ID2), days to heading (DTH), days to flowering (DTF), days to maturity (DTM), spike weight (SW), spike area (SA) and grain yield (GY).

| A | B |
| --- | --- |
|  |  |

**Supplementary Fig. 2** Number of imputed SNPs used in different chromosomes of the wheat genomes (A), number of imputed SNPs used in wheat genomes (B).

| DTF (year1) |  |
| --- | --- |
| 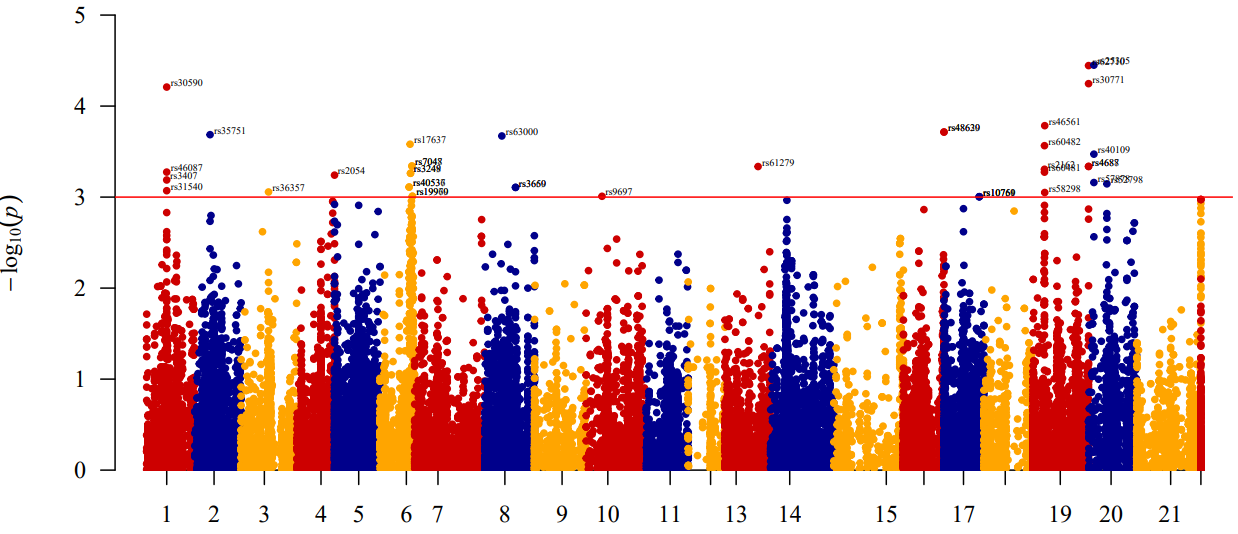 | 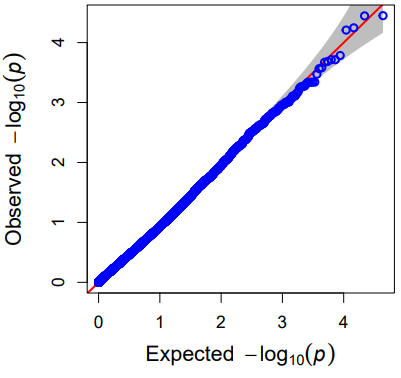 |
| DTH (year1) |  |
| 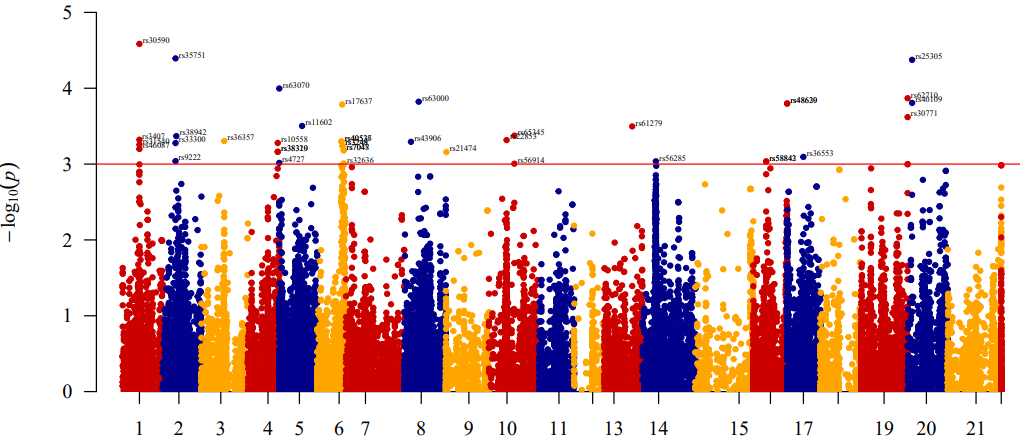 | 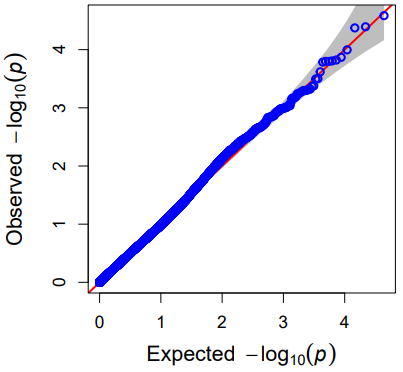 |
| DTM (year1) |  |
| 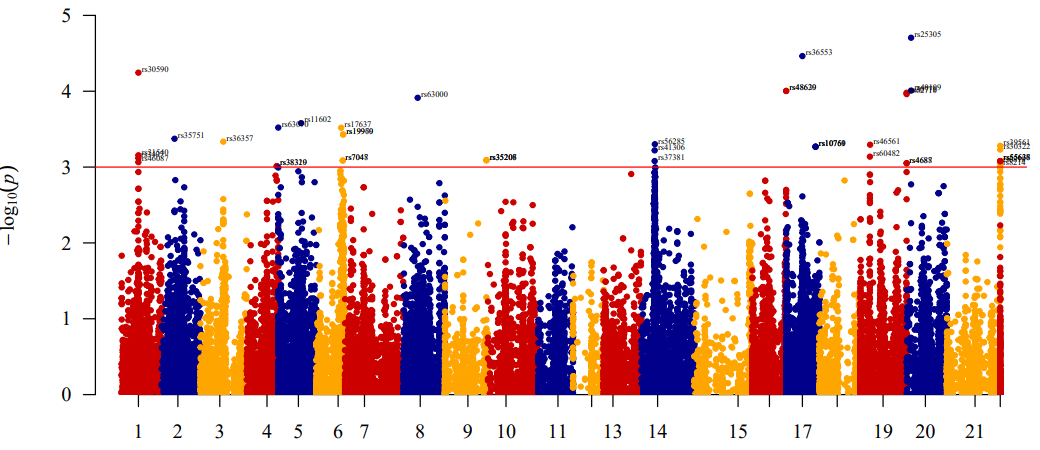 | 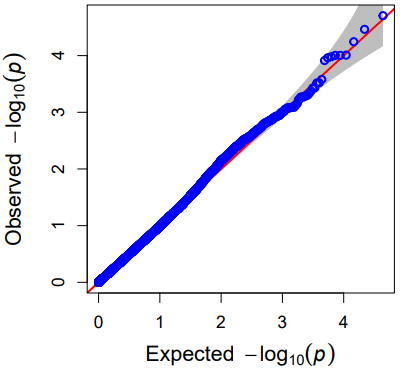 |
| GY (year1) |  |
| 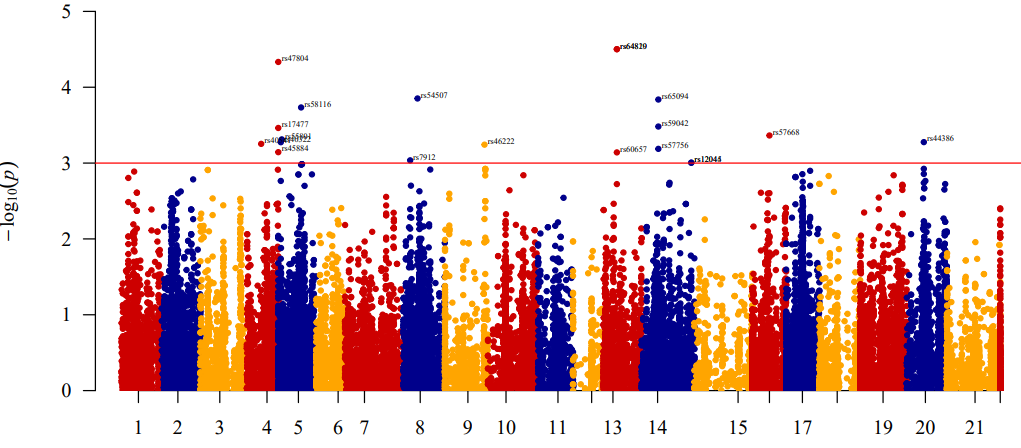 | 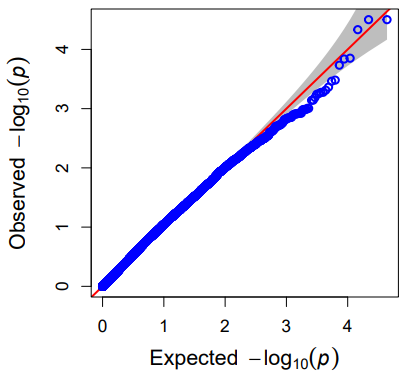 |
| ID1 (year1) |  |
| 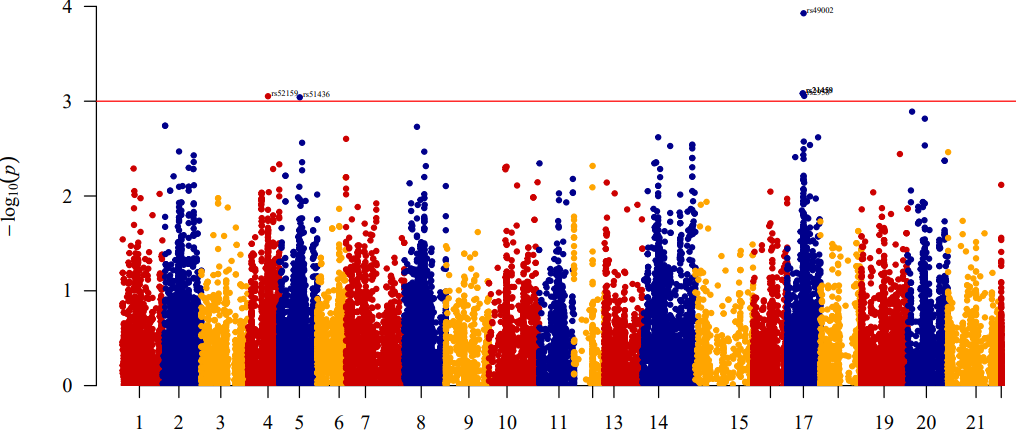 | 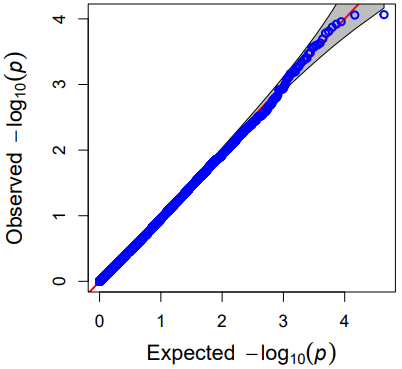 |
| ID2 (year1) |  |
| 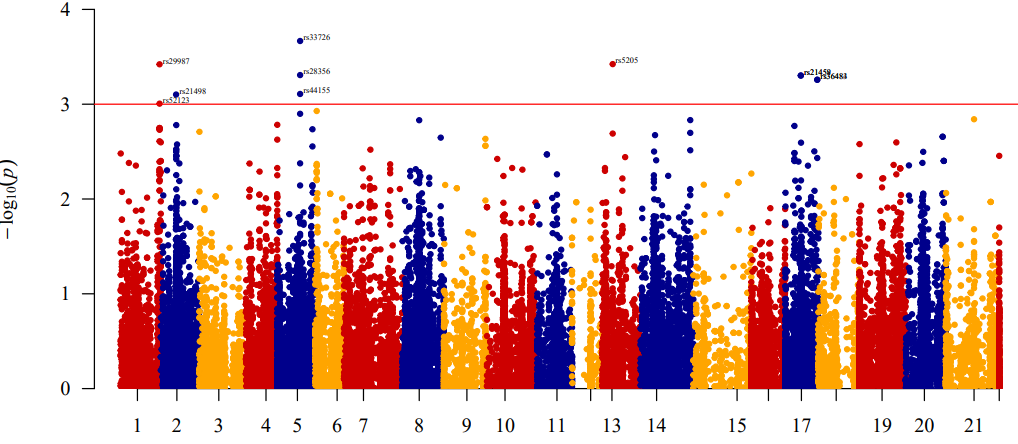 | 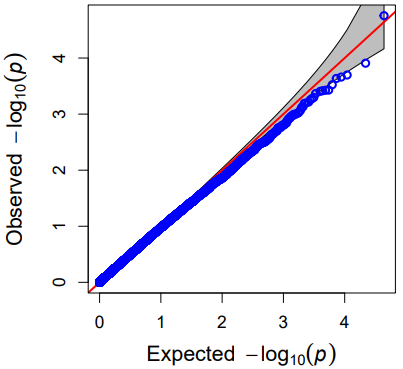 |
| IL1 (year1) |  |
| 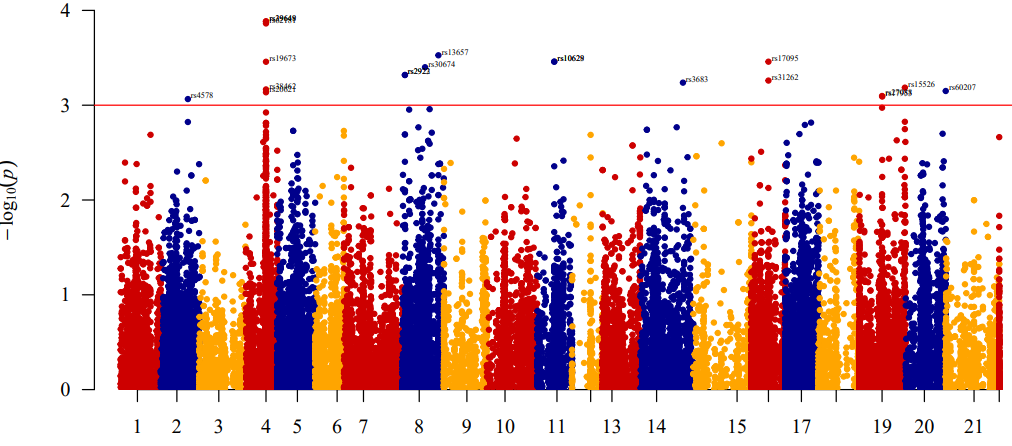 | 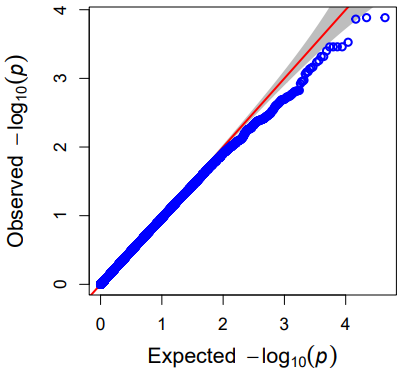 |
| IL2 (year1) |  |
| 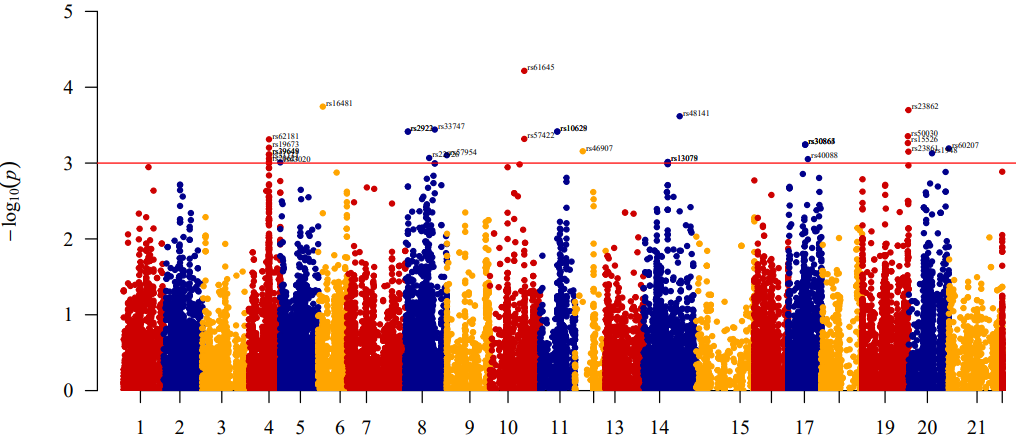 | 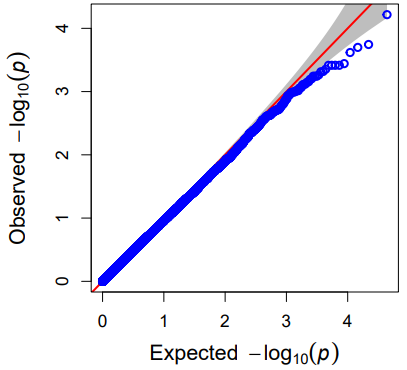 |
| NFN (year1) |  |
| 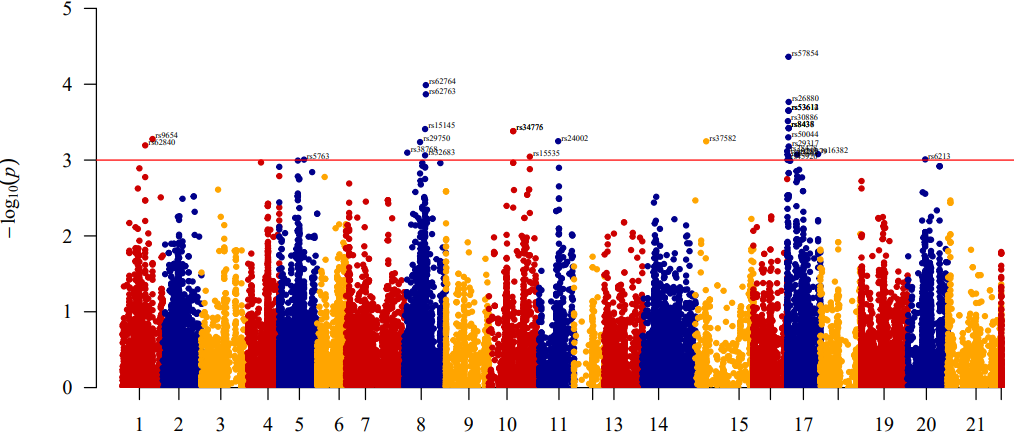 | 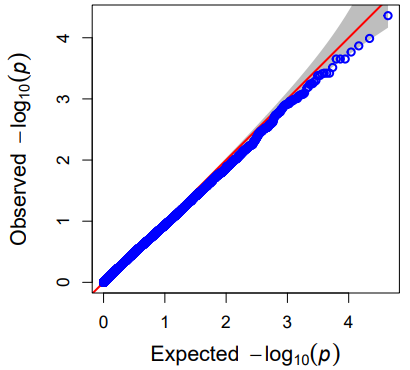 |
| PD (year1) |  |
| 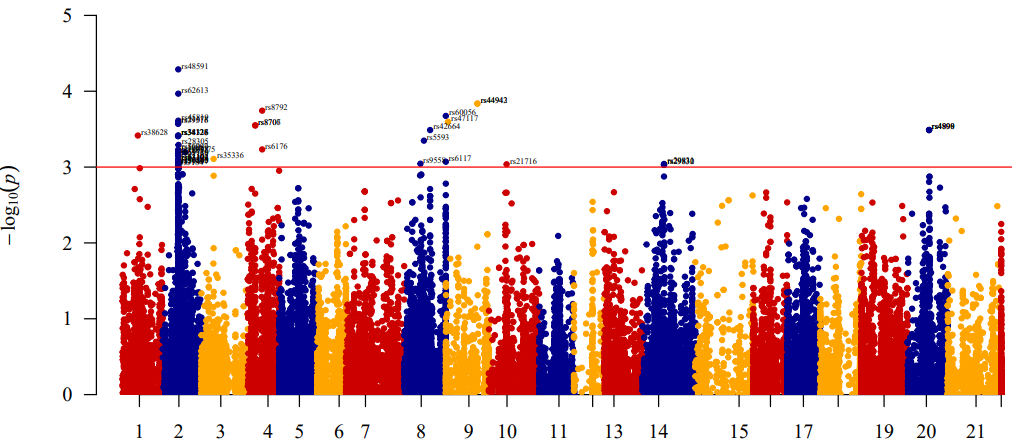 | 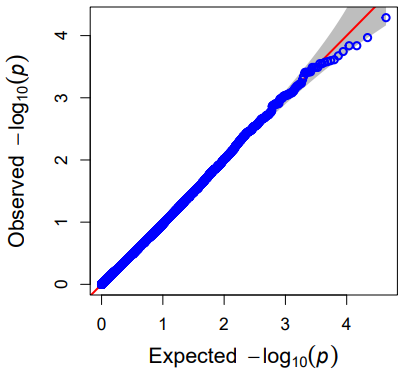 |
| PeD (year1) |  |
| 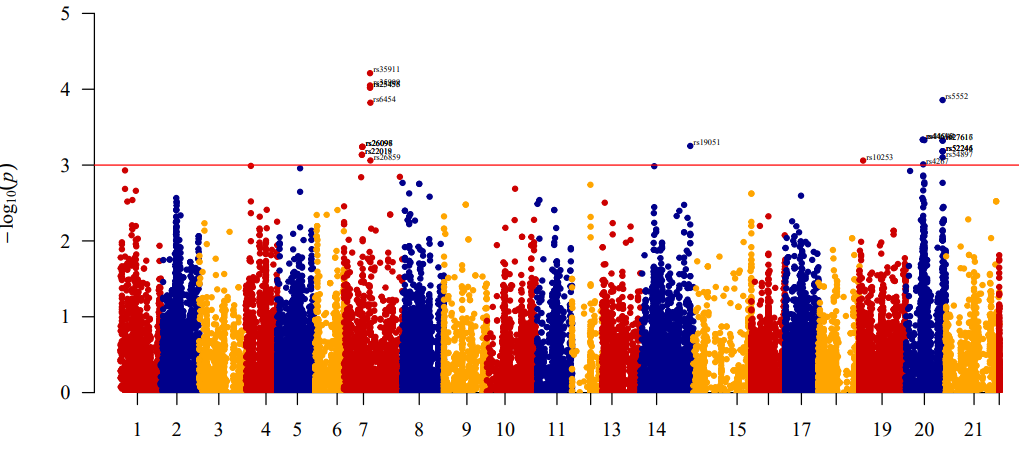 | 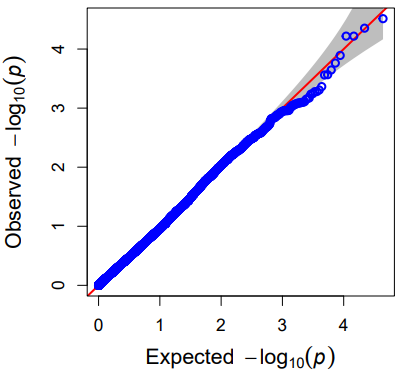 |
| PeL (year1) |  |
| 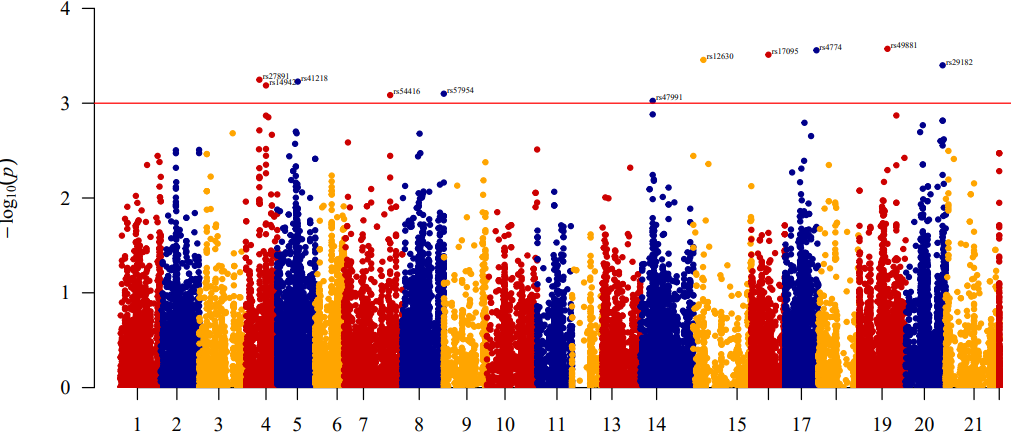 | 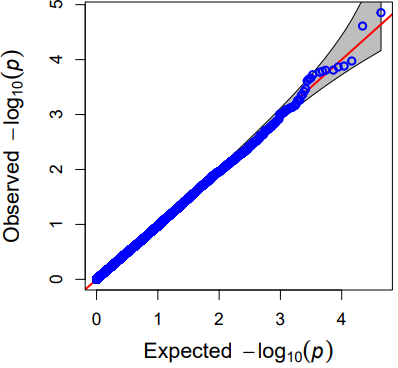 |
| PL (year1) |  |
| 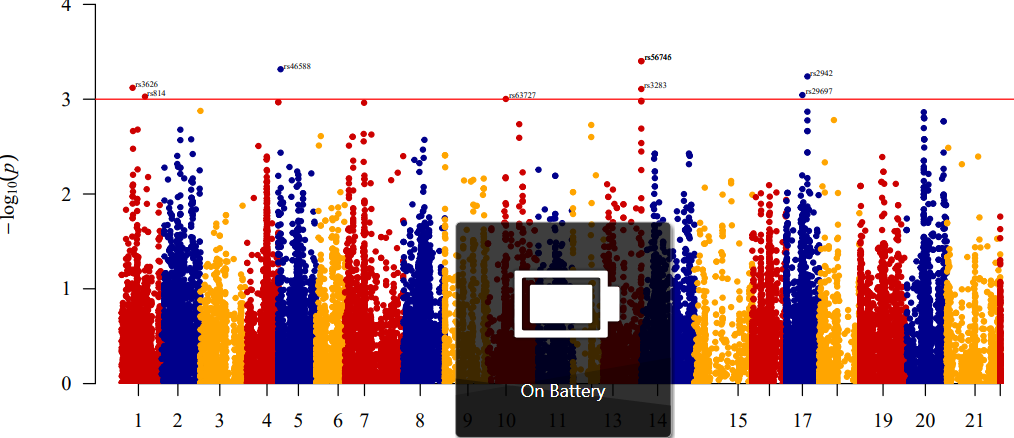 | 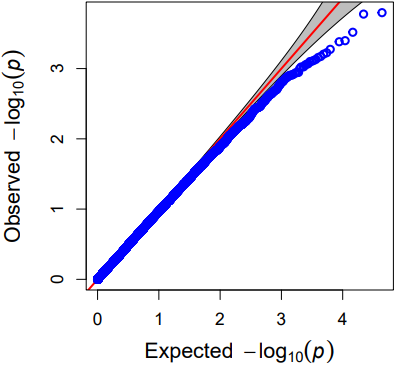 |
| SA (year1) |  |
| 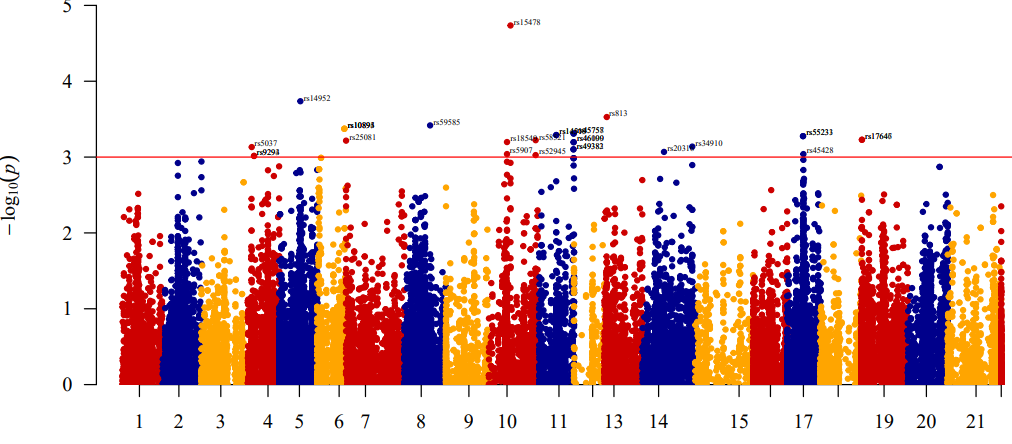 | 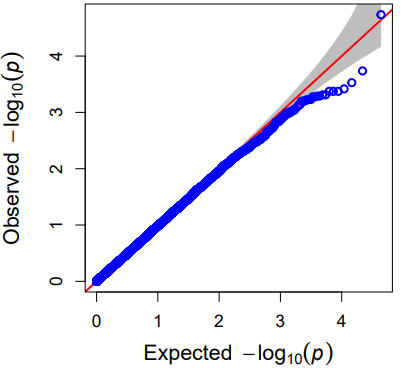 |
| SW (year1) |  |
| 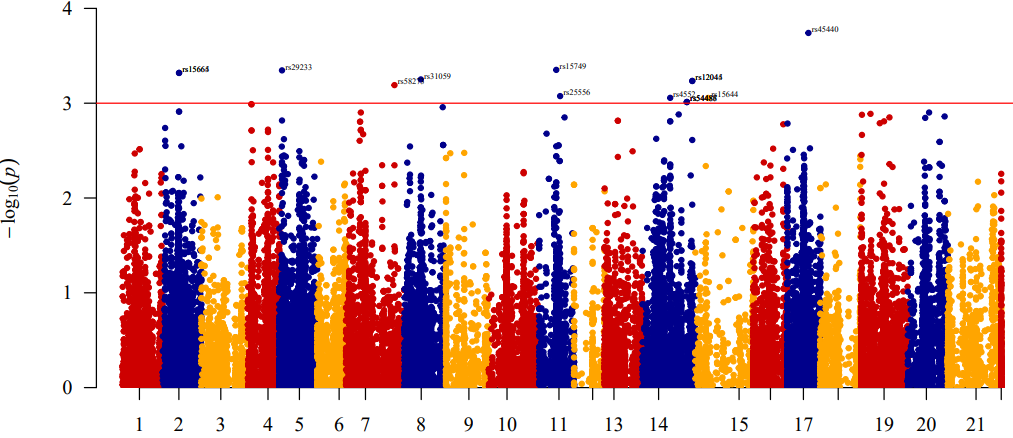 | 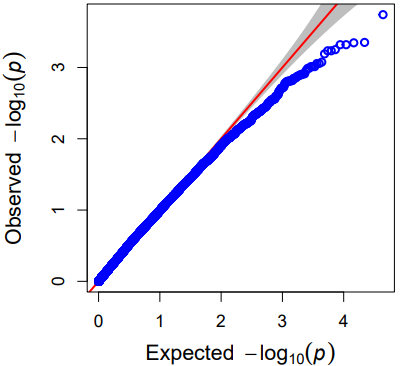 |

| DTF (year2) |  |
| --- | --- |
| 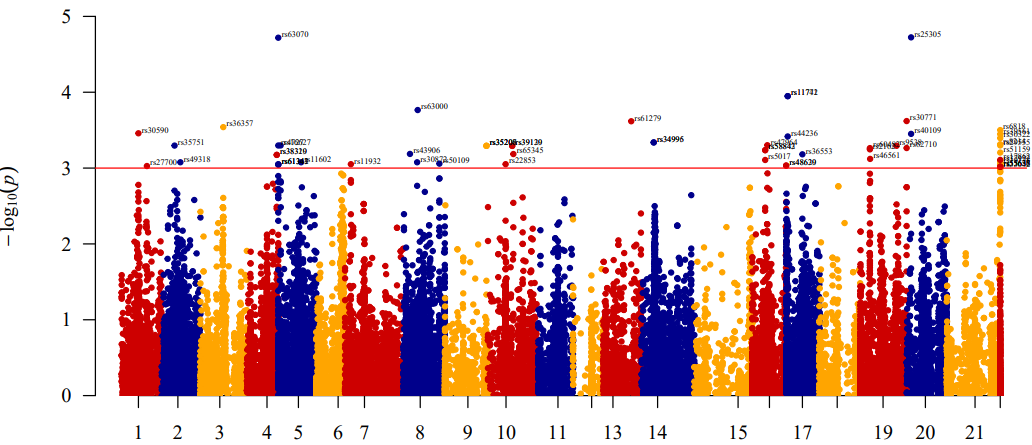 | 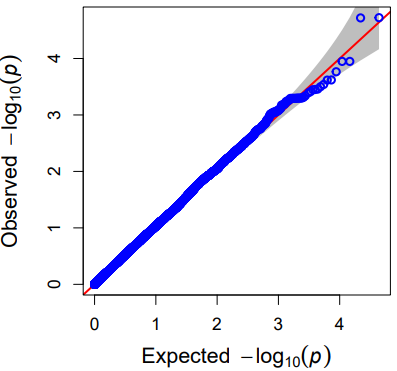 |
| DTH (year2) |  |
| 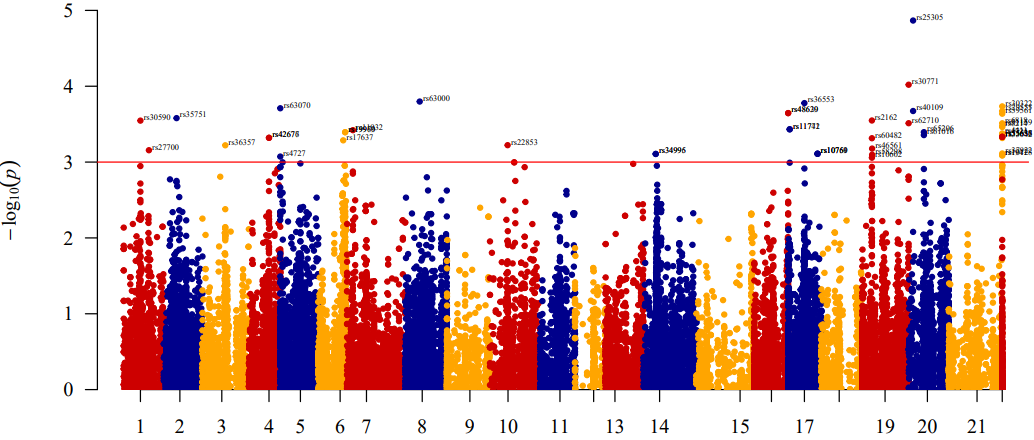 | 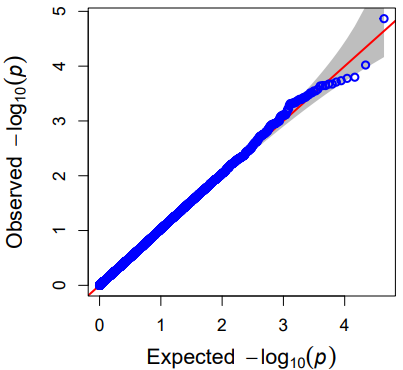 |
| DTM (year2) |  |
| 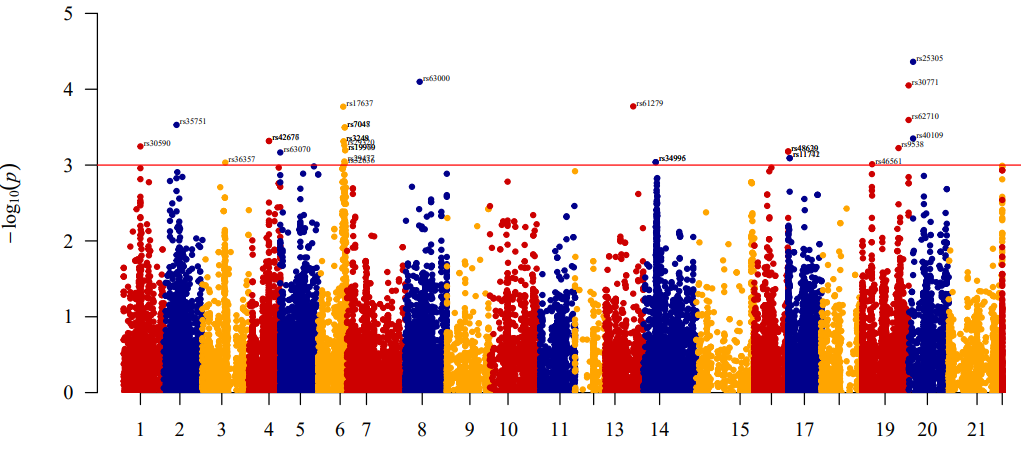 | 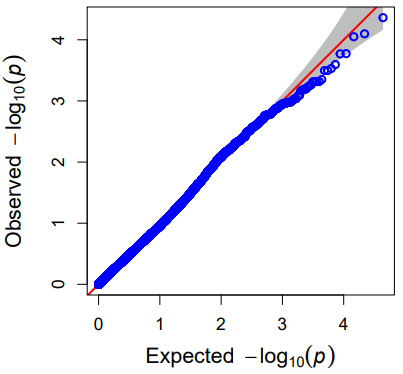 |
| GY (year2) |  |
| 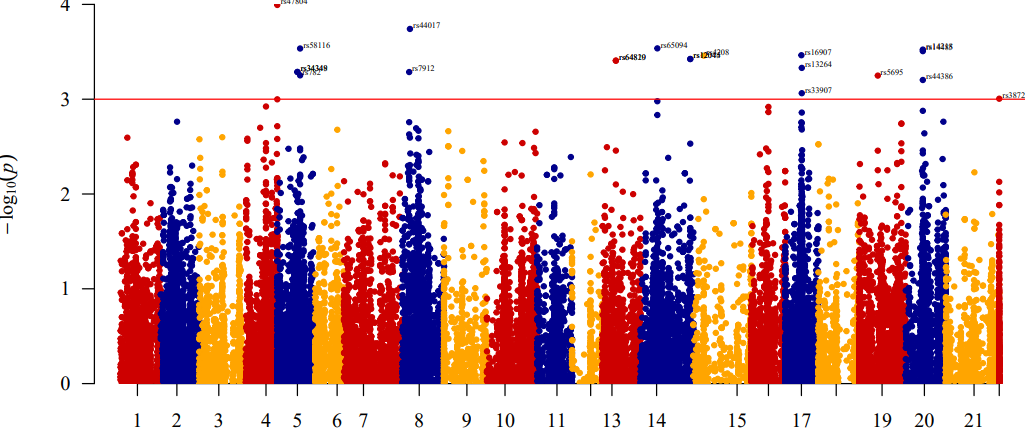 | 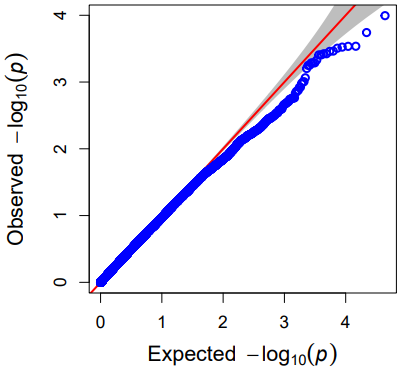 |
| ID1 (year2) |  |
| 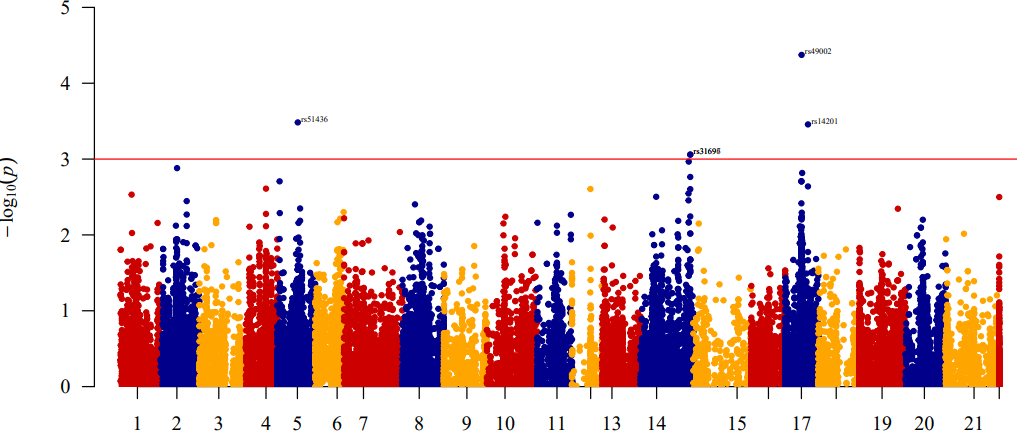 | 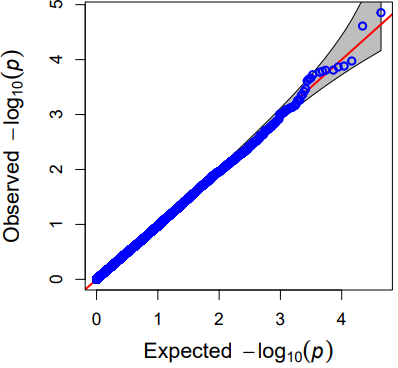 |
| ID2 (year2) |  |
| 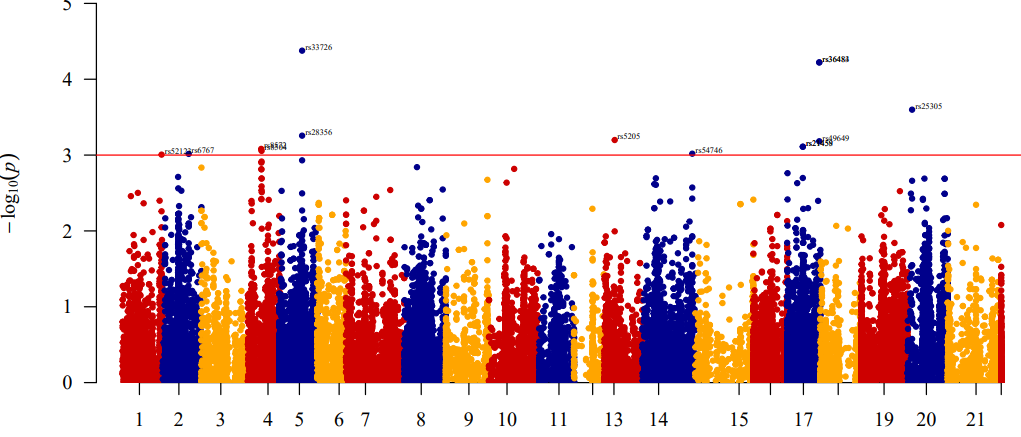 | 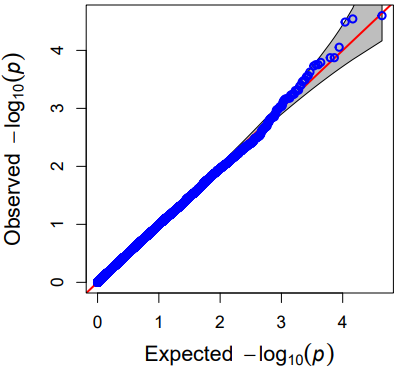 |
| IL1 (year2) |  |
| 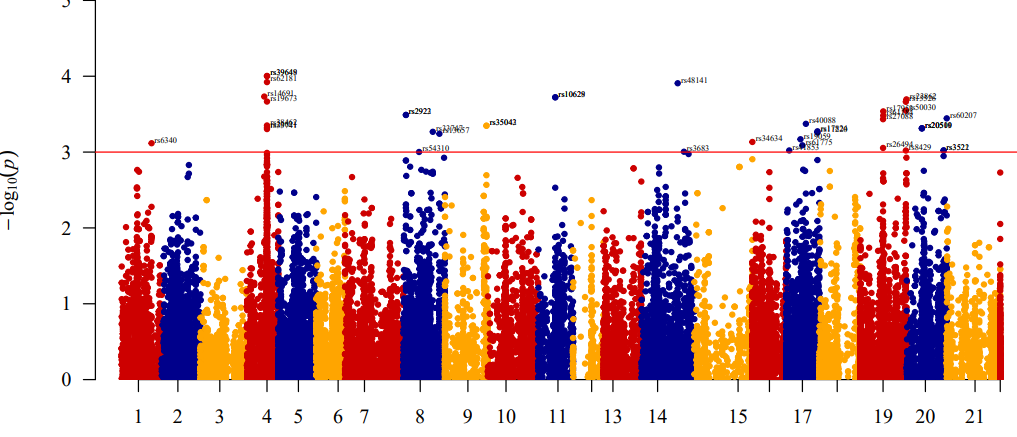 | 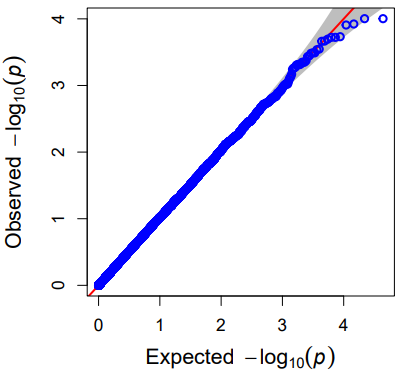 |
| IL2 (year2) |  |
| 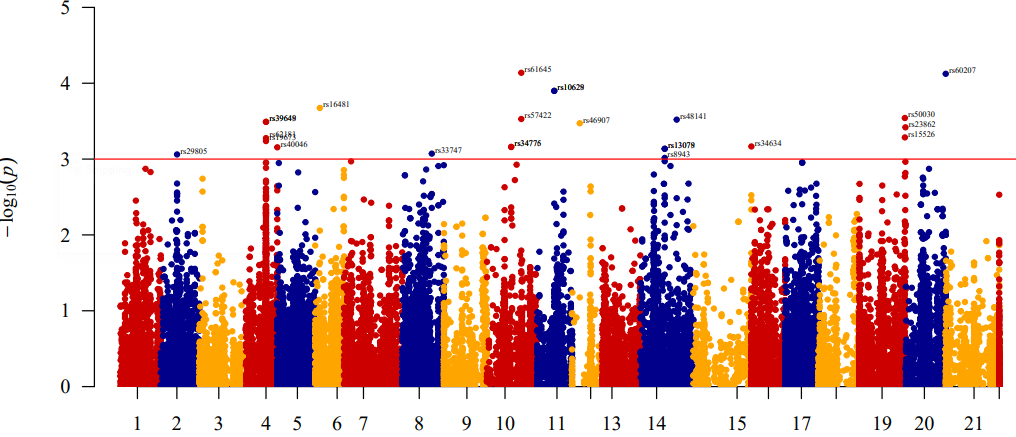 | 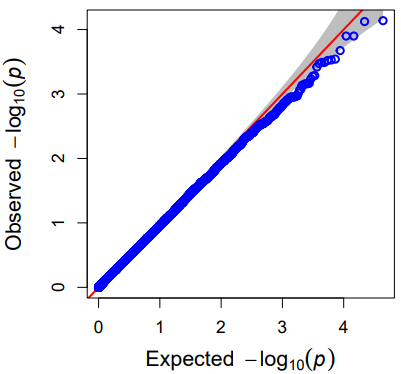 |
| NFN (year2) |  |
| 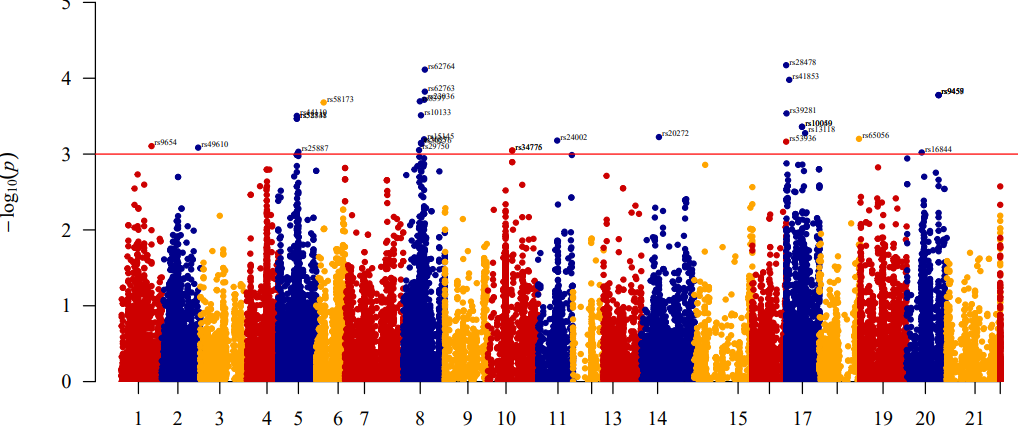 | 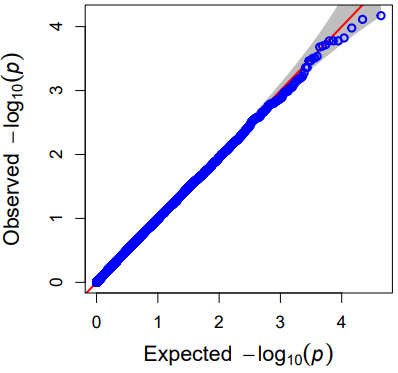 |
| PD (year2) |  |
| 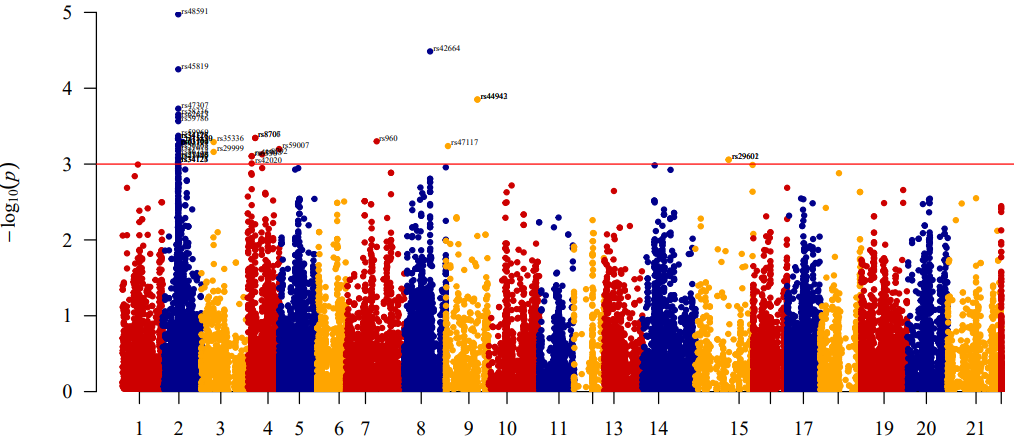 | 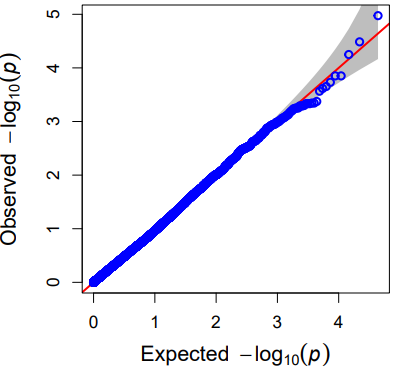 |
| PeD (year2) |  |
| 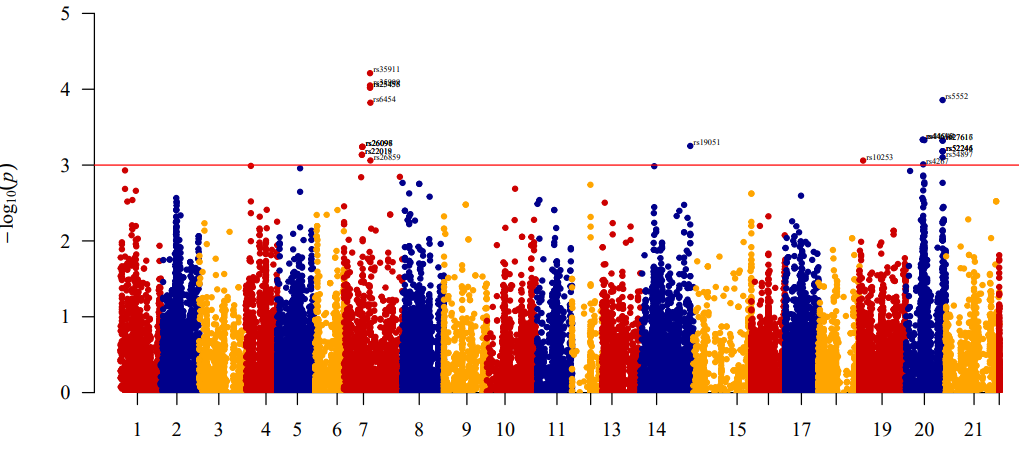 | 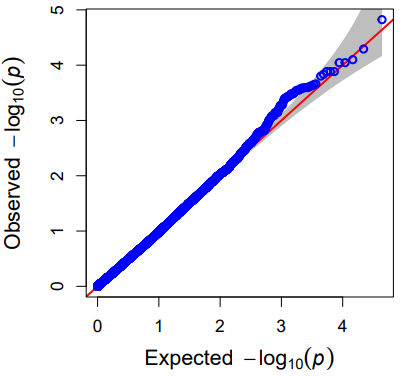 |
| PeL (year2) |  |
| 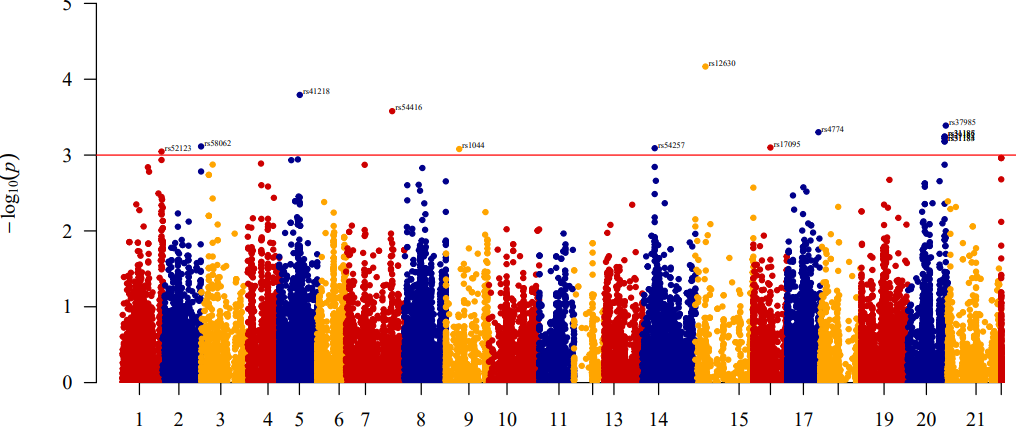 | 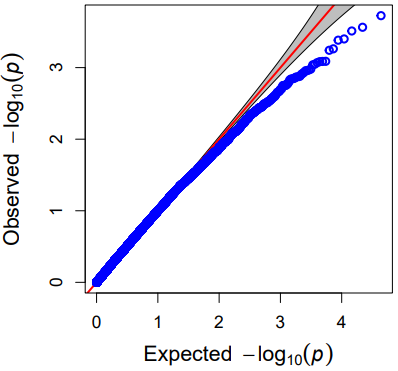 |
| PL (year2) |  |
| 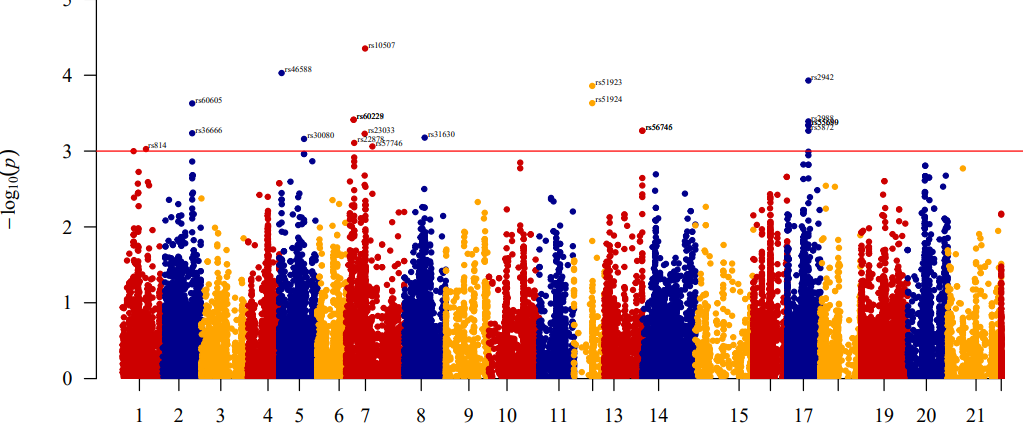 | 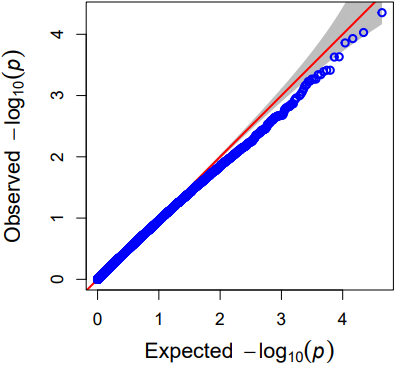 |
| SA (year2) |  |
| 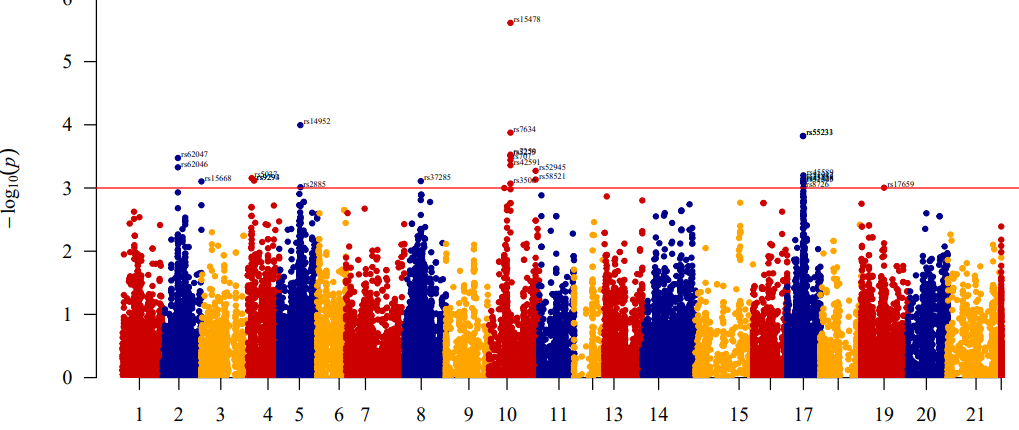 | 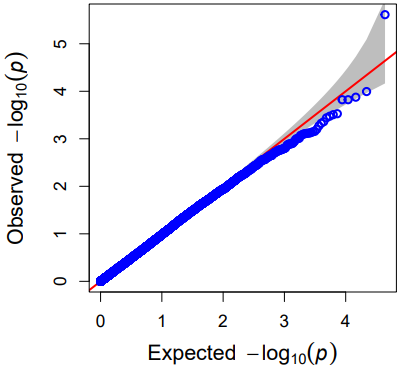 |
| SW (year2) |  |
| 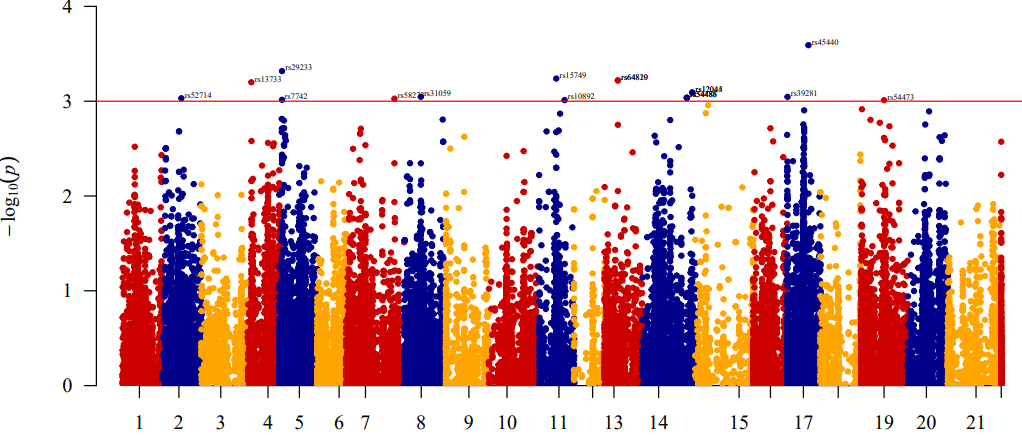 | 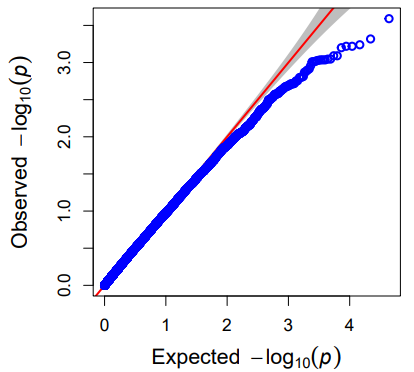 |

| DTF (pooled) |  |
| --- | --- |
| 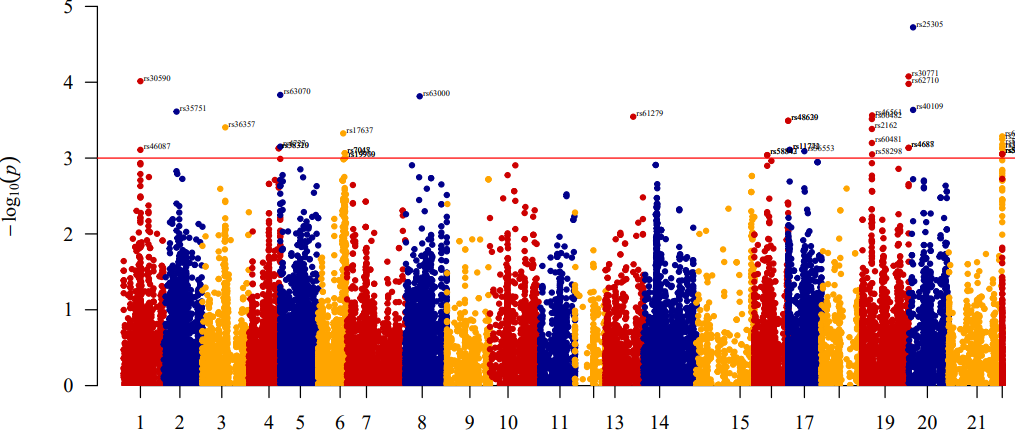 | 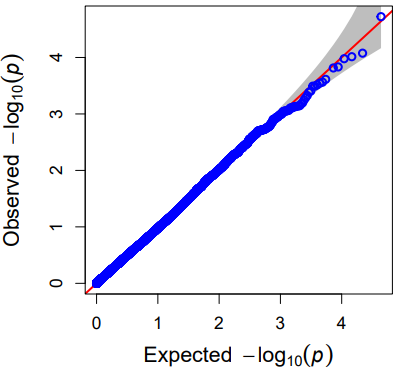 |
| DTH (pooled) |  |
| 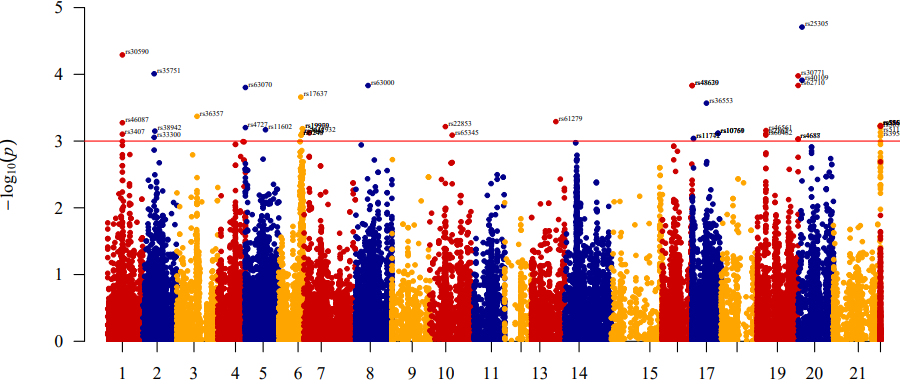 | 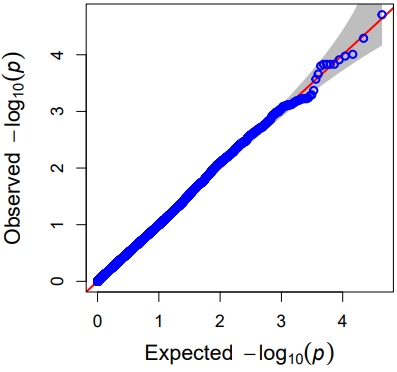 |
| DTM (pooled) |  |
| 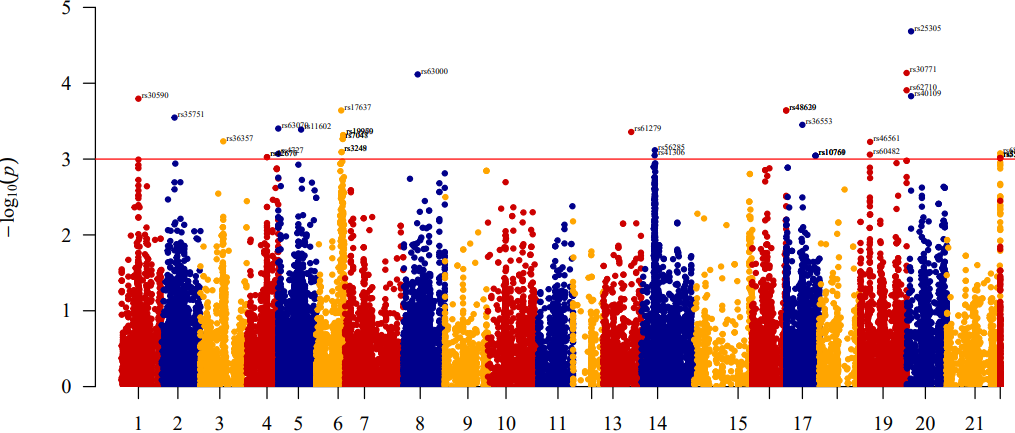 | 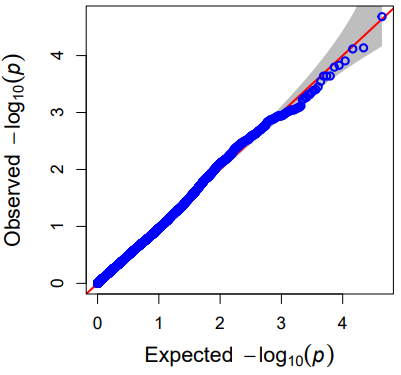 |
| GY (pooled) |  |
| 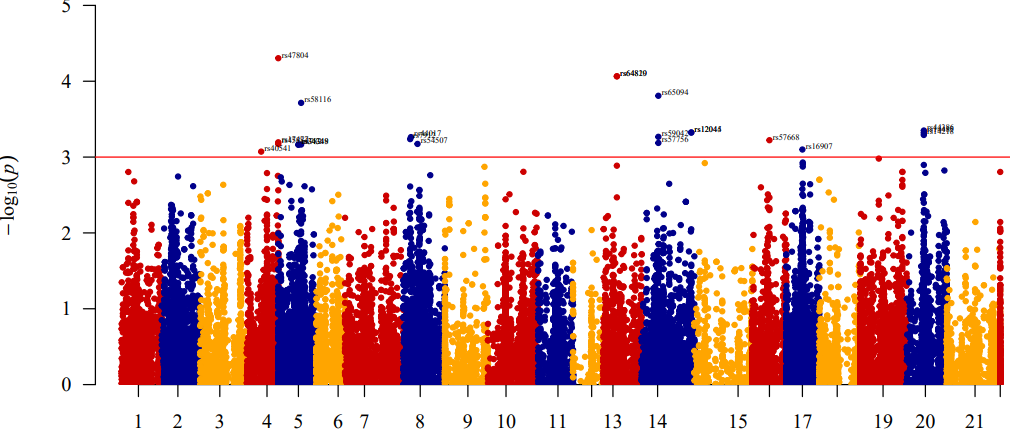 | 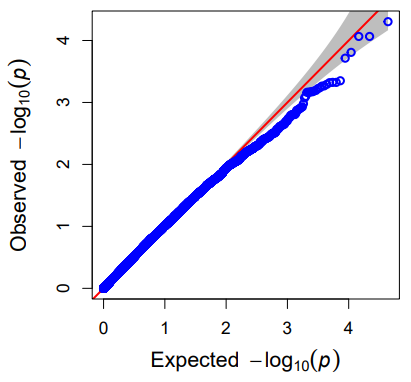 |
| ID1 (pooled) |  |
| 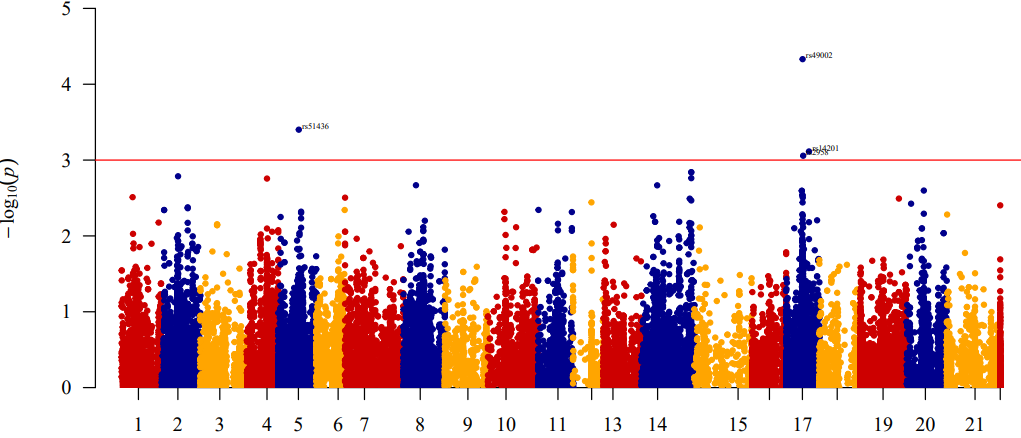 | 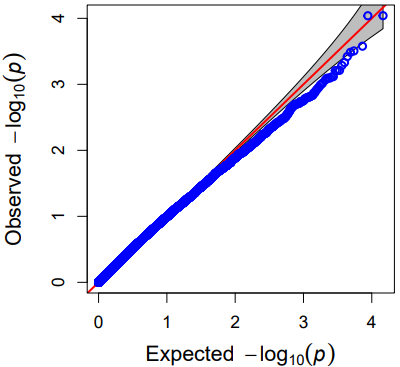 |
| ID2 (pooled) |  |
| 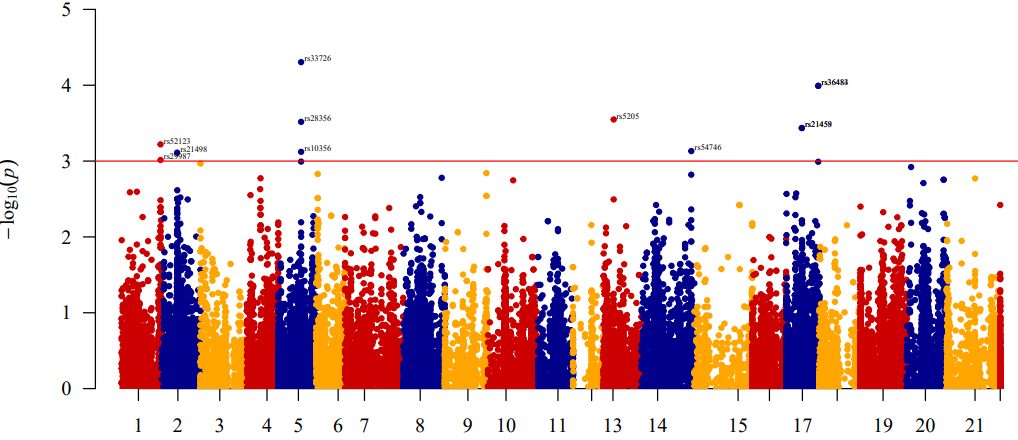 | 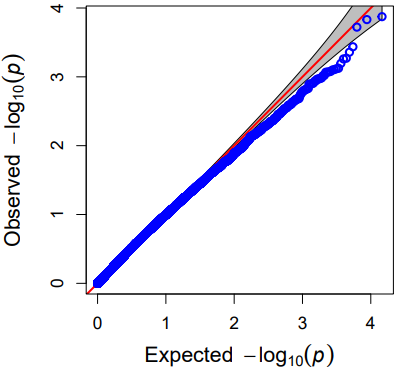 |
| IL1 (pooled) |  |
| 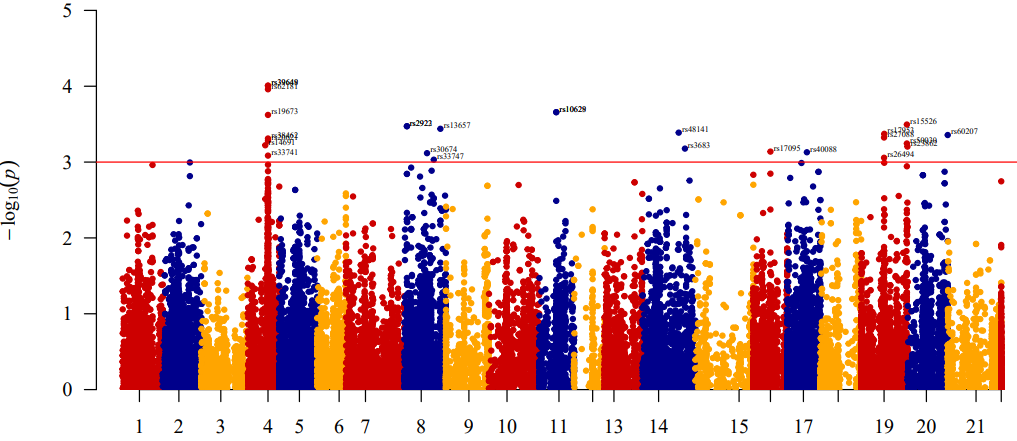 | 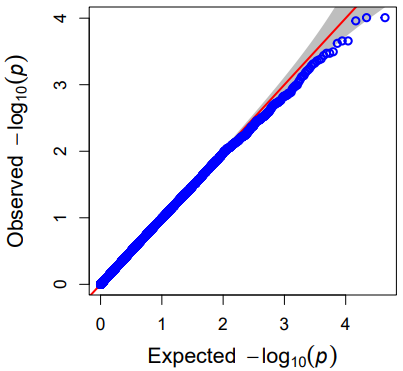 |
| IL2 (pooled) |  |
| 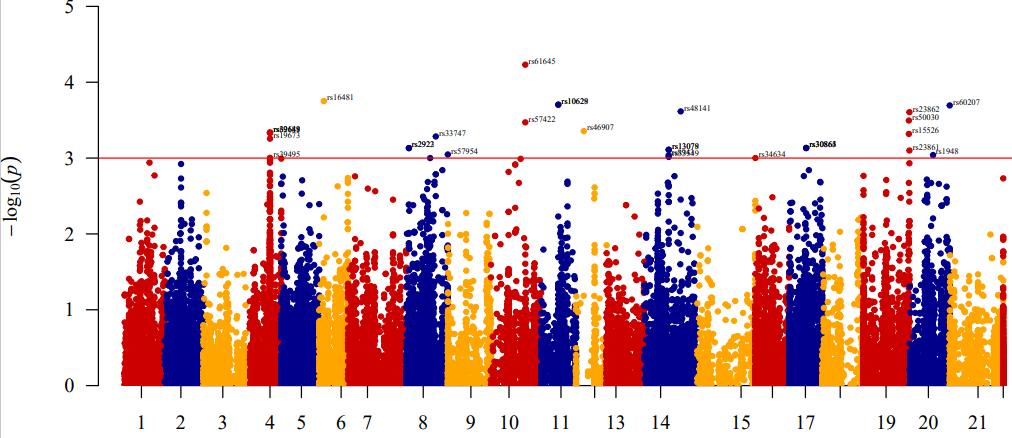 | 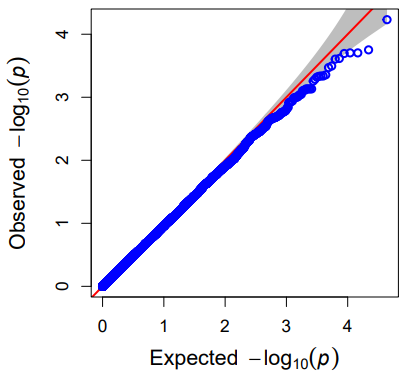 |
| NFN (pooled) |  |
| 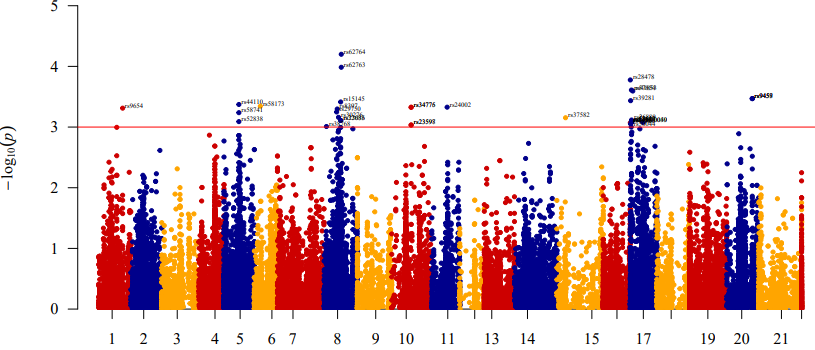 | 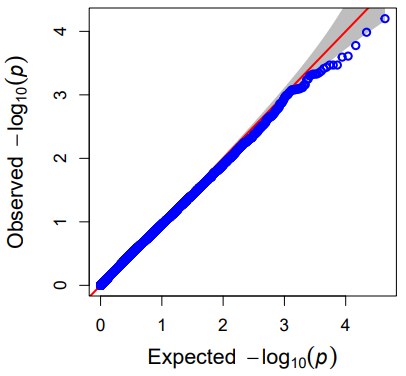 |
| PD (pooled) |  |
| 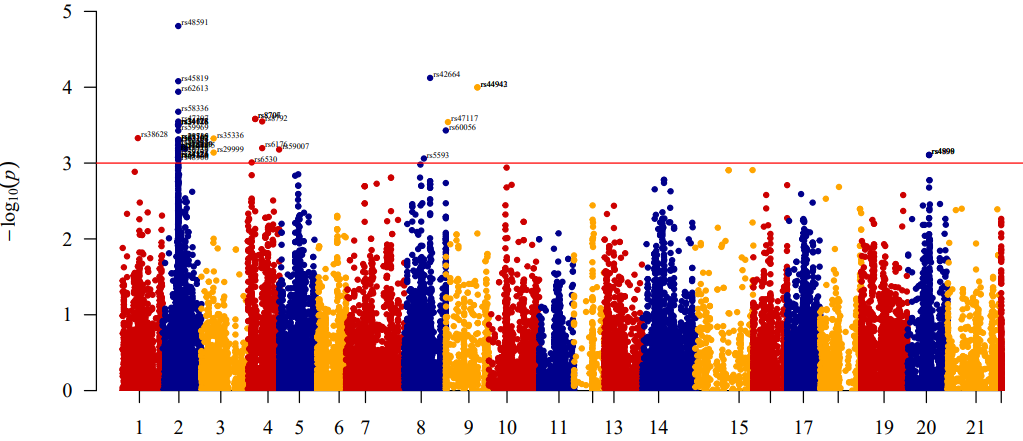 | 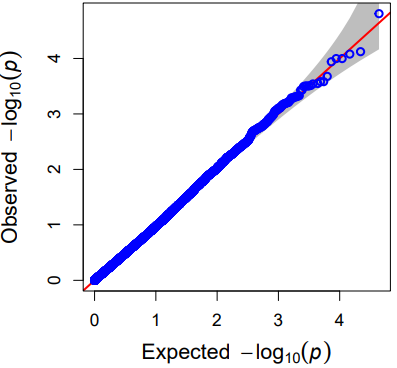 |
| PeD (pooled) |  |
| 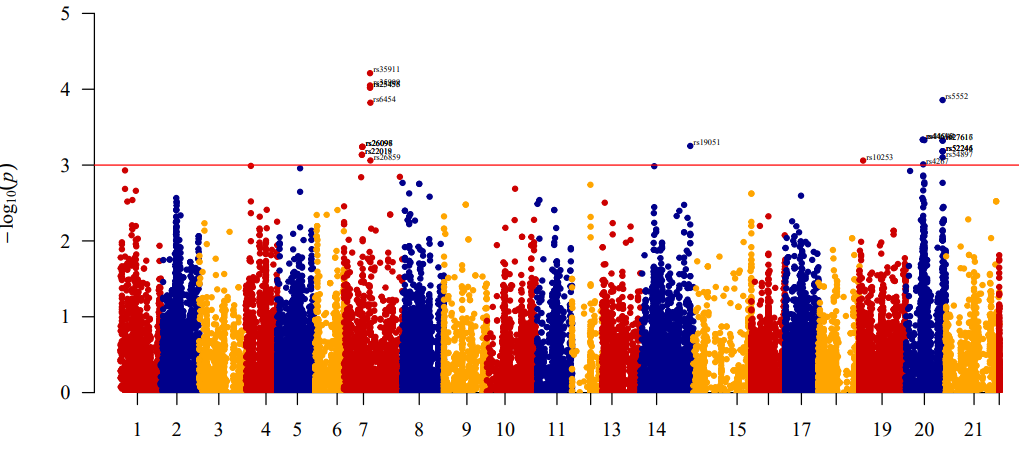 |  |
| PeL (pooled) |  |
|  |  |
| PL (pooled) |  |
|  |  |
| SA (pooled) |  |
|  |  |
| SW (pooled) |  |
|  |  |

**Supplementary Fig. 3** Manhattan and QQ-plots of highly associated haplotypes in Iranian wheat landraces and cultivars. X axis represents chromosomes: 1) 1A, 2) 1B, 3) 1D, 4) 2A, 5) 2B, 6) 2D, 7) 3A, 8) 3B, 9) 3D, 10) 4A, 11) 4B, 12) 4D, 13) 5A, 14) 5B, 15) 5D, 16) 6A, 17) 6B, 18) 6D, 19) 7A, 20) 7B, 21)7D.

Abbreviations: Lodged area (LA), crop angle of inclination (CAI), lodging score index (LS), plant height (PH), number of nodes (NFN), peduncle length (PL), penultimate length (PeL), internode length 1 (IL1), internode length 2 (IL2), peduncle diameter (PD), penultimate diameter (PeD), internode diameter 1 (ID1), internode diameter 2 (ID2), days to heading (DTH), days to flowering (DTF), days to maturity (DTM), spike weight (SW), spike area (SA) and grain yield (GY).


**Supplementary Fig. 4** The KEGG pathway of starch and sucrose metabolism.

**Supplementary Fig. 5** The KEGG pathway of Zeatin biosynthesis.

**Supplementary Fig. 6** The KEGG pathway of amino sugar and nucleotide sugar metabolism.

**Supplementary Fig. 7** The KEGG pathway of carbon metabolism

The pathway map without coloring is the original version that is manually drawn by in-house software called KegSketch. The other pathway maps with coloring are all computationally generated as summarized below.

Reference pathway: This is the original version; white boxes are hyperlinked to KO, ENZYME, and REACTION entries in metabolic pathways; they are hyperlinked to KO entries in non-metabolic pathways.

Reference pathway (KO): blue boxes are hyperlinked to KO entries that are selected from the original version.

Reference pathway (EC): blue boxes are hyperlinked to ENZYME entries that are selected from the original version.

Reference pathway (Reaction): blue boxes are hyperlinked to REACTION entries that are selected from the original version.

Organism-specific pathway: green boxes are hyperlinked to GENES entries by converting K numbers (KO identifiers) to gene identifiers in the reference pathway, indicating the presence of genes in the genome and also the completeness of the pathway.
